# Identification of TMEM263 as an ER-resident protein that controls early stages of lipid droplet biogenesis

**DOI:** 10.1038/s41467-026-78093-9

**Published:** 2026-09-28

**Authors:** Katy Yalci, Nafsika Chala, Joanna Moss, Connor MacDonald, Przemysław Zakrzewski, Phineas Smith, Rabia Sevil, Lana Willoughby, Seren Wonklyn, Thomas Andrews, Chrissy L. Hammond, Robin A. Corey, Bernadette Carroll

**Affiliations:** 1 School of Biochemistry and Biomedical Sciences, University of Bristol, Biomedical Science Building, Bristol, UK; 2 School of Psychology and Neuroscience, University of Bristol, Biomedical Science Building, Bristol, UK; 3 Present Address: Department of Physiology, Anatomy and Genetics, University of Oxford, Oxford, UK

**Keywords:** Endoplasmic reticulum, Fatty acids, Fat metabolism

## Abstract

Cell and organismal growth is controlled not only by the availability of nutrients, but also the ability to dynamically sense and respond to changes in metabolic demand. We have identified a protein, TMEM263 (also known as C12orf23) as a mechanistic link between growth and lipid metabolism. TMEM263 was discovered in a screen as an ER-resident protein, and we have characterised its role as both necessary and sufficient for lipid droplet formation. TMEM263 has two transmembrane domains that fold into a dynamic alpha hairpin which are essential for its localisation to the ER and to support lipid droplet accumulation. Functionally, we propose that TMEM263 can interact with and support the condensation of neutral lipids in a bilayer to promote lipid droplet formation and growth. Consistently, loss of TMEM263 in cells and in zebrafish significantly impairs lipid droplet accumulation. Loss of TMEM263 protein function in vivo is associated with organismal growth defects and proportional dwarfism and our study provides a mechanistic understanding linking these phenotypes to impaired lipid droplet biology.

## Abstract (web summary)

We reveal TMEM263, a gene associated with bone defects, is a helical transmembrane, ER-resident, COPI cargo protein that localises with nascent and mature lipid droplet markers. Loss of TMEM263 in cellulo and in vivo leads to decreased lipid droplet accumulation.

## Introduction

The ability to sense and appropriately respond to nutrients is essential for the control of cell and whole-body growth. Too few or too many nutrients is widely associated with human disease, and thus it is crucial we understand the molecular determinants controlling nutrient handling and nutrient homeostasis. An often-overlooked regulator of nutrient homeostasis and cellular growth is the endoplasmic reticulum (ER). It orchestrates the synthesis of proteins and lipids, facilitates proper protein folding, and serves as a store of intracellular calcium. To support these activities, the ER hosts a diverse array of resident proteins; ER-resident chaperones, for instance, are critical for the folding and maturation of secretory and membrane proteins, including growth factor receptors that directly influence cell growth and survival^1,2^. The localisation of such ER-resident proteins is tightly regulated, in part by the heptameric coatomer protein complex (or COPI). COPI recognises specific cargo motifs and facilitates both intra-Golgi trafficking and retrograde transport from the cis-Golgi back to the ER to prevent proteins being lost via the secretory pathway^3,4^. As such, COPI is essential for maintaining ER-Golgi homeostasis; its disruption results in widespread defects in vesicular transport, ER and Golgi disorganisation, mitochondrial dysfunction, and the aberrant accumulation of autophagosomes and lipid droplets^5–7^.

The ER also plays a central role in the dynamic sensing and response to metabolic challenges, including nutrient starvation and surplus^8^. For example, during nutrient starvation, recruitment of proteins such as WIPI2 and ATG16L to ER subdomains rich in P13KC3-dependent PI3P promotes the formation of phagophores, the precursors of autophagosomes, which support nutrient liberation and cell survival by delivering cytoplasmic cargo to the lysosome for degradation. One consequence of this degradation is an increase in free fatty acids from the breakdown of membranes^9^. Similar fatty acid accumulation can occur as a result of decreased utilisation, increased de novo biosynthesis or increased uptake from the environment via cell surface receptors such as CD36^10–13^. Fatty acids represent an important fuel source, while excess lipid accumulation can cause lipotoxicity, cytotoxic stress and cell death^10,11^. Therefore, as a protective storage mechanism, fatty acids are converted by ER-resident enzymes into neutral lipids, which are stored in lipid droplets. Specifically, enzymes such as diacylglycerol O-acyltransferase 1 (DGAT1) and acyl-CoA:cholesterol acyltransferases (ACAT1/2) esterify fatty acids and cholesterol, respectively, generating triacylglycerols (TAGs) and cholesterol esters, which accumulate within the ER phospholipid bilayer. When the neutral lipid accumulation surpasses a critical concentration (~3–5 mol%), they undergo phase-separation into a lipid ‘lens’^14–17^. The growing lipid core grows asymmetrically towards the cytoplasm in a process regulated by the biophysical properties of the ER bilayer, such as membrane tension and phospholipid composition^10,18^. The subsequent monolayer-enclosed lipid droplet remains functionally and physically connected to the ER by extensive membrane contact sites. These contact sites facilitate the exchange of proteins and lipids to allow the rapid response to changes in cellular metabolic demand, leading to lipid droplet expansion, shrinkage or degradation^10–13,19^.

Several ER-resident proteins have emerged as key regulators of lipid droplet biogenesis, acting in concert with metabolic enzymes. Seipin/BSCL2 is a multimeric complex that localises at ER–lipid droplet contact sites to coordinate the nucleation and growth of lipid droplets by organising lipid lenses and recruiting metabolic enzymes^20–22^. FIT2, another ER-resident protein, is implicated in early lipid droplet formation, potentially through modulating membrane curvature^23^. Given the tight and dynamic spatial relationship between the two organelles, there are likely to be many more ER-resident proteins involved in lipid droplet biology.

Using unbiased proteomics to reveal ER-resident regulators of mammalian growth, we identified TMEM263/C12orf23, mutation of which is associated with organismal growth defects and dwarfism. We determined TMEM263 is a COPI cargo and a resident of the ER, where it regulates early steps in lipid droplet formation. Specifically, we show that overexpressed TMEM263 localises to nascent lipid droplets and is sufficient to induce the accumulation of enlarged, clustered lipid droplets, particularly upon lipid feeding. Modelling indicates that TMEM263 has two transmembrane (TM) domains connected via a linker in the ER lumen, and experimentally, we demonstrate that the TM domains are essential for TMEM263 localisation and function. Molecular dynamics simulations indicate that TMEM263 can interact with and support the condensation of neutral lipids in a bilayer, and mutation of key structural and lipid-interacting residues impairs EGFP-TMEM263-induced lipid droplet formation in cells. Consistently, loss of endogenous TMEM263 in cells and in zebrafish significantly reduces lipid droplet number following lipid feeding. Together, our data indicate TMEM263 is required for efficient lipid droplet formation in vitro and in vivo and provides a mechanistic understanding for the in vivo phenotypes observed upon TMEM263 loss*-*of-function^24–28^.

## Results

**Fig. 1:**
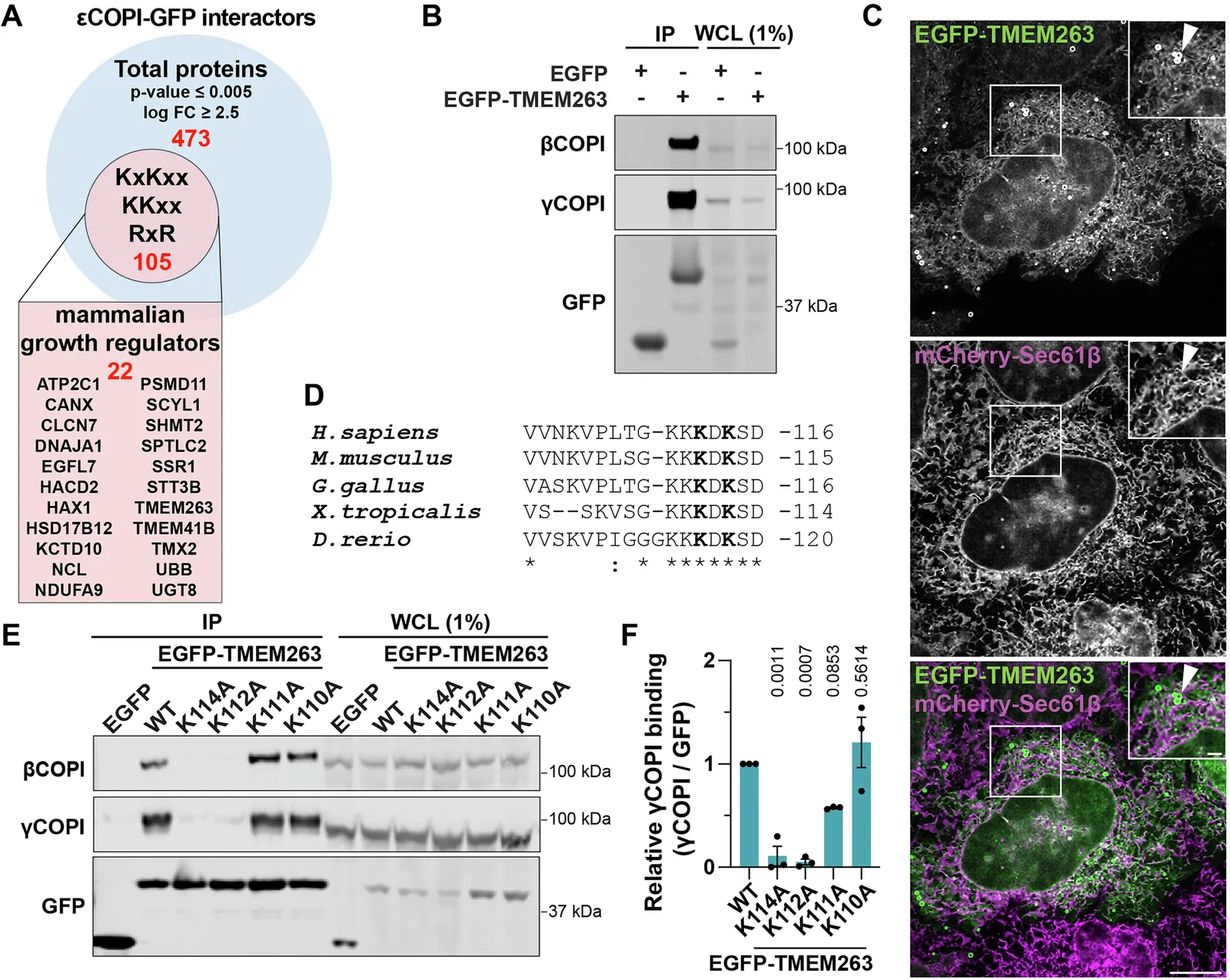
TMEM263 is a COPI cargo protein. **A** Identification of COPI cargo. HeLa cells stably expressing GFP or εCOPI-GFP were subject to GFP immunoprecipitation and analysis by TMT mass spectrometry. COPI cargoes were defined as proteins with a log_2_ fold change (FC) ≥ 2.5 and p-value ≤ 0.005 εCOPI-GFP / GFP (*n* = 4). Proteins were further filtered for those containing COPI-interacting motifs, either KxKxx or KKxx (at the C-terminus) or RxR (within the last 30 amino acids of the C-terminus), and associated with mammalian growth regulation. **B** TMEM263 is a COPI-interacting protein. HeLa cells expressing EGFP-TMEM263 were subject to GFP immunoprecipitation and western blot to detect endogenous COPI components, γCOPI and βCOPI, *n *= 2, both biological replicates had similar results. **C** TMEM263 is localised to the ER. HeLa cells transiently expressing EGFP-TMEM263 and stably expressing mCherry-Sec61β (ER) were imaged live. The white arrowhead points to EGFP-TMEM263 vesicular structures. **D** TMEM263 contains a canonical COPI interacting motif. ClustalW alignment highlighting a conserved canonical COPI motif, KxKxx in the terminal five amino acids of TMEM263. **E**, **F** Mutation of the COPI-binding motif inhibits TMEM263-COPI interaction. HEK293T cells overexpressing EGFP-TMEM263 wild-type or mutants, as indicated, were subject to GFP immunoprecipitation and western blot to detect endogenous COPI components, γCOPI and βCOPI (**E**). The interaction between EGFP-TMEM263 and γCOPI was quantified (**F**). Unless stated otherwise, *n* = 3 and error bars represent Standard Error of the Mean (SEM). An ordinary one-way ANOVA with Dunnett’s multiple comparisons test was performed. Scale bars are 10 μm and 2 μm for zoomed images.

The coatomer, or COPI protein complex, is essential for the maintenance of ER homeostasis and healthy cell growth. COPI is a large stable protein complex of seven subunits (α, β, β’, γ, δ, ε, ζ) which is recruited to Golgi membranes, via Arf1-GTP, to promote vesicle biogenesis and budding to support retrograde cargo transport both from cis-Golgi back to the ER and within the Golgi (trans- to cis-Golgi membranes). Cargo proteins interact with outer-COPI coat subunits, which consist of α-COP, β’-COP and ε-COP predominantly via the short consensus motifs, KxKxx or KKxx, present in the very C-terminus of their cytoplasmic tails^29^. In addition, less well-characterised motifs, including RxR and Φ-(K/R)-X-L-X-(K/R), are recognised by β-COPI / δ-COPI and δ-COPI / ζ-COPI, respectively^4^. To identify ER-resident COPI cargo which are important for controlling cell growth, we overexpressed a component of the outer-coat COPI complex, εCOPI-GFP in HeLa cells and initially confirmed it integrated properly into complex with other COPI machinery and localised to the ER and Golgi as expected (Supplementary Fig. 1A, B). We subsequently carried out GFP immunoprecipitation and proteomic analyses, which identified 473 high-confidence (log_2_ FC ≥ 2.5, *p*-value ≤ 0.005) binding partners for COPI, including 105 that contain the canonical COPI binding motifs, KxKxx, KKxx, or RxR (Fig. 1A, Supplementary Fig. 1C and Supplementary Data 1). We further filtered the results using the online MGI database^30,31^ to identify proteins that have been implicated in mammalian growth. This approach produced a list of 22 proteins, several of which are also implicated in lipid metabolism (Fig. 1A and Supplementary Fig. 1C). These include UGT8, which is a ceramide galactosyltransferase and HSD17B12, which is a 17-beta-hydroxysteroid dehydrogenase. Other interesting hits include TMEM41B, which is a lipid scramblase that has been implicated in the biogenesis of lipid droplets and autophagosomes from the ER membrane^32,33^, and TMEM263. Also known as C12orf23, TMEM263 is a vertebrate-specific 12 kDa protein with a poorly characterised function. Loss-of-function mutations in TMEM263 have been associated with bone and growth defects in chickens, mice, and humans^24–28^. Mechanistically, human GWAS studies implicate *TΜΕΜ263* variants in bone mineral density and osteoblast function^24,25^. The defects in bone growth observed in *Tmem263* knock-out mice were associated with reduced growth hormone receptor expression, and a subsequent reduction in circulating IGF-1 levels^26^, although there were no observed differences in circulating IGF-1 in mutant chicken strains^27,34,35^. Thus, it is unclear at present whether these phenotypes were a direct result of TMEM263 loss, and the structure and molecular function of TMEM263 remain largely unknown. Interestingly, TMEM263 has also been validated as a high-confidence, but uncharacterised lipid droplet protein^12,36^.

To confirm that TMEM263 is an ER-resident protein and COPI cargo, we validated the COPI-TMEM263 interaction by immunoprecipitation using εCOPI-GFP and mCherry-TMEM263 (Supplementary Fig. 1D). The reciprocal immunoprecipitation using EGFP-TMEM263 as bait further confirmed a robust interaction with endogenous COPI (Fig. 1B). Furthermore, as would be expected of COPI cargo, EGFP-TMEM263 localised to the ER (Fig. 1C and Supplementary Fig. 1E, F), Golgi (Supplementary Fig. 1E, F) and endogenous and overexpressed COPI subunits (the latter at small vesicular structures and at a region consistent with the Golgi, see arrowheads, Supplementary Fig. 1G, H). The C-terminal five amino acids of TMEM263 encode the canonical COPI binding motif, KxKxx (**K**D**K**SD) (Fig. 1D), and mutation of lysines in this motif (amino acids 112 and 114) abolished the interaction with COPI (Fig. 1E, F). As a control, mutation of two upstream lysine residues (K110 and K111) had no significant impact on TMEM263-COPI binding (Fig. 1E, F). These data validate TMEM263 a bona fide COPI cargo protein and ER-localised protein.

**Fig. 2:**
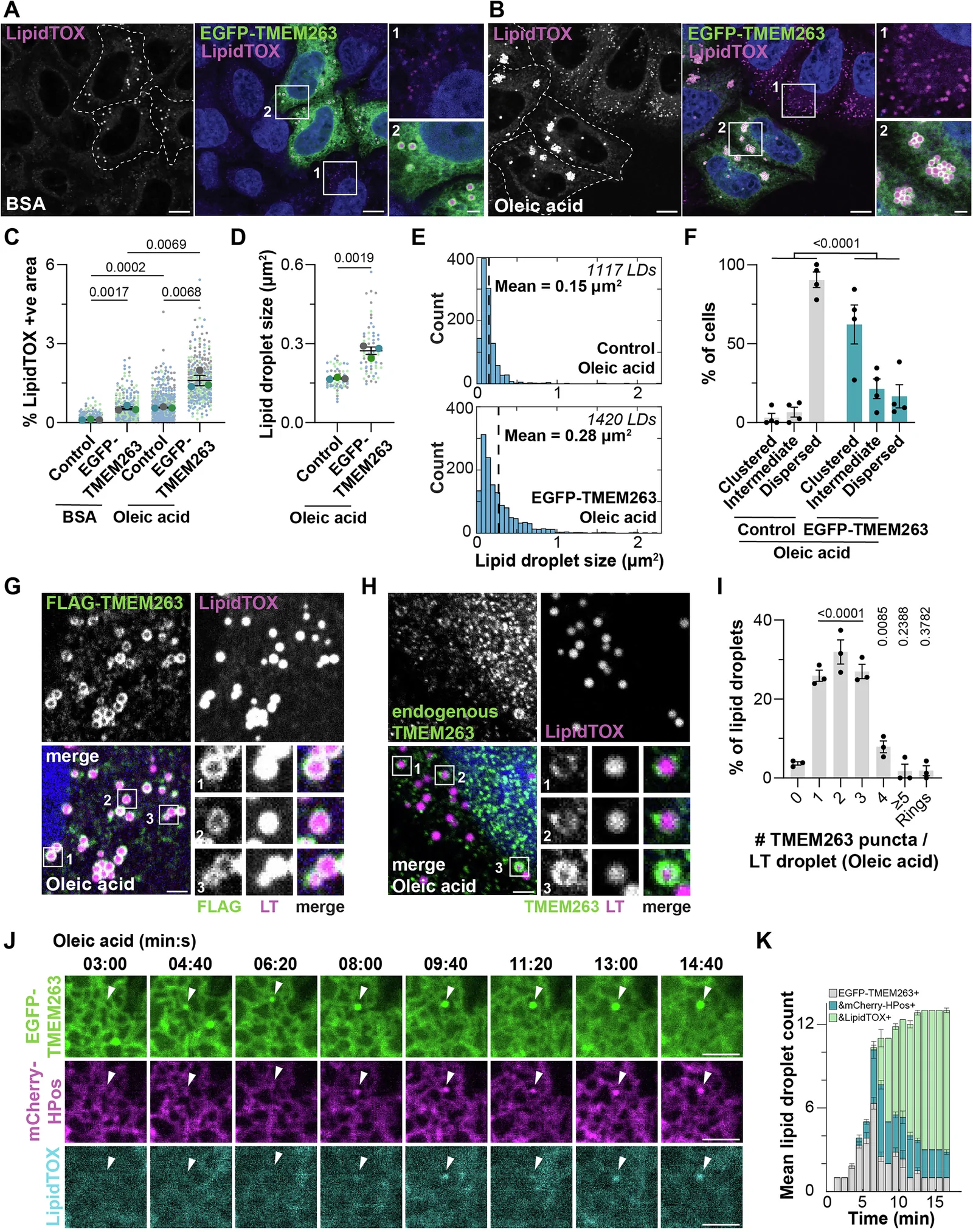
TMEM263 is localised to nascent lipid droplets. **A**, **B** TMEM263 expression induces the accumulation and clustering of lipid droplets. HeLa cells expressing EGFP-TMEM263 were incubated for 16 h with BSA (**A**) or 30 µM oleic acid (**B**), fixed and stained with LipidTOX. White dotted lines highlight EGFP-transfected cells, which were quantified relative to neighbouring untransfected controls in the same field of view. **C–E** The % LipidTOX positive area/cell was quantified from cells treated as in (**A**, **B**). *n* = 3. **C** The mean size of lipid droplets per cell using the LipidTOX signal was quantified from cells treated as in (**B**). *n* = 3, single plane images were used (**D**). Histograms of individual lipid droplet size of 20 control cells and 21 cells expressing EGFP-TMEM263 using a maximum z-projection are shown (**E**). **F** Cells treated as in A-B were scored as having a clustered, intermediate, or dispersed distribution of EGFP-TMEM263-positive lipid droplets. *n* = 4, Cochran-Mantel-Haenszel chi-squared test with Haldane-Anscombe and Bonferroni corrections was performed. **G** HeLa cells expressing FLAG-HA-TMEM263 were treated as in (**B**), immunostained with an antibody against FLAG and stained with LipidTOX. **H**, **I** Endogenous TMEM263 localises to lipid droplets. HeLa cells were treated as in (**B**), immunostained with an antibody againstTMEM263 and stained with LipidTOX (**H**). Quantification of the number of TMEM263-positive puncta per lipid droplet, chi-squared tests with Bonferroni correction were performed (**I**). HeLa cells transfected with EGFP-TMEM263 and mCherry-HPos, stained with LipidTOX and imaged live for 18 min after oleic acid addition, arrowheads point to the formation of a LipidTOX-positive lipid droplet (**J**). Quantification of the appearance time of each signal, 14 puncta, across 5 cells in 3 independent experiments were quantified. Error bars represent standard deviation (SD) (**K**). Unless stated otherwise, *n* = 3. Scale bars are 10 µm and 2 μm for the zoomed in images and for **G**, **H**, and **J**. Unless stated otherwise, bars extend to the mean and error bars represent SEM. For (**C**, **D**), colour-coded individual data points correspond to individual cells from different experiments, lines show mean values, error bars represent SEM and two-tailed unpaired Student’s *t* tests were performed.

We observed that upon overexpression, in addition to EGFP-TMEM263 localisation to ER, it also induced the accumulation of vesicular structures (see arrowhead, Fig. 1C). EGFP-TMEM263 positive ‘puncta’ were observed in ~80% transfected HeLa cells, which ranged from tiny puncta to large, hollow vesicles (Fig. 1C and Supplementary Fig. 2A). Given the previous publication of TMEM263 as a high-confidence lipid droplet protein, we stained cells with the neutral lipid dye, and mature lipid droplet marker, LipidTOX. EGFP-TMEM263-positive puncta were almost exclusively labelled with LipidTOX (Supplementary Fig. 2B). Overexpression of EGFP-TMEM263 promoted a significant increase in LipidTOX-positive area in both control (BSA) and lipid-fed (oleic acid; OA) HeLa cells (Fig. 2A–C). In lipid-fed conditions, this is mediated by a significant increase in lipid droplet size (from 0.15 μm^2^ in control to 0.28 μm^2^, Fig. 2D, E), but not in lipid droplet number (Supplementary Fig. 2C–E) compared to control cells. The intracellular and inter-cellular variations of LD sizes is displayed in the histograms in Supplementary Fig. 2D, E. Furthermore, in OA-fed cells, we observed that EGFP-TMEM263 induced a striking and significant shift in the cytoplasmic distribution of lipid droplets. Specifically, over 50% of analysed cells showed a distinctive clustered, or ‘bunch-of-grapes’ phenotype compared to untransfected controls (Fig. 2B, F). The lipid droplet protein, perilipin 2 (PLIN2), co-localised with EGFP-TMEM263 at the membrane surrounding the LipidTOX-positive core (Supplementary Fig. 3A, B) and a similar localisation of additional lipid droplet proteins, LPCAT1 and LPCAT2, further confirms TMEM263 overexpression induces the accumulation of canonical lipid droplets (Supplementary Fig. 3C)*.* Lipid droplet accumulation and clustering was also observed upon overexpression of EGFP-TMEM263 in mouse embryonic fibroblasts (MEFs) and retinal epithelial cells (RPE-1) in both BSA and OA-fed conditions (Supplementary Fig. 3D–I). To ensure the large EGFP-tag is not affecting the localisation of TMEM263 to lipid droplets, we overexpressed FLAG-HA-TMEM263 (Fig. 2G) as well as stained for endogenous TMEM263 (Fig. 2H and Supplementary Fig. 3J for endogenous antibody validation). In both cases, TMEM263 localisation to LipidTOX-positive lipid droplets was observed. Quantitative analysis of endogenous TMEM263 immunofluorescence identified it localises to discrete puncta, typically 1-3 puncta per LipidTOX-positive lipid droplet, while ‘rings’ of TMEM263 around the LipidTOX core, similar to the observed distribution of EGFP-TMEM263, are occasionally observed (Fig. 2H, I). Overexpression of EGFP-TMEM263 in cells treated with shRNA targeting the UTR of TMEM263 resulted in a similar localisation to LipidTOX-positive lipid droplets (Supplementary Fig. 3K).

Given that TMEM263 is an ER-resident protein (Fig. 1C) and its overexpression is sufficient to drive the accumulation of mature lipid droplets, as labelled with LipidTOX (Fig. 2A–F), we reasoned that it may function at the early stages of lipid droplet formation. To test this, we initially used confocal and STED microscopy to ask whether TMEM263 colocalised with seipin/BSCL2, a key ER-resident regulator of lipid droplet biogenesis^10^ (Supplementary Fig. 3L–N). We did not observe any significant colocalisation of the two proteins (Supplementary Fig. 3L–N). Next, we co-expressed EGFP-TMEM263 with mCherry-HPos, which labels ER and nascent lipid droplets, before neutral lipids accumulate to levels sufficient to stain positive for LipidTOX^37^. EGFP-TMEM263 shows a clear ER localisation immediately after OA feeding (Fig. 2J and Supplementary Movie 1). Significant new EGFP-positive puncta emerged ~ 6.5 min after addition of OA (average: 6:36 ± 0:46 min). These puncta were initially negative for mCherry-HPos, but acquired mCherry signal ~ 8 min after addition of OA (average: 8:08 ± 0:41 min). The average time lapsed between the emergence of EGFP-TMEM263 puncta and the emergence of mCherry-HPos signal was 1:07 ± 0:21 min. The EGFP-TMEM263^+^ mCherry-HPos^+^ puncta then acquired LipidTOX staining ~ 10 min after addition of OA (average: 10:13 ± 0:57 min) (Fig. 2J, K and Supplementary Movie 1). Altogether, these data, identify TMEM263 as an ER-resident protein that localises to nascent and mature lipid droplets. We show that TMEM263 overexpression is sufficient to promote the accumulation of enlarged lipid droplets with a propensity to cluster. These data are consistent with the identification of TMEM263 as a high-confidence, but an uncharacterised lipid droplet protein in the literature^12,36^.

**Fig. 3:**
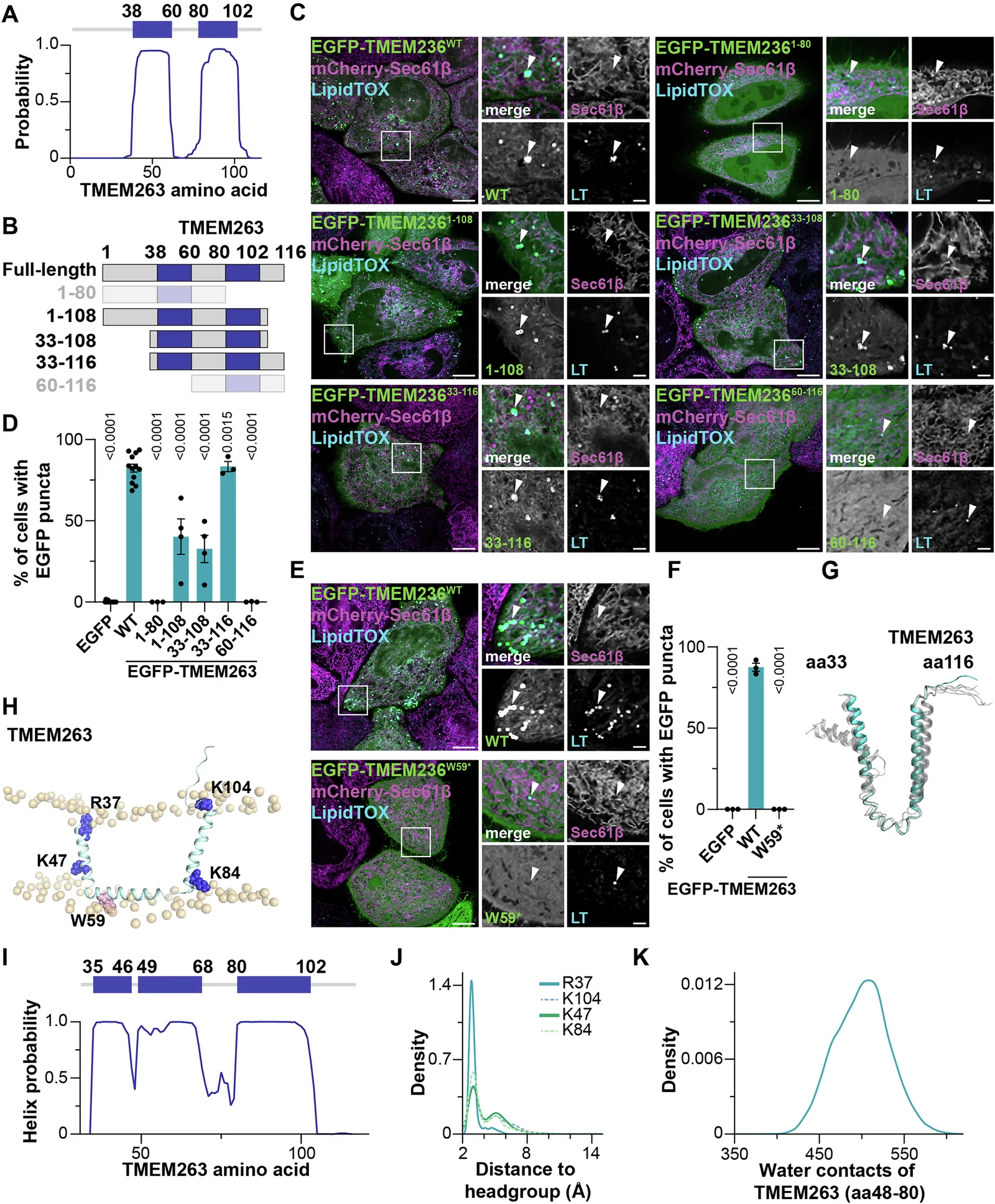
TMEM263 has two transmembrane helixes. **A** TMEM263 is an integral membrane protein. TMHMM predicts two transmembrane domains, amino acids 38–60 and 80–102 (dark blue) in *H. sapiens* TMEM263. **B** Diagram of TMEM263 protein truncations generated in this study. **C**, **D** TMEM263 transmembrane domains are required for its localisation. HeLa cells stably expressing mCherry-Sec61β (ER) were transfected with EGFP-TMEM263 and stained with LipidTOX (**C**). HeLa cells were transfected with EGFP or EGFP-TMEM263 truncations, fixed and scored for the presence of EGFP-positive puncta (**D**). **E**, **F** TMEM263 mutation associated with dwarfism impairs TMEM263 localisation. HeLa cells stably expressing mCherry-Sec61β were transfected with EGFP-TMEM263 and stained with LipidTOX (**E**). HeLa cells were transfected as indicated, fixed and scored for the presence of EGFP-positive puncta (**F**). **G** TMEM263 folds into a hairpin structure. Overlay of the top 5 ranked of 40 AlphaFold2 models of TMEM263^33−116^. Rank 1 is coloured cyan, and ranks 2–5 are coloured grey. **H** Representative snapshot of TMEM263 from atomistic MD. The protein (cyan) is embedded in a lipid bilayer, with phosphate headgroups depicted as orange spheres. The basic residues (R37, K47, K84, K104; blue spheres) are used as reference points for further study. W59 is shown as a pink sphere. **I** Per-residue helix probability from atomistic MD simulations of TMEM263, calculated as the fraction of frames assigned as helical by DSSP analysis, averaged across five independent repeats. **J** Distance distributions between basic residues and phosphate headgroups of the upper and lower bilayer leaflets from atomistic MD simulations. Kernel density estimates the minimum distance between basic residues and phosphate headgroups of the upper and lower leaflets are shown. **K** Distribution of water contacts (TIP3) within 0.35 nm for residues in the range of 48–80 from atomistic MD simulations. Unless stated otherwise, *n* = 3. Scale bars are 10 µm and 2 μm for zoomed images. Bars extend to the mean, and error bars represent SEM. For (**D**, **F**) *n* = 3, Cochran-Mantel-Haenszel chi-squared tests with Haldane-Anscombe and Bonferroni corrections were performed. For (**H**–**K**) analysis was taken over 5 repeats of 1 µs, the first 100 ns discarded as equilibration.

TMEM263 is a small (12 kDa), vertebrate-specific protein with a poorly characterised structure and no obvious functional domains. TMHMM predicts TMEM263 consists of 2 helical transmembrane domains (TM), formed by amino acid residues 38–60 and 80–102, which are well conserved across vertebrate species (Fig. 3A and Supplementary Fig. 4A and refs. ^26,27^). Overexpression of protein truncations of EGFP-TMEM263 revealed that both putative TM domains are required for proper localisation of TMEM263 to the ER and only constructs including both putative TM domains were shown to induce lipid droplet accumulation upon overexpression (Fig. 3B–D and Supplementary Fig. 4B). Specifically, deletion of the C-terminal, 8 amino acid tail (EGFP-TMEM263^1−108^), which contains the COPI-binding motif, led to a significant reduction in the percentage of cells with EGFP-TMEM263-positive lipid droplets (from ~ 80% to ~ 40%) (Fig. 3B–D). Of note, EGFP-TMEM263^1−108^ and EGFP-TMEM263^33−108^ constructs expressed at lower levels, which may account for some difference in lipid droplet accumulation (Supplementary Fig. 4B). Deletion of the N-terminal 33 amino acids, however had no impact on EGFP-TMEM263^33−116^ localisation to the ER (Fig. 3B–D), its ability to induce EGFP-TMEM263-positive lipid droplet accumulation or to promote lipid droplet clustering (Supplementary Fig. 4C–E). Consistently, the TMEM263 mutation associated with dwarfism in chickens, W59*^27^, which introduces an early stop codon and therefore results in the loss of TM2, shows a cytoplasmic distribution and fails to localise to the ER or induce lipid droplet accumulation (Fig. 3E, F and Supplementary Fig. 4F).

The current TMEM263 AlphaFold2 (AF2) model (AF-Q8WUH6-F1) hosted on the EBI database (https://alphafold.ebi.ac.uk/entry/Q8WUH6) suggests that it exists in an almost entirely unstructured conformation. Using ColabFold, we generated 40 AF2 predictions of TMEM263 with the first 33 residues removed (TMEM263^33−116^), since we found this region to be functionally redundant (Supplementary Fig. 4B–E). Our data reveal that the top 5 ranked models are structurally similar, with TMEM263 folding into a TM hairpin structure (Fig. 3G).

To interrogate the structure further, we modelled our AF2 model in a membrane comprising short-tail lipids to mimic the ER bilayer (DMPC and DLPC) and neutral lipids (TAG, DAG, cholesterol) and performed five atomistic molecular dynamics (MD) simulations each lasting 1 μs. Our data reveal a high degree of dynamics within the TMEM263 fold (Supplementary Fig. 4G). The core structure of TMEM263 is preserved, with each of the two TMs stabilised by flanking basic residues at either end: R37 and K47 for TM1, and K84 and K104 for TM2 (Fig. 3H). This arrangement results in a very short TM segment of 10 residues (35–46; TM1), a relatively long linker comprising both helix (49–68) and loop (69–79), and a more typical TM segment of 23 residues (80–102; TM2) (Fig. 3I and Supplementary Fig. 4H). This trapezoid-like structure remains relatively stable throughout the MD simulations, with dynamic but consistent distances between the tops and bottoms of TM1 and TM2 (Supplementary Fig. 4I), as well as stable TM orientations (Supplementary Fig. 4J). Furthermore, the four basic residues remain consistently associated with their respective lipid headgroups (Fig. 3J), while the TMEM263 loop remains solvated – and therefore does not partition fully into the membrane – throughout the simulations (Fig. 3K). Together, these findings support the presence of two stable helical TM domains that fully span the membrane.

**Fig. 4:**
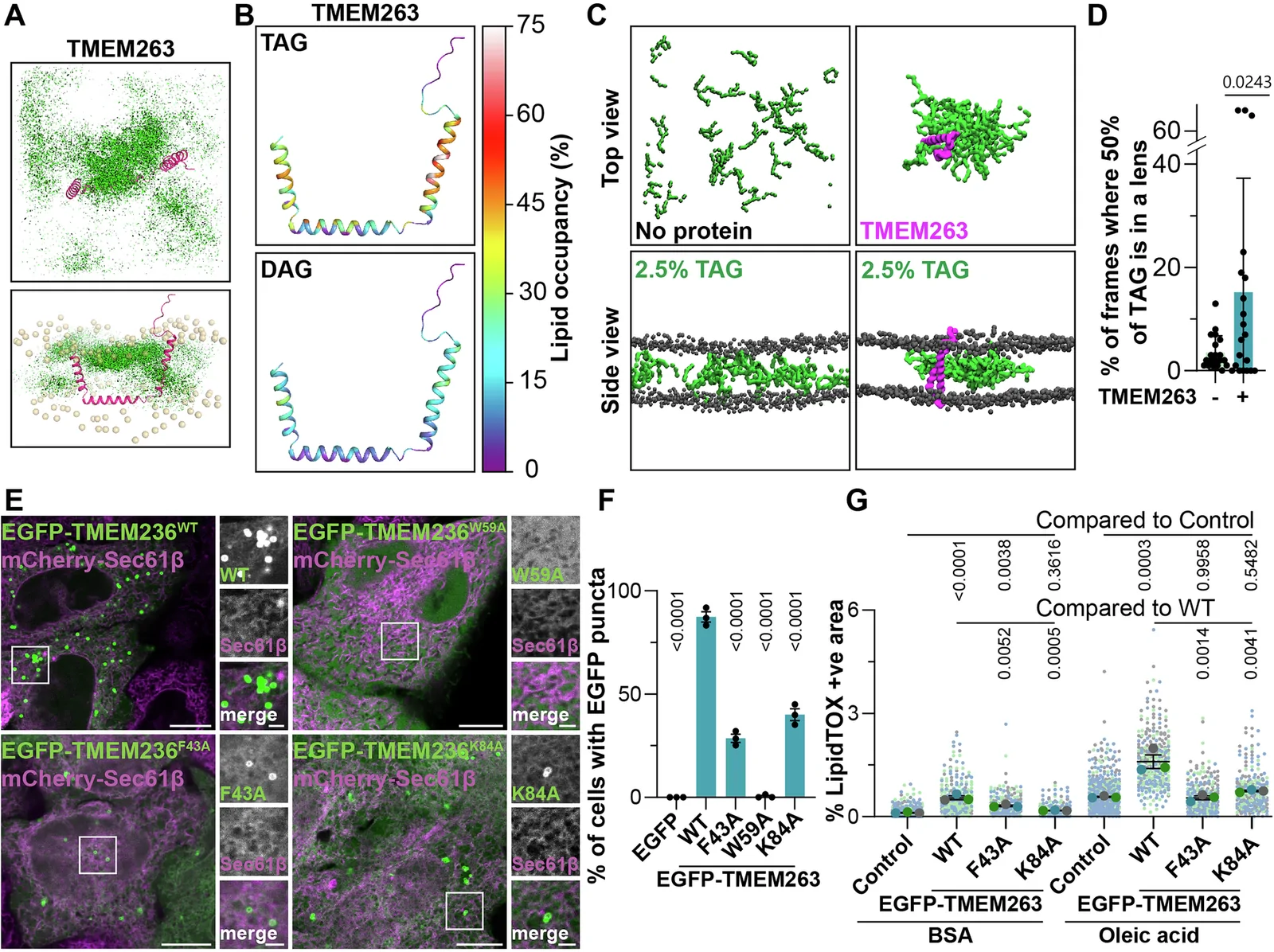
TMEM263 can bind and concentrate lipids in a phospholipid bilayer. **A** Spatial distribution of neutral lipids around TMEM263 during atomistic MD simulation. The position of neutral lipids (green) sampled across the MD trajectory is shown relative to a single representative TMEM263 structure (pink cartoon). Phosphate headgroups are depicted as orange spheres. The dense central accumulation reflects regions of preferential neutral lipid localisation around TMEM263 over the course of the simulation. **B** PyLipID-derived TAG and DAG binding occupancies based on atomistic MD analysis. Occupancies were calculated for each residue as % of frames where the residue is within 0.35 nm of TAG/DAG. Data are projected as a coloured heatmap on a representative structure. **C** Representative frames of coarse-grained simulation of a bilayer containing 2.5% TAG with or without TMEM263^33−116^ present. **D** Quantification of (**C**) measuring % of frames where at least 50% of TAG is present in the largest lens (cluster), with contacts within clusters defined as within 0.55 nm, *n* = 20 per condition. A two-tailed unpaired Student’s *t* test was performed, and error bars represent standard deviation. **E–G** Lipid binding and key structural residues are functionally important for TMEM263. HeLa cells stably expressing mCherry-Sec61β (ER) were transfected as indicated and imaged live (**E**). HeLa cells were transfected as indicated, fixed and scored for the presence of EGFP-positive puncta (**F**). *n* = 3, Cochran-Mantel-Haenszel chi-squared tests with Haldane-Anscombe and Bonferroni corrections were performed. HeLa cells were transfected as indicated and incubated for 16 h with BSA or 30 µM oleic acid, fixed and stained with LipidTOX. The % LipidTOX positive area per cell was quantified (**G**). Control represents untransfected cells in the same field of view as the EGFP-TMEM263-expressing cell. *n* = 3, ordinary one-way ANOVA with Dunnett’s multiple comparisons tests were performed. Data from control and EGFP-TMEM263^WT^-expressing cells are also included in Fig. 2C. Scale bars are 10 µm and 2 μm for zoomed images, bars extend to the mean, colour-coded individual data points correspond to individual cells from different experiments (**G**), lines show mean values, and error bars represent SEM, unless stated otherwise.

As our cell work indicates TMEM263 is ER-resident and EGFP-tagged TMEM263 localises to nascent lipid droplets, we used a series of simulations to study the relationship of TMEM263 and neutral lipids in a bilayer. Firstly, we observed from our atomistic MD data that neutral lipids tend to accumulate in the middle of TMEM263, between the two TM domains (Fig. 4A). This led us to hypothesise that specific residue(s) in TMEM263 may bind directly to lipids. To provide a higher resolution view of this, we carried out atomistic simulations of TMEM263 in a bilayer containing TAG and DAG, and used the PyLipID package to assess the neutral lipid binding properties of individual residues^38^. This analysis shows there is a clear preference for TAG and DAG binding to the transmembrane domains of TMEM263 (Fig. 4B and Supplementary Fig. 5A). Of particular note are F43, which has the highest neutral lipid binding on TM1, and the stretch of residues from 89–95 (AVAGGVT) on TM2 which supports a very high amount of neutral lipid binding (Supplementary Fig. 5A). Interestingly, the hydrophobic TM domains of seipin/BSCL2, a key lipid droplet biogenesis regulator^10^, have been shown in molecular dynamics simulations to trap and cluster TAG and DAG in a bilayer, concentrating the lipids and lowering the energy barrier to lens formation^21^.

To better probe the longer timescale dynamics of neutral lipid binding to TMEM263, we used coarse-grained simulations with the Martini 2.2 force field. To model how TMEM263 behaves during lipid lens formation, we introduced neutral lipids, 6% each of TAG, DAG, and cholesterol esters in a bilayer and nine copies of TMEM263^33−116^ (i.e., one copy per region of the 3 × 3 grid) into the simulation (Supplementary Fig. 5B). At these high concentrations, the neutral lipids naturally coalesced from their original sites, laid down in a 3 × 3 grid, into a ‘lens’ over the course of the 7 μs simulation (Supplementary Fig. 5B)^15–17^. In all 9 repeats, TMEM263 was consistently recruited to the lens border, in the same orientation, where it remained throughout the simulation. Furthermore, TMEM263 remained evenly spaced around the lens border, and there was no evidence of protein-protein interactions or protein oligomerisation (Supplementary Fig. 5B). Consistently, immunoprecipitation with differentially tagged TMEM263 proteins showed no evidence of TMEM263 being able to bind to itself (Supplementary Fig. 5C).

To test whether TMEM263 may influence lipid lens formation, coarse-grained simulations were repeated using a lower concentration of 2.5% TAG in the bilayer, and no other neutral lipids (Fig. 4C, D). This concentration is below the threshold at which lipids would coalesce spontaneously^14–17^, as seen from our no protein control (Fig. 4C, left column) and thus allowed us to test whether TMEM263 could contribute to lens formation. The data showed that one molecule of TMEM263 was sufficient to induce a significant increase in the proportion of TAG within the lens, supporting the hypothesis that TMEM263 may promote lipid droplet biogenesis. These data are consistent with previous reports that hairpin proteins and TM domains have affinity for TAG and can localise around a neutral lipid lens in a bilayer^39^.

To experimentally test some of the above observations from the atomistic simulations and PyLipID analysis, we mutated residues in TMEM263. We introduced F43A to perturb the observed neutral lipid binding to TM1 (Fig. 4B and Supplementary Fig. 5A), W59A to perturb the bulky residue at the start of the inter-TM linker (Fig. 3H), and K84A to destabilise the interaction of the bottom of TM2 with the lipid headgroup (Fig. 3H–J). The overexpression of these three TMEM263 mutations in HeLa cells resulted in a significantly reduced percentage of cells expressing EGFP-TMEM263 positive puncta compared to wild-type control (Fig. 4E, F and Supplementary Fig. 5D). Co-staining with the ER marker, mCherry-Sec61β confirmed that F43A and K84A were still correctly localised to ER, while W59A showed a cytoplasmic distribution (Fig. 4E). As it is not localised properly, it would not be possible to interrogate the importance of the W59 residue, independent of ER localisation and therefore the W59A mutant was omitted from subsequent analysis. Expression of F43A and K84A mutants failed to promote an increase in the LipidTOX-positive area above the untransfected controls in either BSA or OA-fed conditions, as observed with the expression of wild-type EGFP-TMEM263 (Fig. 4G and Supplementary Fig. 5D, E). Furthermore, neither the F43A nor the K84A mutant induced a change in the distribution of LipidTOX-positive droplets from dispersed to clustered as seen upon expression of wild-type EGFP-TMEM263 (Supplementary Fig. 5D–F).

**Fig. 5:**
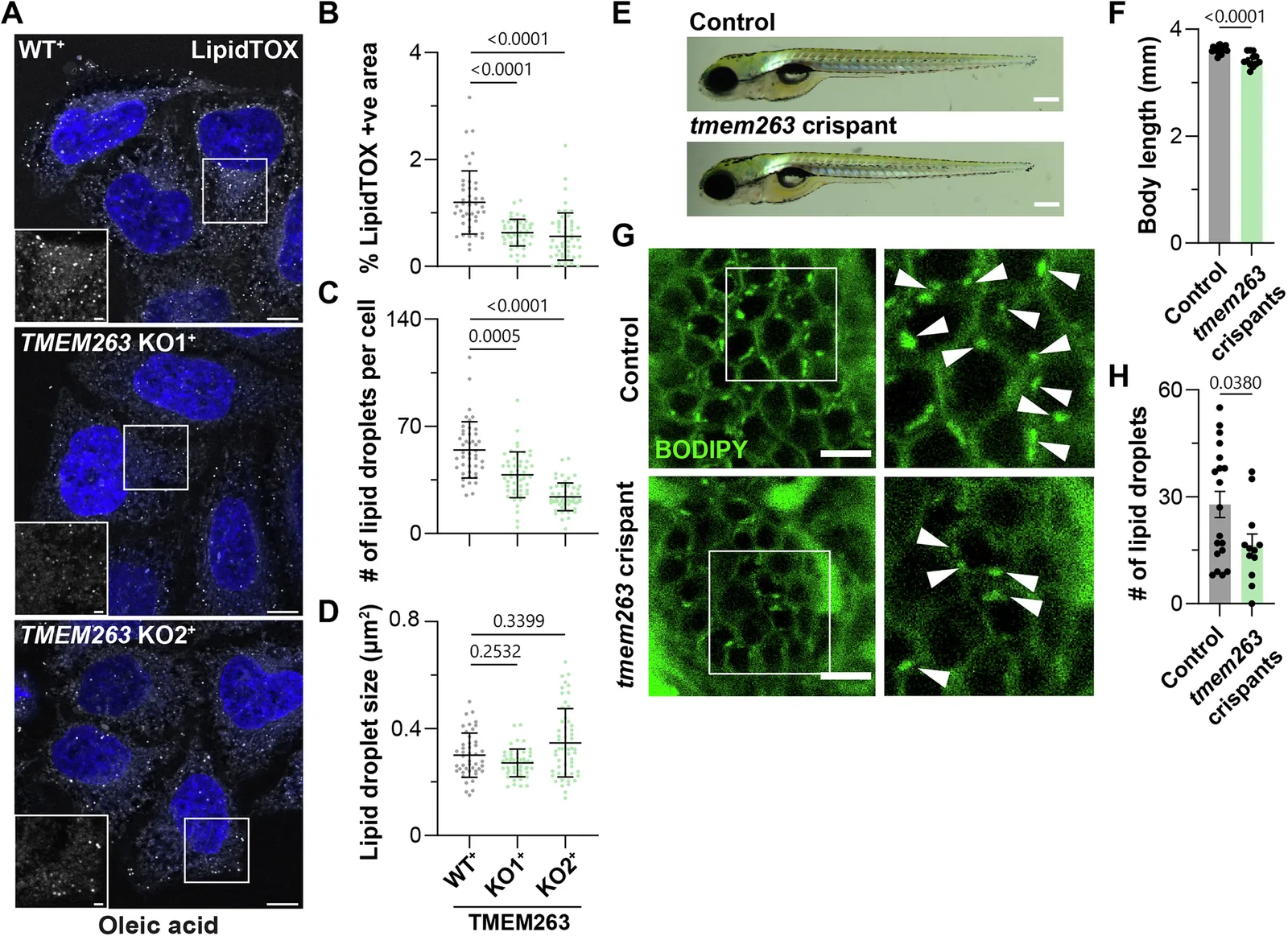
TMEM263 is required for efficient lipid droplet formation in vivo. **A–D** TMEM263 knock-out impairs lipid droplet formation. To remove residual TMEM263 from CRISPR KO HeLa, cells were treated with TMEM263 shRNA and subsequently referred to as TMEM263 knock-out 1 (KO1^+^) and TMEM263 KO2^+^. Parental cells were treated with control shRNA and referred to as WT^+^. HeLa cells were treated for 16 h with 30 µM oleic acid and stained with LipidTOX (**A**). Scale bars are 10 µm and 2 μm for zoomed images. The % LipidTOX-positive area per cell (**B**), the number of lipid droplets per cell (**C**), and the size of lipid droplets per cell was quantified (**D**). For (**B**–**D**) graphs show representative data from one repeat, *n* = 4, with at least 45 cells per condition. Individual data points correspond to individual cells. Kruskal-Wallis tests with Dunn’s multiple comparisons tests were performed. All 4 biological replicates had similar results. **E–H** TMEM263 mutation in zebrafish impairs lipid droplet formation. **E** Representative images of wild-type control versus *tmem263* crispant zebrafish at 5 days post fertilisation (dpf). Scale bars are 250 μm. **F** Quantification of body length of control and *tmem263* crispant fish at 6 dpf. *n* = 19 for control and *n* = 12 for *tmem263* crispants. A two-tailed unpaired Student’s *t* test was performed. **G** Zebrafish (6 dpf) were fed 300 µM oleic acid for 24 h, followed by incubation with BODIPY for 2 h. Representative confocal microscope z-slices from the intestine. Scale bars are 10 μm. White arrowheads in insets show lipid droplets. **H** Quantification of lipid droplet number per image from control and *tmem263* crispant fish. *n* = 19 for control and *n* = 12 for *tmem263* crispants. A two-tailed unpaired Student’s *t* test was performed. Lines show mean values, bars extend to the mean and error bars represent SD.

Together, these data suggest that TMEM263 can bind neutral lipids in the ER bilayer, potentially to aid lens formation to support lipid droplet biogenesis. Based on these data, we hypothesised that loss or mutation of TMEM263 would impair lipid droplet formation. To interrogate this hypothesis, we generated CRISPR/Cas9 knock-out (KO) HeLa cell lines (Supplementary Fig. 6A, B) and CRISPR/Cas9 mutant zebrafish lines (Supplementary Fig. 6C, D). Two clonal *TMEM263* KO HeLa cell lines showed significantly lower BODIPY-positive area compared to parental controls following OA feeding (Supplementary Fig. 6E, F). Due to high levels of variation in lipid droplet size and number in the KO clones, probably as a result of residual TMEM263 protein expression, we treated the cells with shRNA to silence any remaining transcript. Using these cells, referred to as KO1/2^+^, we observed that loss of *TMEM263* led to reduced percentage LipidTOX-positive area as well as reduced lipid droplet number compared to controls in OA-fed conditions (Fig. 5A–C). Loss of *TMEM263* has no effect on lipid droplet size (Fig. 5D).

The majority of *tmem263* crispant fish (77.5%) showed abnormal phenotypes, including oedema, bent tails and tail truncations (Supplementary Fig. 6G, H), and failed to survive past 5 days post fertilisation (dpf). The surviving 22.5% *tmem263* crispant fish showed a small but significant decrease in body length (Fig. 5E, F), consistent with an established role for TMEM263 in organismal growth^26–28^. Lipid droplet accumulation in the intestine was significantly decreased in *tmem263* crispants following OA feeding (Fig. 5G, H). It is important to note that given that experiments could only be carried out in 22.5% of *tmem263* crispants with less severe phenotypes, it is likely the results presented here are under-representing the extent of the growth and lipid droplet phenotype. Altogether, these data demonstrate TMEM263 is necessary for efficient lipid droplet formation in vivo. These results provide a previously uncharacterised link between lipid droplet homeostasis and organismal growth.

## Discussion

Using in silico*, in cellulo* and in vivo models, we have identified TMEM263 as an ER-resident protein and a COPI cargo which contributes to efficient lipid droplet formation. There is a close association between ER homeostasis, COPI and lipid droplet biology. Indeed, the COPI/Arf1 machinery has been identified both as a direct regulator of lipid droplet dynamics^40,41^, and an indirect via regulation of ER homeostasis. Here, we describe TMEM263 as another molecular link between COPI-ER-lipid droplets.

Our data demonstrate that TMEM263 overexpression causes the accumulation of enlarged lipid droplets that have a propensity to cluster, particularly upon lipid feeding, and are enriched with classical lipid droplet proteins, including perilipin 2 and LPCAT1/2. Mechanistically, TMEM263 functions early in lipid droplet biogenesis, labelling nascent sites in the ER before they acquire HPos or neutral lipid staining. These data indicate TMEM263 is recruited to the very early stages of lipid droplet biogenesis in the ER. There is however, no evidence it colocalises with the key biogenesis regulator, seipin. Furthermore, loss of TMEM263 does not phenocopy seipin knock-out in cells i.e., there is no evidence of the characteristic supersized lipid droplets^42,43^, nor in vivo^44^, and while seipin is evolutionarily conserved, the later emergence of vertebrate-specific TMEM263^45^ suggests they have not co-evolved. Therefore, it remains an interesting open question how TMEM263 coordinates with the established mechanisms to control the onset of lipid droplet formation. Instead, our work demonstrates TMEM263 has an affinity for neutral lipids, which may underpin its role in regulating lipid droplets. Specifically, TMEM263 contains two helical transmembrane domains, which are both required to anchor it to the ER. Molecular dynamics simulations show these TM domains have affinity for neutral lipids and promote their concentration in a phospholipid bilayer. These data indicate TMEM263 may contribute to lipid droplet biogenesis by supporting lipid lens formation. We showed that mutation of the putative lipid-binding residue (F43A) and key structural residues (W59A, K84A) all impair EGFP-TMEM263-induced lipid droplet formation, while W59A resulted in complete mislocalisation. Further studies of the protein-protein interactions of TMEM263 would be key in elucidating whether this neutral lipid affinity is the primary role of TMEM263 or whether it has additional roles. For example, neutral lipid binding may simultaneously aid lens formation and support recruitment of TMEM263 to early lenses, where it could serve a secondary purpose, such as enzyme recruitment, retention, or activity.

Interestingly, our data also demonstrate that both overexpressed and endogenous TMEM263 can localise in ‘rings’ around the LipidTOX core of lipid droplets. Here, EGFP-TMEM263 co-localises with PLIN2, which associates with lipid droplets via an amphipathic helix^10,46^. As a transmembrane protein, it is unlikely that TMEM263 could transition, via the so-called ER-to-lipid droplet (ERTOLD) route, to lipid droplet monolayers. One hypothesis is that these ‘rings’ are the result of tethering of TMEM263-enriched ER around the mature lipid droplet, similar to the association of SNX14 with lipid droplets^47^. This would be consistent with the clustering phenotype we observe upon expression of EGFP-TMEM263, as this has been observed during increased ER-LD connectivity^48^. However, it must be noted that TMEM263 was also identified in isolated lipid droplets treated with ionic chaotropes, to specifically preserve monolayer-integrated proteins as opposed to peripherally associated proteins^36^. Additionally, the ability of TMEM263 to associate with mature lipid droplets is further supported in the literature by its identification in proximity labelling of the lipid droplet surface in U2OS cells^12^. To understand the association between mature lipid droplets and TMEM263, additional investigation, similar to the elegant studies carried out by Dhiman et al.^49^ for UBXD8/FAF2, are required.

While there are still questions about the molecular regulation of TMEM263, the loss or mutation of TMEM263, both in cells and in vivo, in zebrafish larvae, leads to a significant reduction in the formation of lipid droplets, highlighting its importance. The biggest mystery remains understanding how loss or mutation of TMEM263, an ER-resident protein and lipid droplet regulator, causes a severe impairment of organismal growth and bone health. The late emergence of TMEM263, which is vertebrate-specific, may reflect the increased regulatory complexity required in vertebrates to control lipid storage and metabolism. Human GWAS studies have shown a significant association between TMEM263 variants and bone mineral density, bone fracture risk, and osteoblast function^24,25^. Introduction of early stop codons in TMEM263 causes autosomal dwarfism in chickens^27^ and was linked to a case of severe fatal skeletal dysplasia and shortened limbs in a human foetus^28^. Data presented in our study suggest that in both cases, these stop codons would result in loss of the TMEM263 TM2 domain and functionally inactivate the protein. Consistently, TMEM263 KO mice showed proportional dwarfism, and were 30-40% smaller than wild-type littermates at 8 weeks old^26^. While the physiological consequences of TMEM263 loss are consistent across model species, there is no clear causative mechanism. In contrast to the observations in humans and chickens for example, the KO mice were born at the same weight and size as wildtypes, with growth defects appearing after 21 days, a period of intense long bone growth^26^. Furthermore, while the TMEM263 mutant chickens show no changes in plasma IGF1 levels^34,35^, the TMEM263 KO mice showed reduced circulating IGF1 levels, possibly a result of reduced GHR expression and reduced JAK2/STAT5 signalling in the liver^26^. There was no mention of lipid defects or lipodystrophy in any of these reports, however transcriptomics from the TMEM263 KO mouse livers identified fatty acid metabolism and steroid hydroxylase activity were among the most differentially expressed genes (GO terms), indicating that lipid handling and metabolism is grossly altered in these animals^26^. More extensive analysis of the model organisms, the KO mice and the zebrafish model we have generated here to explore lipid homeostasis is required to determine if there are changes in physiological resilience in response to lipid or metabolic challenges. Our data identified a clear cell-autonomous role for TMEM263 in the regulation of lipid droplets, and evidence in mouse and human tissue suggests TMEM263 is ubiquitously expressed in all tissues^26^. It therefore remains to be seen whether the whole-body defects in growth are a result of cell or tissue-specific roles.

The ability to sense and properly respond to changes in nutrient availability is fundamental to regulate growth. While we have a good understanding of how growth factors and amino acids integrate to promote growth pathways, such as mTORC1 and the autophagy-lysosome pathway, much less is known about the links between lipid homeostasis and growth. Dysregulation of lipid droplets and ER-lipid droplet crosstalk is widely linked to metabolic diseases such as obesity, non-alcoholic fatty liver disease (e.g., PNPLA3, PLIN3), and lipodystrophy (e.g., seipin/BSCL2, AGPAT2)^11,42^. Our study establishes a direct link between lipid droplets and the regulation of whole-body growth and bone health. Previous studies have identified lytic bone lesions in patients with AGPAT mutations^51^. Furthermore, mutations in the lipophagy regulator, spartin, are associated with short stature^52^ and possibly GH deficiency^53^ and mutations in CAV1 or PTRF, which result in congenital generalised lipodystrophy, have been associated with short stature and skeletal abnormalities^54–56^. In all cases, however, the mechanisms are poorly understood, but intriguingly, a study by Rendina-Ruedy et al.^50^ revealed lipid droplets play an important role during osteoblast differentiation. In TMEM263, we have ultimately identified a fundamental mechanistic link between lipid homeostasis and organismal growth.

## Methods

### Cell lines and treatments

Human cervical cancer HeLa cells, HEK293T cells, wild-type mouse embryonic fibroblasts (MEF), and RPE1-hTERT cells were cultured in DMEM (D6546, Sigma-Aldrich) supplemented with 2 mM glutamine, 10% FBS and 100 U/ml penicillin–streptomycin at 37 °C, 5% CO_2_. All cells lines were obtained from collaborators, except from HEK293T cells that were purchased by ATCC. Where indicated, 24 h post-transfection, cells were incubated for 16 h in complete DMEM supplemented with 30 µM oleic acid (O1383, Sigma-Aldrich) in 10% fatty acid-free BSA (A8806, Sigma-Aldrich) or 10% fatty acid-free BSA only as vehicle control. Sodium oleate was prepared by slowly adding 400 μL of oleic acid (O1383, Sigma-Aldrich) into 30 mL of pre-warmed (~40 °C) ultrapure water with 400 μL of 5 M NaOH, while constantly stirring until all micelles were dissolved, resulting in a stock concentration of 41 mM. Additional NaOH was added as necessary to dissolve all micelles. Sodium oleate was diluted in warm complete DMEM to a final concentration of 200 μM and added to cells to observe lipid droplet formation using live imaging.

### Generation of TMEM263 KO cell lines

gRNA targeting *TMEM263* was cloned into pSpCas9(BB)-2A-Puro using Bbs1-HF. gRNA 1: GGUAUCUUCAGUGUUACAAA (to generate *TMEM263* KO1) and 2: CUUAAUGAUGAACCACCAGA (to generate *TMEM263* KO2). HeLa cells were transfected with FuGENE HD (Promega) overnight and selected with 3 μg/ml puromycin for 48 h. To generate single clones, cells were subsequently subject to serial dilution. Clones were screened by western blot. shRNA targeting against the UTR of TMEM263 was purchased by Sigma-Aldrich (MISSION, #TRCN0000148824), and non-targeting shRNA was used as a control (addgene #8453). Cells were selected using puromycin (3 μg/ml).

### DNA and viral production

mCherry-Sec61β (addgene #49155) was subcloned into pXLG3 (SpeI and KpnI). pLVXpuro- εCOPI-GFP was a gift from Prof David Stephens, University of Bristol (addgene #66604). pEFIRES-P-mCherry-Hpos was purchased from addgene (#87159). TMEM263 cDNA (NP_001306590.1) was purchased from Integrated DNA Technologies (IDT) and cloned into pEGFP-C2 (Clontech), p-mCherry2-C1 (addgene #54563)), FLAG-HA-pcDNA3.1 (addgene #52535). Primers used to generate TMEM263 truncations and point mutations in pEGFP-C2, are shown in Supplementary Table 1. Point mutants were generated using the QuikChange XL site-directed mutagenesis kit (Agilent 200517). All transient transfections were performed using FuGENE6 transfection reagent (Promega E2691) or Lipofectamine2000 according to manufacturer instructions. Transient transfection of HEK293T cells was carried out using 10 μg/μl polyethylenimine (PEI) with a 3:1 ratio PEI:DNA.

For virus production, HEK293T cells were transfected with a plasmid containing the gene of interest and packaging vectors pAX2 and pMDG2 for 72 h using 10 μg/μl PEI with a 3:1 ratio PEI:DNA. Viruses were harvested, filtered and incubated on cells overnight.

### Zebrafish husbandry and CRISPR-mutant lines

Zebrafish (*Danio rerio*) were raised and maintained under standard conditions^57^. Experiments were approved by the local ethics committee (the Animal Welfare and Ethical Review Body of the University of Bristol) and performed under a UK Home Office project licence.

Crispant *tmem263* zebrafish lines were generated using CRISPR-Cas9 mutagenesis, as previously performed^58^. Briefly, gRNAs targeting exon 3 of *tmem263* (ENSDARG00000030465) were synthesised (2 nmol, Alt-R™ CRISPR Cas9 crRNA, IDT): CRISPR-guide 1 – ATGGGAGTAGGCCTGGTGAAGGG; CRISPR-guide 2 – GACCATCCCCAGCCTCAGCCTGG, CRISPR-guide 3 – GGCGCTGTTGGGGCTACAGTGGG. crRNAs were each annealed to Alt-R CRISPR-Cas9 tracrRNA (5nmol, IDT) at 95 °C for 5 min to form a 57 mM gRNA. Each gRNA was then incubated with Alt-RTM S.p. Cas9 Nuclease V3 (diluted to 57 mM, IDT) for 5 min at 37 °C to assemble the ribonucleoprotein (RNP) for injection. The yolk of 1-cell stage zebrafish embryos was injected with 1 nl of RNP (1000 pg gRNA and 4700 pg of Cas9), and surviving embryos were raised under normal husbandry conditions. All three gRNAs were injected in combination.

### DNA extraction and genotyping of zebrafish lines

For DNA extraction, whole embryos were incubated in base solution (25 mM NaOH, 0.2 mM EDTA) for 30 min at 95 ^o^C before addition of an equal volume of neutralisation solution (40 mM Tris–HCl, pH 5.0). For genotyping, DNA was amplified using the following primers: F- TGCAACCTGCATCTTATGGAGA, R- AGAGGTCCGTGTCTCTACCC. Amplicons were sent for Sanger sequencing.

### Lipid droplet analysis in zebrafish

BODIPY FL C_5_ (D3834, Invitrogen) was used for live imaging of lipid droplets in the intestines of larvae following OA treatment. Briefly, larvae were fed 300 μM oleic acid in Danieau’s buffer for 24 h from 5-6 dpf, rinsed three times, then placed in 3123 μM BODIPY in Danieau’s buffer for 2 h and imaged.

### Immunofluorescence

Cells were seeded onto glass coverslips for 24–48 h then fixed using 4% paraformaldehyde/PBS for 10 min and permeabilised in 0.2% Triton X-100/PBS for 8 min, all at room temperature. Alternatively, 0.1% saponin/PBS was used as permeabilisation buffer when staining for endogenous TMEM263 and seipin, and 0.01% saponin added to all subsequent incubation and wash solutions. Coverslips were incubated with blocking buffer (5% BSA/PBS-0.05% Tween) for 1 h at room temperature. Coverslips were incubated in primary antibodies in blocking solution overnight at 4 °C. Primary antibodies used in this study: γCOP (Proteintech, 12393-1-AP, rabbit, 1:1000), ADRP/Perilipin-2 (PLIN2, Proteintech, 15294-1-AP, rabbit 1:400), TMEM263 (Invitrogen, PA5-70960, rabbit 1:200 & NovusBio, BP2-83690, rabbit, 1:200), Flag M2 (Sigma-Aldrich, F1804, mouse, 1:1000), Sec61β (Cell Signalling Technology, #14648, rabbit, 1:200), TGN46 (GeneTex, GTX74290, sheep, 1:2000), BSCL2/Seipin (Abnova, H00026580-A02, mouse, 1:200), LPCAT1 (Abcam, ab214034, rabbit, 1:250), LPCAT2 (Abcam, ab224244, rabbit, 1:250). Coverslips were washed and incubated in the appropriate Alexa Fluor-conjugated secondary antibodies (Thermo Fisher Scientific, 1:1000) in blocking buffer for 1 h at room temperature. The following secondary antibodies were used: goat anti-rabbit 594 (A11037, Invitrogen, Thermo Fisher Scientific), donkey anti-rabbit 568 (A10042, Invitrogen, Thermo Fisher Scientific), goat anti-rabbit 488 (A11034, Invitrogen, Thermo Fisher Scientific), donkey anti-mouse 568 (A10037, Invitrogen, Thermo Fisher Scientific), and donkey anti-sheep 633 (A21100, Invitrogen, Thermo Fisher Scientific). Where indicated, cells were incubated at room temperature with HCS LipidTOX™ Deep Red Neutral Lipid Stain (H34477, Invitrogen 1:1000) for 30 min or BODIPY-493/503 (25892, Cayman chemical 1 µg/ml) for 10 min, diluted in PBS. Coverslips were mounted using ProLong™ Gold Antifade Mountant with DAPI (Invitrogen, Thermo Fisher Scientific, P36934). For the STED imaging, goat anti-rabbit STAR RED (abberior, STRED-1002, 1:200) and goat anti-ms STAR ORANGE (abberior, STORANG-1001, 1:200) were used as secondaries, and the samples were mounted with Mount Solid Antifade (abberior, MM-2013)

### Microscopy

Cells were imaged using either a Leica SP8 AOBS confocal laser scanning microscope with 63 × HC PL APO CS2 oil objective, or an Olympus IXplore spinning disk system based on Yokogawa CSU-W1 SoRa with 60X NA 1.4 oil objective.

For stimulated emission depletion (STED) microscopy, the MIRAVA POLYSCOPE (abberior instruments GmbH) with 60x NA 1.42 oil objective with excitation lasers at 640 nm, 561 nm and 488 nm was used.

For live imaging of lipid droplet formation, cells were seeded onto glass-bottom 24 well-plates and 24–48 h later were transfected with EGFP-TMEM263 and mCherry-HPos. 24 h after transfection, cells were stained with LipidTOX for 1 h and then imaged with Olympus IXplore spinning disk system based on Yokogawa CSU-W1 SoRa with a 60X oil NA 1.5 objective with temperature and CO_2_ control. Oleic acid in the form of sodium oleate (200 μM) was added after cell selection, and a time lapse was acquired every 10 s for 18 min.

For live imaging, fish were anaesthetised using 0.1 mg/ml MS222 (Tricaine methane sulfonate) diluted in Danieau’s buffer and imaged laterally. Images of live larvae at 5 dpf were obtained using a Leica MZ10 F modular stereo microscope system at 3.2 x magnification. Alternatively, zebrafish larvae were mounted in 1% LMP agarose (16520050, Thermofisher) and imaged using a Leica SP5-II AOBS tandem scanner confocal microscope attached to a Leica DMI 6000 inverted epifluorescence microscope and oil immersion 20 x NA 0.7 objectives run using Leica LAS AF software (Leica, Germany).

### Image quantification

The percentage of cells containing EGFP-positive puncta was scored manually on a widefield Leica DMI6000 inverted epifluorescence microscope. Scoring of the number of TMEM263 puncta (Fig. 2I), scoring of the percentage of LipidTOX positive EGFP puncta (Supplementary Fig. 2B), and analysis of lipid droplet distribution (i.e., dispersed, intermediate or clustered) was carried out manually post-imaging. The appearance time of each signal (EGFP-TMEM263, mCherry-HPos, LipidTOX; Fig. 2K) was assessed manually from the acquired timelapse. For lipid droplet quantification (i.e., percentage area), a constant threshold was applied across all images, then an ROI was generated for each individual cell, and the ‘analyse particles’ tool in ImageJ/Fiji^59,60^ was utilised with no set minimum or maximum size and circularity 0.00-1.00. Τhe quantification of size and number of lipid droplets was done in MATLAB (MathWorks, v. 9.14, R2023a). The code from Olzmann lab^12^ (function ‘imfindcircles’) was used for the detection of lipid droplets from single images. When z-stacks were analysed, the 99.8th percentile intensity was used to cap extreme bright pixels and after ‘imfindcircles’, the mean intensity and the signal-to-background ratio using 3 pixels beyond the radius was calculated for each detected lipid droplet per slice. Then, spatially overlapping lipid droplets (centres within 5 px in XY) were removed, keeping the ones with higher intensity and higher signal-to-background ratio lipid droplets. Further filtering was done using two sets of thresholds for intensity and signal-to-background ratio, one to detect the very bright lipid droplets and one to detect the less bright but very distinct lipid droplets. Cell borders were drawn in MATLAB, and lipid droplets were analysed per cell. For the linescan analysis of EGFP/PLIN2/LipidTOX (Supplementary Fig. 3B), 30 lipid droplets from 1 independent experiment were analysed. A line was drawn across individual lipid droplets, and using the ‘Plot Profile’ function of ImageJ/Fiji the raw fluorescent intensities of the 3 channels were determined. The fluorescent intensities were then normalised to the maximum fluorescent intensity and the longest distance of each channel and lipid droplet. For the co-localisation analysis (Supplementary Fig. 1F), the background was subtracted using a rolling ball radius, a threshold was applied, and then the plugin Coloc 2 of Fiji was used, and the Manders coefficient (M2) was extracted. For the detection of endogenous puncta of TMEM263 and Seipin (Supplementary Fig. 3M), a custom workflow for the ModularImageAnalysis (MIA) ImageJ plugin was used^61^. First, lipid droplets were detected in z-stack confocal images, which were subject to a 2D Gaussian filter to reduce noise (sigma = 2 px), followed by background subtraction (rolling ball radius = 20 px). These images were binarised using the Otsu method^62^ and proximal lipid droplets, which had become merged into a single contiguous binary region, separated using a distance-based watershed transform^63^. Individual droplets were detected in 3D from this optimised binary image stack using connected components labelling and subject to a minimum 2D droplet area (projected into the XY plan) of 0.04 μm². TMEM263 detection was performed on background-subtracted (rolling ball radius = 50 px) images using TrackMate’s Laplacian of Gaussian spot detector^64^. All TMEM263 puncta within 0.22 μm of a lipid droplet surface were linked to that droplet, and any puncta within this distance of multiple droplets was linked to the closest lipid droplet. The same approach was applied to the detection and linking of seipin puncta. Nearest neighbour analysis was performed between TMEM263 and seipin puncta that were linked to the same lipid droplet. Then, using MATLAB, the distance from the nearest neighbour analysis was used to define the categories of colocalisation between TMEM263 and Seipin (distance ≤ 0.22 μm), adjacent (0.22 μm < distance ≤ 0.44 μm), and distinct (distance > 0.44 μm).

For zebrafish, standard larval lengths were obtained from lateral images at 5 dpf by measuring from the nose of the fish to the end of the tail manually in Fiji. For lipid droplet analysis, a 68 μm^2^ region of interest (ROI) was selected from one z-slice per larvae and BODIPY puncta were counted. Maximum projection images were assembled using LAS AF Lite software (Leica) and Fiji (Schindelin et al., 2012) (Fig. 5G). This experiment was repeated independently with similar results three times.

### Immunoprecipitation

HEK293T cells or HeLa cells were seeded in 10 cm dishes and lysed for immunoprecipitation (IP) 24 h after transfection. Cells were washed in ice-cold PBS and lysed, on ice, in 500 μL IP buffer (40 mM Tris-HCL pH 7.5, 0.5% NP-40 (IGEPAL CA-630, #18896, Sigma-Aldrich), supplemented with 1 x Halt™ Protease and Phosphatase Inhibitor Cocktail, EDTA-free (Thermo Fisher Scientific, 78443, 1:100)). Lysates were cleared by centrifugation for 10 min at 17115 x *g* at 4 °C. A whole cell lysate sample was removed, and the remaining supernatant was incubated with 10 μL of equilibrated GFP-Trap agarose beads (GTA-20, ChromoTek) for 2 h at 4 °C with constant rotation, then washed and samples boiled at 95 °C for 5 min in 1 x NuPAGE LDS sample buffer (NP0008, Invitrogen, Thermo Fisher Scientific) supplemented with 5% 2-mercaptoethanol.

### Immunoblotting

Immunoblotting was performed as described previously^65,66^. Briefly, samples were separated via SDS-PAGE using 4–12% NuPAGE gels, (Thermo Fisher Scientific, NP0335BOX and NP0336BOX) as per company instructions. Gels were transferred to methanol-activated PVDF membrane by either standard wet transfer or semi-dry transfer methods. Membranes were blocked for 1 h at room temperature in 5% milk in 0.05% PBS-Tween. Primary antibodies were diluted in blocking buffer and incubated overnight at 4 °C. The following primary antibodies were used: HA.11 (Biolegend, #901573, mouse, 1:1000), GFP (Roche, 11814460001, mouse, 1:2000; Proteintech, PABG1, Rabbit, 1:2000), mCherry (Abcam, ab167453, rabbit, 1:2000), TMEM263 (Invitrogen, PA5-70960, rabbit 1:400), γCOP (Proteintech, # 12393-1-AP, rabbit, 1:1000), βCOP (gift from D. Stephens, University of Bristol, mouse, 1:500), α-tubulin (Sigma-Aldrich T9026, mouse, 1:1000), and GAPDH (Cell Signalling Technology, #97166, mouse, 1:2000). After 3 washes with PBS-T, the blots were incubated for 1 h at room temperature with the following secondary antibodies diluted in blocking buffer: goat anti-mouse 680 (Invitrogen, Thermo Fisher Scientific, A21057, 1:20000) and goat anti-rabbit 800 (Invitrogen, Thermo Fisher Scientific, SA535571, 1:20000). Blots were washed 3 times with PBS-T and once with PBS before being imaged with LI-COR Odyssey xL (LI-COR Biotechnology) and quantified using Image Studio Lite software (LI-COR Biotechnology).

### Proteomics

#### TMT Labelling and High pH reversed-phase chromatography

Samples from four biological replicates of HeLa cells transfected with GFP or εCOPI-GFP were immunoprecipitated using GFP-trap beads as described above. Immuno-isolated samples were reduced (10 mM TCEP, 55 °C for 1 h), alkylated (18.75 mM iodoacetamide, room temperature for 30 min) and then digested from the beads with trypsin (1.25 µg trypsin; 37 °C, overnight). The resulting peptides were then labelled with TMTpro sixteen-plex reagents according to the manufacturer’s protocol (Thermo Fisher Scientific, Loughborough, LE11 5RG, UK) and the labelled samples pooled and desalted using a SepPak cartridge according to the manufacturer’s instructions (Waters, Milford, Massachusetts, USA). Eluate from the SepPak cartridge was evaporated to dryness and resuspended in buffer A (20 mM ammonium hydroxide, pH 10) prior to fractionation by high pH reversed-phase chromatography using an Ultimate 3000 liquid chromatography system (Thermo Scientific). In brief, the sample was loaded onto an XBridge BEH C18 Column (130 Å, 3.5 µm, 2.1 mm × 150 mm, Waters, UK) in buffer A and peptides eluted with an increasing gradient of buffer B (20 mM Ammonium Hydroxide in acetonitrile, pH 10) from 0–95% over 60 min. The resulting fractions (concatenated into 6 in total) were evaporated to dryness and resuspended in 1% formic acid prior to analysis by nano-LC MSMS using an Orbitrap Fusion Tribrid mass spectrometer (Thermo Scientific).

#### Nano-LC mass spectrometry

High pH RP fractions were further fractionated using an Ultimate 3000 nano-LC system in line with an Orbitrap Fusion Tribrid mass spectrometer (Thermo Scientific). In brief, peptides in 1% (vol/vol) formic acid were injected onto an Acclaim PepMap C18 nano-trap column (Thermo Scientific). After washing with 0.5% (vol/vol) acetonitrile 0.1% (vol/vol) formic acid, peptides were resolved on a 250 mm × 75 μm Acclaim PepMap C18 reverse phase analytical column (Thermo Scientific) over a 150 min organic gradient with a flow rate of 300 nL min^−1^. Solvent A was 0.1% formic acid, and Solvent B was aqueous 80% acetonitrile in 0.1% formic acid. Peptides were ionised by nano-electrospray ionisation at 2.0 kV using a stainless-steel emitter with an internal diameter of 30 μm (Thermo Scientific) and a capillary temperature of 275 °C.

All spectra were acquired using an Orbitrap Fusion Tribrid mass spectrometer controlled by Xcalibur 2.1 software (Thermo Scientific) and operated in data-dependent acquisition mode using an SPS-MS3 workflow. FTMS1 spectra were collected at a resolution of 120000, with an automatic gain control (AGC) target of 200000 and a max injection time of 50 ms. Precursors were filtered with an intensity threshold of 5000, according to charge state (to include charge states 2–7) and with monoisotopic peak determination set to peptide. Previously interrogated precursors were excluded using a dynamic window (60 s ± 10 ppm). The MS2 precursors were isolated with a quadrupole isolation window of 1.2 m/z. ITMS2 spectra were collected with an AGC target of 10000, max injection time of 70 ms and CID collision energy of 35%.

For FTMS3 analysis, the Orbitrap was operated at 50000 resolution with an AGC target of 50000 and a max injection time of 105 ms. Precursors were fragmented by high-energy collision dissociation (HCD) at a normalised collision energy of 60% to ensure maximal TMT reporter ion yield. Synchronous Precursor Selection (SPS) was enabled to include up to 10 MS2 fragment ions in the FTMS3 scan.

#### Data Analysis

The raw data files were processed and quantified using Proteome Discoverer software v2.4 (Thermo Scientific) and searched against the UniProt Human database (downloaded January 2024: 82415 entries) using the SEQUEST HT algorithm. Peptide precursor mass tolerance was set at 10 ppm, and MS/MS tolerance was set at 0.6 Da. Search criteria included oxidation of methionine (+15.995 Da), acetylation of the protein N-terminus (+42.011 Da) and methionine loss plus acetylation of the protein N-terminus (− 89.03 Da) as variable modifications and carbamidomethylation of cysteine (+57.021 Da) and the addition of the TMT mass tag (+304.207 Da) to peptide N-termini and lysine as fixed modifications. Searches were performed with full tryptic digestion, and a maximum of 2 missed cleavages were allowed. The reverse database search option was enabled, and all data was filtered to satisfy a false discovery rate (FDR) of 5%. COPI cargo were defined as proteins with log_2_ fold change (FC) > 2.5 and *p*-value < 0.005 εCOPI-GFP / GFP (*n* = 4). Proteins were further filtered for those containing COPI-interacting motifs, either KxKxx or KKxx (at the C-terminus) or RxR (within the last 30 amino acids of the C-terminus), and associated with ‘growth’ in the MGI database^30,31^. The results are presented in Fig. 1A, Supplementary Fig. 1C and in Supplementary Data 1.

### Statistical analysis

For the statistical analysis of quantitative values, two kinds of parametric tests were performed on experimental data from at least three individual experiments. The two-tailed unpaired Student’s *t* test or two-tailed one-way ordinary ANOVA for multiple comparison test with Dunnett’s correction was performed for comparisons between two or more than two conditions, respectively. For individual experiments, the Shapiro-Wilk test was used to test the normality of the data. For non-normally distributed data, the two-tailed Kruskal-Wallis test with Dunn’s correction was performed for comparisons between more than two conditions. All these statistical tests were performed with GraphPad Prism v10.2.3. For the statistical analysis of qualitative data (clustering, EGFP puncta positive, number of endogenous TMEM263 puncta), the two-tailed chi-squared (chisq.test) with Bonferroni correction or the Fisher’s exact test (fisher.test) with Bonferroni correction was performed on individual experiments. The two-tailed Cochran-Mantel-Haenszel chi-squared test (mantelhaen.test) with Haldane-Anscombe and Bonferroni corrections was performed to combine all replicates and shown in graphs. For the comparison of the number of endogenous TMEM263 puncta per lipid droplet (Fig. 2I), the two-tailed chi-squared test with Bonferroni correction was performed in pooled data and shown in the graph. All these statistical tests were performed in R v4.4.2. For all statistical tests, significance level was defined at α = 0.05 (*p* < 0.05) and 95% confidence intervals were calculated where applicable. The number of biological replicates (n) in cell based experiments that was use for the statistics is included in the figure legend. The number of independent repeats with similar results is the same as the number of replicates unless stated otherwise. For chi-squared tests, this information is in the Source Data file. In dot plots, colour-coded individual data points correspond to both individual cells from different experiments and the mean values of each experiment.

### Multiple sequence alignment

MSA generated in ClustalW^67^ and represented in JalView^68^. The following TMEM263 sequences were used: *H. sapiens* isoform a (human): NP_001306590.1; *H. sapiens* iso b (human): NP_001306595.1; *P. macrocephalus* (sperm whale): XP_054941260.1; *D. rotundus* (common vampire bat): XP_045042869.1; *G. gallus* (chicken): NP_001006244.2; *L. disocolor* (Swift parrot): XP_065535720.1; *A. carolinensis* (green anole): XP_016849149.1; *C. mydas* (Green sea turtle): XP_037736208.1; *X. tropicalis* (tropical clawed frog): NP_989399.1; *M. unicolour* (Tiny Cayenne Caecilian): XP_030070213.1; *D. rerio* (zebrafish): NP_998306.1; *E. electricus* (electric eel): XP_026885649.1.

### AlphaFold2 modelling

Protein models were built using AlphaFold2 via ColabFold v1.5.2^69,70^ using residues 33–116 of human TMEM263 taken from UniProt (Q8WUH6). To maximise the structural sampling, 8 independent seeds were run to generate a total of 40 models. When ranked by pLDDT, the top 5 models all had a similar double helix structure (Fig. 3G) and an average RMSD to the top model of 0.69 ± 0.33 Å. The AlphaFold2 model and all quality metrics are available to download on the Open Science Framework (https://osf.io/sfeur/).

### Atomistic simulations

The AlphaFold2 TMEM263 model was used to seed molecular dynamics simulations. Models were built into simulation systems using CHARMM-GUI^71,72^. Protein atoms were described with the CHARMM36m force field^73,74^. Side chain pKas were assessed using propKa3.1^75^, and side chain side charge states were set to their default. The proteins were built into membranes comprising 4:4:2:1:1 DLPC, DMPC, cholesterol, TAG and DAG. The shorter acyl tails of phospholipids were chosen to represent the thinner membrane of the ER. The membranes were solvated with TIP3P waters and neutralised with K^+^ and Cl^−^ to 150 mM. The simulation boxes were 11 × 11 x 10 nm and had ca. 135,000 atoms.

Each system was minimised and equilibrated according the standard CHARMM-GUI protocol, with a 50 ns final equilibration step. Production simulations were run in the NPT ensemble, with temperatures held at 303.5 K using a velocity-rescale thermostat and a coupling constant of 1 ps, and pressure maintained at 1 bar using a semi-isotropic Parrinello-Rahman pressure coupling with a coupling constant of 5 ps^76,77^. Short-range van der Waals and electrostatics were cut off at 1.2 nm. Simulations were run to 1000 ns with five independent (reseeded) repeats. Lipid contacts were quantified using PyLipID^38^ implemented via LipIDens^78^. Spatial distribution functions for both TAG and DAG were calculated using the GROMACS tool gmx Spatial. Minimum atom-to-phosphate distances were determined with the distance_array function from MDAnalysis, considering all atoms within each residue and calculating the shortest distance to DMPC/DLPC phosphate atoms under periodic boundary conditions. Residue-to-residue distances were measured using the GROMACS tool gmx mindist, focusing exclusively on backbone atoms. Backbone-backbone root mean square deviation (RMSD) calculations were performed with the GROMACS tool gmx RMSD, while the GROMACS tool gmx gangle was employed to compute the angles between the TM helices as well as TM-to-z axis angles. To quantify TIP3 molecules near residues within the 48–80 range, the GROMACS tool gmx mindist was used with a cutoff distance of 0.35 nm, ensuring each TIP3 molecule was counted only once. Protein secondary structure assignments (DSSP) were obtained using the DSSP class from the MDAnalysis.analysis.dssp module. Except for RMSD analyses, the first 100 ns of each trajectory were excluded to allow for equilibration. Data were analysed using GROMACS, v.2023.4^79^.

### Coarse-grained simulations

Coarse-grained systems were built using the AlphaFold2 TMEM263 model. Systems were built using the Martini Maker package of CHARMM-GUI^80^. The proteins were described using Martini 2.2^81,82^. Elastic networks of 500 kJ mol^−1^ nm^−2^ were applied between all protein backbone beads within 1 nm. All systems were solvated with Martini waters and Na^+^ and Cl^−^ ions to a neutral charge and 0.15 M. Minimisation and equilibration followed the standard CHARMM-GUI Martini Maker protocol. Different systems were built, each described separately below.

The TAG blister systems were built using a monomer of TMEM263 in a membrane containing 2.5% TAG and 97.5% POPC. Boxes were set to 16 × 16 x 11.5 nm. Controls without protein were created by deleting the protein beads from the system and equilibrating for 100 ns. Production simulations for each state were run for 2 µs with 20 repeats. TAG clusters were quantified using the GROMACS tool gmx clustsize with a cutoff of 0.55 nm for contacts. For each repeat, every frame was quantified for the total TAG that forms part of the main TAG cluster. Alternatively, a smaller system was built (12 × 12 x 12 nm) containing 59% POPC, 23% cholesterol, and 6% each of cholesterol ester (CE), DAG, and TAG. Post equilibration states were tiled into 9 repeats using the GROMACS tool gmx genconf and simulated for 7 µs to let TMEM263 interact with the CE/DAG/TAG blister. All simulations were run in GROMACS 2022.3^79^. Data were analysed using GROMACS tools and VMD^83^.

### Other software utilised

TMHMM prediction was generated with TMHMM2.0^84^. HeliQuest^85^ was used to visualise amphipathicity of TMEM263 aa35-46 helix 1 (LSRVTGGIFSVT), TMEM263 aa49-68 helix 2 (AVGATIGGVAWIGGKSLEVT), and TMEM263 aa80-102 helix 3 (IGLVKGGVSAVAGGVTAVGSAVV).

### Reporting summary

Further information on research design is available in the Nature Portfolio Reporting Summary linked to this article.

## Supplementary information

Supplementary Information

Peer Review file

Description of Additional Supplementary Files

Supplementary Data 1

Supplementary Movie 1

Reporting Summary

## Source data

Source Data

## Data Availability

All data and reagents will be made available upon request. The mass spectrometry proteomics data have been deposited to the ProteomeXchange Consortium via the PRIDE^86^ partner repository with the dataset identifier PXD083284. Source data are provided in this paper.
