# Supplementary material for: Identification of TMEM263 as an ER-resident protein that controls early stages of lipid droplet biogenesis
Source: Nat Commun. 2026 Sep 28;17:11192. doi: 10.1038/s41467-026-78093-9 (PMC13714300; doi:10.1038/s41467-026-78093-9)
Supplement: Supplementary file 1 [file 41467_2026_78093_MOESM1_ESM.pdf]

Supplementary Information

Identification of TMEM263 as an ER-resident protein that controls early stages of lipid droplet biogenesis

Katy Yalci[1^], Nafsika Chala[1^], Joanna Moss[1], Connor MacDonald[2], Przemysław Zakrzewski[1], Phineas Smith[1], Rabia Sevil[1], Lana Willoughby[1], Seren Wonklyn[1], Thomas Andrews[1], Chrissy Hammond[1], Robin A. Corey[2] & Bernadette Carroll[1*]

[1] School of Biochemistry and Biomedical Sciences, University of Bristol, Biomedical Science Building, BS8 1TD
[2] School of Physiology and Neuroscience, University of Bristol, Biomedical Science Building, BS8 1TD

^ these authors contributed equally

* corresponding author: bernadette.carroll@bristol.ac.uk

This file contains 6 supplementary figures with figure legends and 1 supplementary table

Supplementary Figure 1

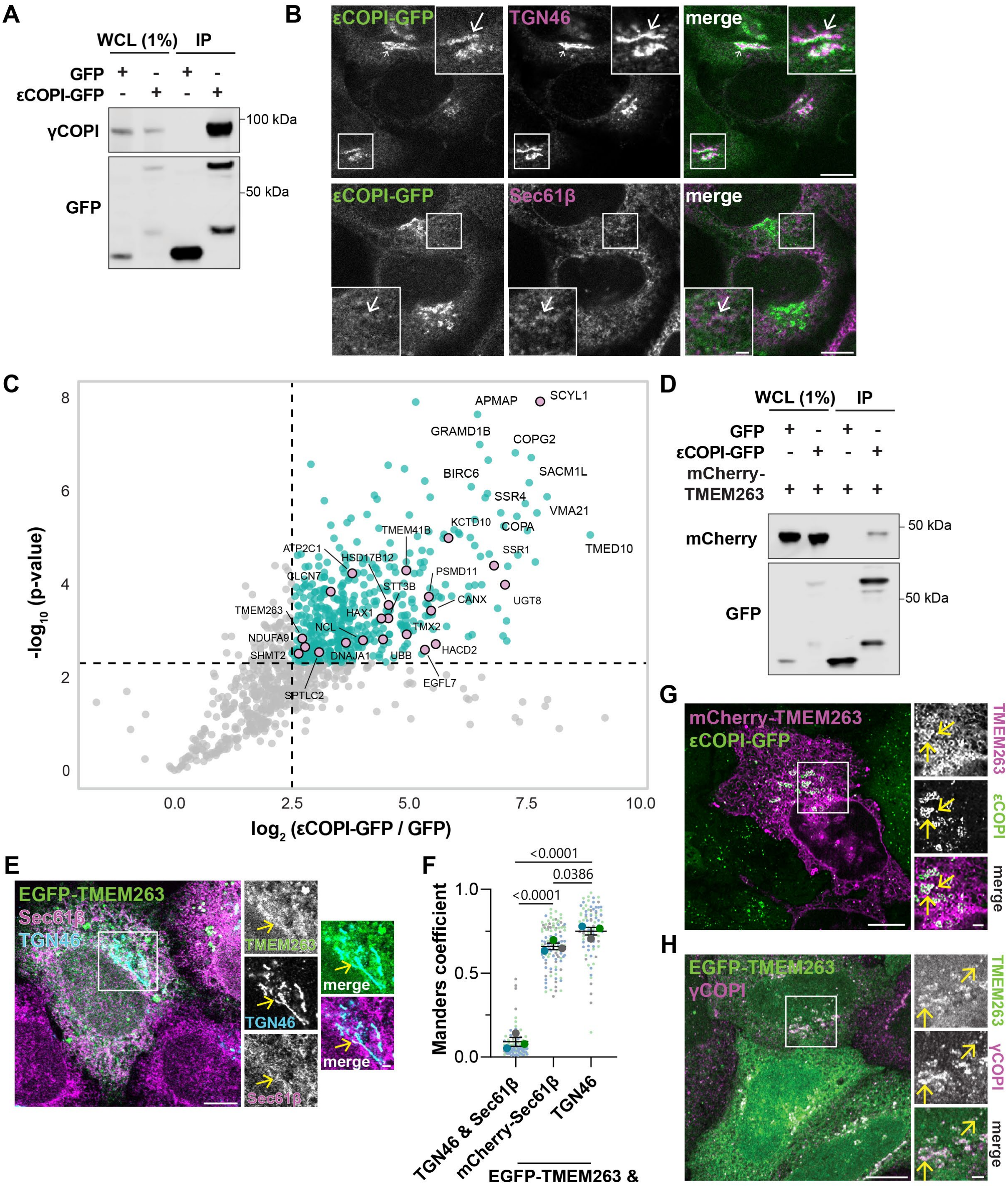

Supplementary Figure 1- Identification of COPI cargo proteins. (A,B) Validation of εCOPI-GFP expression. HeLa cells stably expressing εCOPI-GFP were subject to GFP immunoprecipitation and western blot to confirm binding with endogenous COPI complexes, n = 2, both biological replicates had similar results. **(A)** and colocalises with endogenous TGN46 (trans-Golgi) and Sec61β (ER) **(B)**. White arrows show areas of colocalisation. **(C)** Identification of COPI cargo involved in growth. HeLa cells stably expressing GFP or εCOPI-GFP were subject to GFP immunoprecipitation and analysis by TMT mass spectrometry. Volcano plot of proteins enriched in εCOPI-GFP / GFP immunoprecipitation, proteins in teal represent COPI cargoes, defined as proteins with a $\log_2$ fold change > 2.5, p-value < 0.005 εCOPI-GFP / GFP (n = 4). Proteins labelled on the volcano plot are the top 10 proteins with

canonical COPI-interacting motifs, and those in pink are specifically associated with mammalian growth regulation. **(D)** Validation of εCOPI-GFP interaction with TMEM263. HeLa cells overexpressing mCherry-TMEM263 and εCOPI-GFP were subject to GFP immunoprecipitation and western blotting, n = 2, both biological replicates had similar results. **(E,F,G,H)** TMEM263 localisation is consistent with COPI cargo. HeLa cells expressing EGFP-TMEM263 were fixed and immunostained with antibodies against endogenous Sec61β (ER) and TGN46 (trans-Golgi) **(E)**. Yellow arrows show areas of colocalisation. Quantification of colocalisation of E and Fig. 1C **(F)**. At least 80 cells per condition across n = 3. Ordinary one-way ANOVA with Dunnett's multiple comparisons tests and two-tailed unpaired student t-tests were performed. HeLa cells stably expressing εCOPI-GFP and transiently expressing mCherry-TMEM263 were imaged live, yellow arrows show areas of colocalisation **(G)**. HeLa cells transiently expressing EGFP-TMEM263 were immunostained with an antibody against endogenous γCOPI, yellow arrows show areas of colocalisation **(H)**.

Scale bars are 10 μm and 2 μm for zoomed images. For F, colour coded individual data points correspond to individual cells from different experiments, lines show mean values, and error bars represent SEM.

Supplementary Figure 2

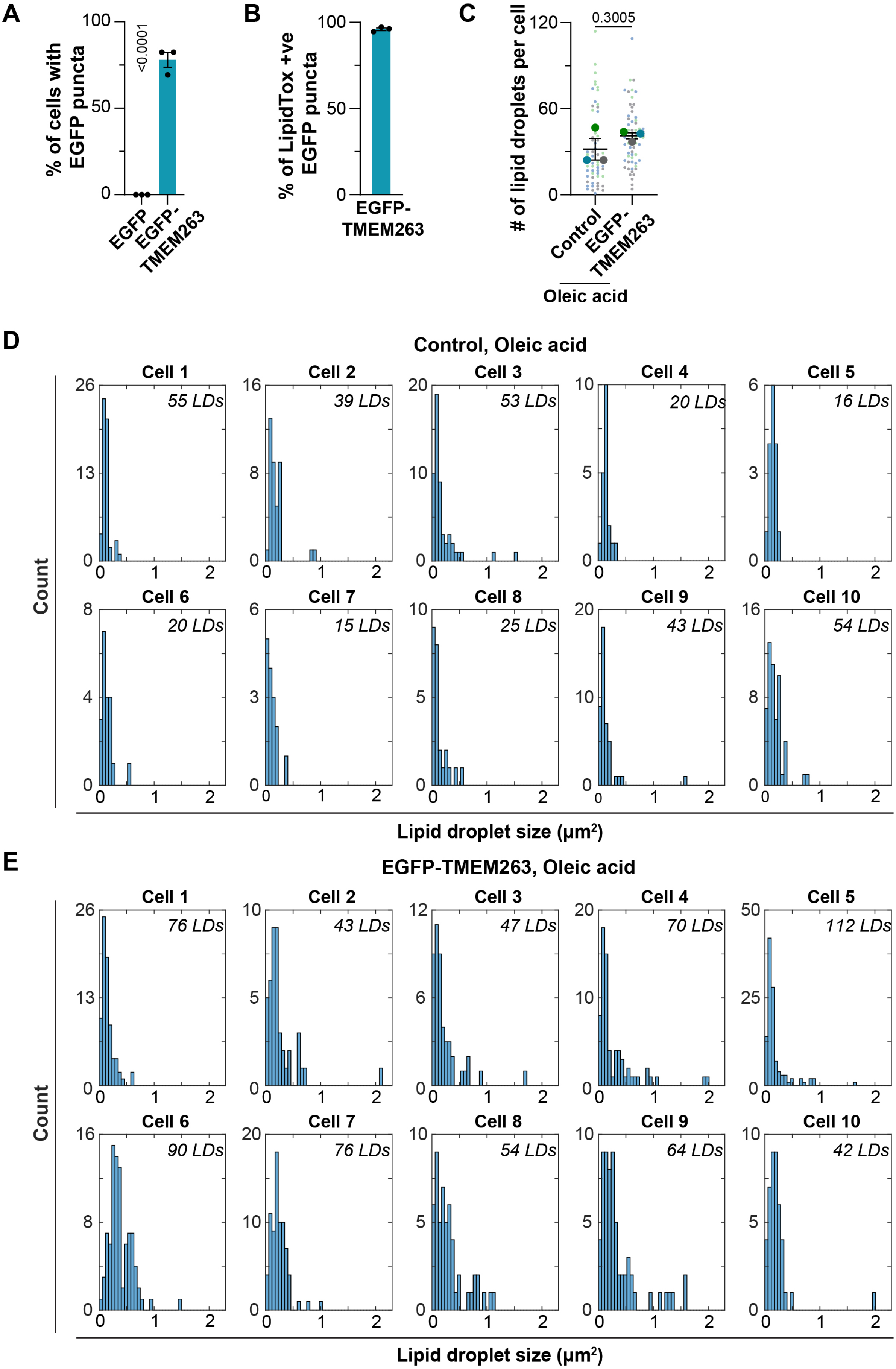

Supplementary Figure 2- Characterising TMEM263 positive vesicles. (A) TMEM263 expression induces puncta formation. Quantification of the % HeLa cells expressing EGFP or EGFP-TMEM263 that contain EGFP puncta. At least 600 cells per condition across n = 3. Cochran-Mantel-Haenszel chi-squared tests with Haldane-Anscombe and Bonferroni corrections was performed. **(B)** EGFP-TMEM263 puncta are lipid droplets. Quantification of the % EGFP-TMEM263 puncta of HeLa cells that are LipidTOX-positive. At least 100 cells and 1500 puncta per condition across n = 3. **(C)** Overexpression of EGFP-TMEM263 does not alter OA-induced lipid droplet numbers. The number of lipid droplets per cell using the LipidTOX signal was quantified from HeLa cells treated as in D. Control represents untransfected cells in the same field of view as EGFP-TMEM263-expressing cell. At least 60 cells per condition across n = 3. Two-tailed unpaired student t-test was performed. **(D,E)** Distribution of lipid droplet size. HeLa cells were transfected with EGFP-TMEM263, treated for 16 hours with 30 μM oleic acid and stained with LipidTOX as in (D). Lipid droplet size was quantified for transfected **(E)** and untransfected control **(D)** using a maximum z-projection of a z-stack. Control represents untransfected cells in the same field of view as EGFP-TMEM263-expressing cell. Histograms of lipid droplets size of 10 cells per condition of one experiment are shown, the number of lipid droplets detected per cell are written in the top right corner.

Unless stated otherwise, n = 3. Scale bars are 10 μm and 2 μm for the zoomed in images. Bars extend to the mean, colour coded individual data points correspond to individual cells from different experiments (C), lines show mean values, and error bars represent SEM.

Supplementary Figure 3

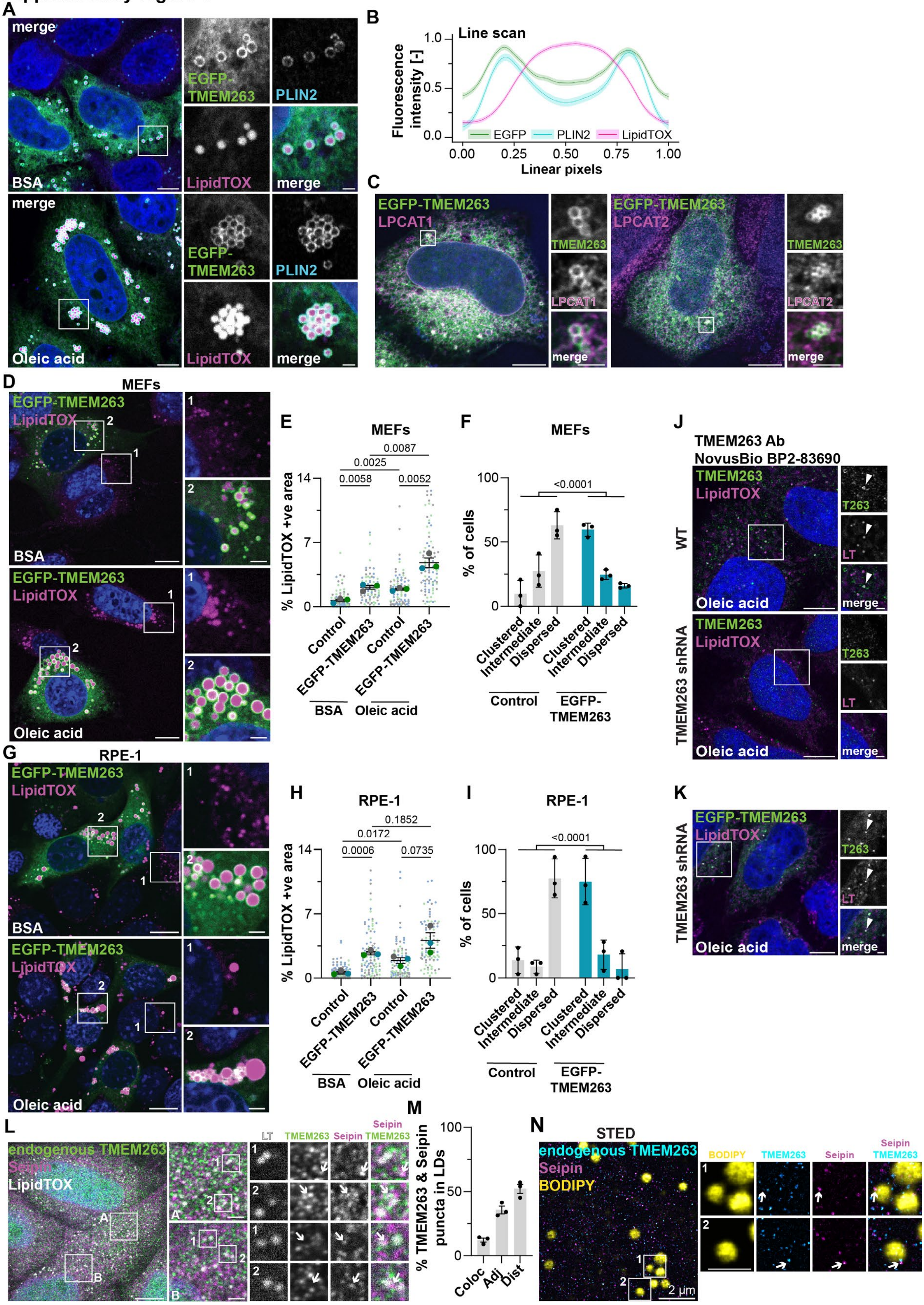

Supplementary Figure 3- Characterising TMEM263 localisation. (A) Expression of TMEM263 induces the accumulation of lipid droplets. HeLa cells expressing EGFP-TMEM263 were incubated for 16 hours with BSA vehicle control or 30 µM oleic acid, fixed, stained with

LipidTOX and immunostained with an antibody against endogenous perilipin 2 (PLIN2). **(B)** TMEM263 colocalises with PLIN2 at lipid droplet membranes. Quantification of EGFP, PLIN2 and LipidTOX signal from 30 linescans of (A, oleic acid) through individual puncta. Shading corresponds to 95% confidence interval, data fitted using Loess regression. **(C)** TMEM263 colocalises with lipid droplet proteins. HeLa cells expressing EGFP-TMEM263 were fixed and immunostained with antibodies against endogenous LPCAT1 and LPCAT2. **(D-I)** TMEM263 induces the accumulation and clustering of lipid droplets in multiple cell lines. Mouse Embryonic Fibroblasts (MEFs) **(D-F)** and Retinal Epithelial (RPE-1) **(G-I)** cells were incubated for 16 hours with BSA vehicle control or 30 μM oleic acid, fixed and stained with LipidTOX. The % LipidTOX positive area per cell and the % cells with clustered, intermediate or dispersed EGFP-TMEM263-positive lipid droplet distribution were quantified. For H, 2 outliers are out of range of axis. **(J)** TMEM263 antibody validation for immunostaining. HeLa cells with shRNA scr (WT) or shRNA TMEM263 were treated with 30 μM oleic acid for 16 hours, fixed, immunostained with antibody against endogenous TMEM263 (NovusBio BP2-83690) and stained with LipidTOX. Arrowhead points to TMEM263 positive lipid droplet. **(K)** HeLa cells with shRNA TMEM263 were transfected with EGFP-TMEM263 and treated with 30 μM oleic acid for 16 hours, fixed and stained with LipidTOX. Arrowhead points to TMEM263 positive lipid droplet. **(L-N)** Endogenous TMEM263 does not co-occur with lipid droplet biogenesis regulator, seipin. Representative images of HeLa cells treated with 30 μM oleic acid for 16 hours. Cells were fixed and immunostained with antibodies against endogenous TMEM263 and seipin, and stained with LipidTOX **(L)** or BODIPY **(N)**. For L, cells were imaged using Spinning disk confocal microscope, for N, TMEM263 and seipin are imaged in 2D STED mode and BODIPY is imaged in confocal mode. White arrows point to seipin positive puncta. Scale bars are 2 μm and 1 μm for the zoomed in images. **(M)** Quantification of distance between TMEM263 and Seipin puncta linked to the same lipid droplet and divided in the following categories: colocalised (Coloc), adjacent (Adj), and distinct (Dist). Images as in J were used.

Unless stated otherwise, n = 3. Scale bars are 10 μm and 2 μm for the zoomed in images. Bars extend to the mean, colour coded individual data points correspond to individual cells from different experiments (E and H), lines show mean values, and error bars represent SEM. For E and H, two-tailed unpaired student t-tests were performed. For F and I, Cochran-Mantel-Haenszel chi-squared tests with Haldane-Anscombe and Bonferroni corrections were performed.

Supplementary Figure 4

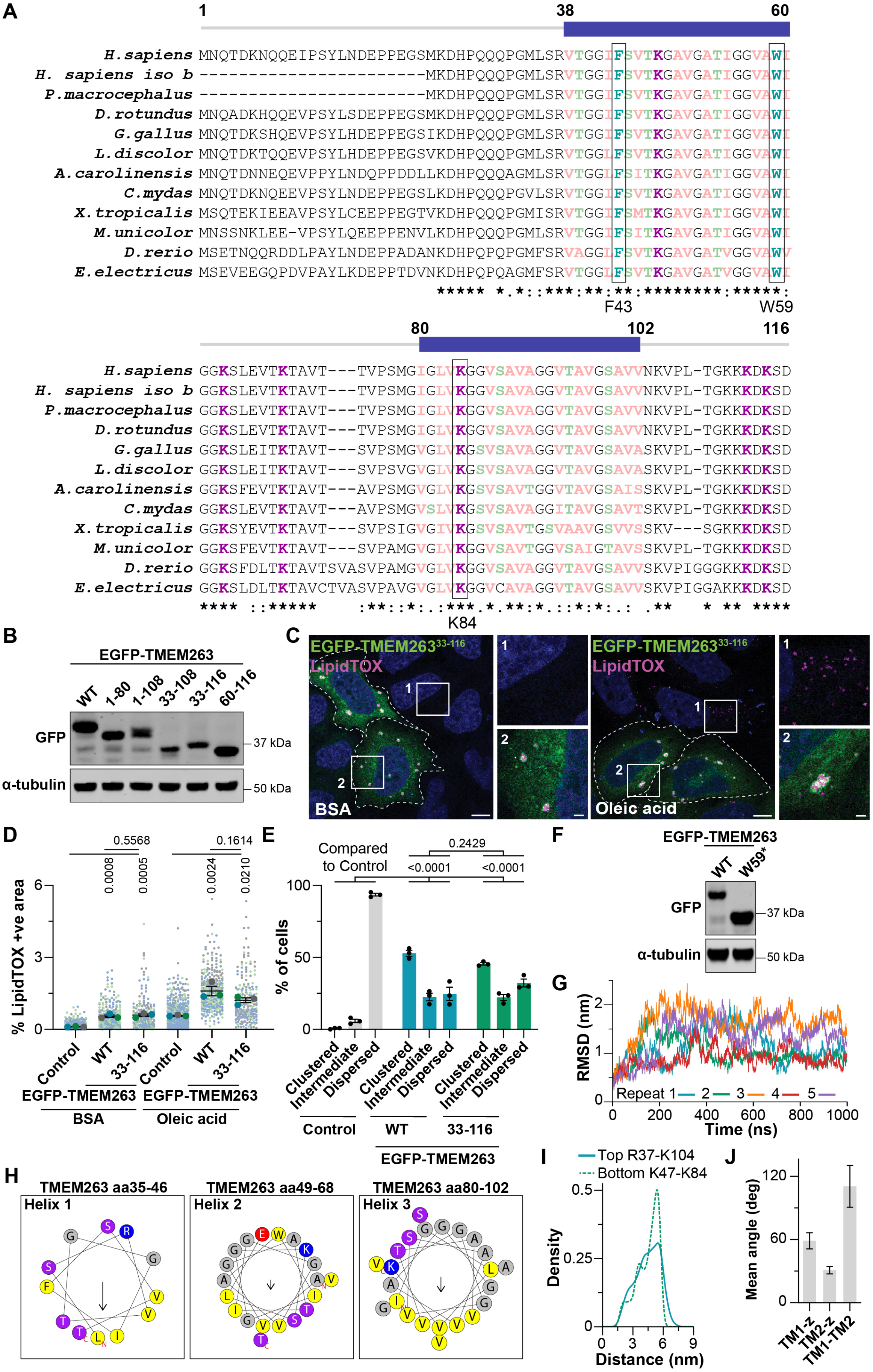

Supplementary Figure 4- Identifying functional domains of TMEM263. (A) Multiple sequence alignment of TMEM263 highlights the two transmembrane domains are highly conserved. **(B)** Representative blot showing the expression levels of EGFP-TMEM263 truncations shown in Fig. 3C,D. n = 2, both biological replicates had similar results. **(C-E)** N-terminus of TMEM263 is functionally redundant. HeLa cells expressing EGFP-TMEM263 full-length or EGFP-TMEM263$^{33\text{-}116}$ were incubated for 16 hours with BSA vehicle control or 30 µM oleic acid, fixed and stained with the neutral lipid dye, LipidTOX **(C)**. The % LipidTOX positive area per cell was quantified **(D)**. Ordinary one-way ANOVA with Dunnett's multiple comparisons tests and two-tailed unpaired student t-tests were performed. Control represents untransfected cells in the same field of view as EGFP-TMEM263-expressing cells. Data from control and EGFP-TMEM263^{WT}-expressing cells are also included in Fig. 2C. Cells were scored as having a clustered, intermediate, or dispersed distribution of EGFP-TMEM263-positive lipid droplets **(E)**. Each category is expressed as a percentage of total cells. At least 200 cells per condition across n = 3. Cochran-Mantel-Haenszel chi-squared tests with Haldane-Anscombe and Bonferroni corrections were performed. **(F)** Representative blot showing the expression levels of EGFP, EGFP-TMEM263^{WT} and EGFP-TMEM263^{W59*} shown in Fig. 3E,F. n = 2, both biological replicates had similar results. **(G)** RMSD of Cα atoms relative to the initial frame plotted over 1000 ns for five independent repeat simulations (Repeats 1-5, coloured individually). **(H)** Helical wheel diagrams of MD predicted Helix 1 (aa35-46), Helix 2 (aa49-68), and Helix 3 (aa80-102) were generated using the HeliQuest. **(I)** Distance distributions between Cα atoms of basic residues across the bilayer leaflets from atomistic MD simulations. Kernel density estimates of the distance between R37-K104 (top leaflet, solid line) and K47-K84 (bottom leaflet, dashed line). **(J)** Mean tilt angle of TM domain 1 (TM1) and TM domain 2 (TM2) relative to the bilayer normal (z-axis) and of TM1 relative to TM2 from atomistic MD simulations. Error bars represent standard deviation across five independent repeat simulations.

Unless stated otherwise, n = 3. Scale bars are 10 µm and 2 µm for the zoomed in images, bars extend to the mean, colour coded individual data points correspond to individual cells from different experiments (D), lines show mean values, and error bars represent SEM. For G,I,J analysis was taken over 5 repeats of 1 µs. For I and J, the first 100 ns were discarded as equilibration.

Supplementary Figure 5

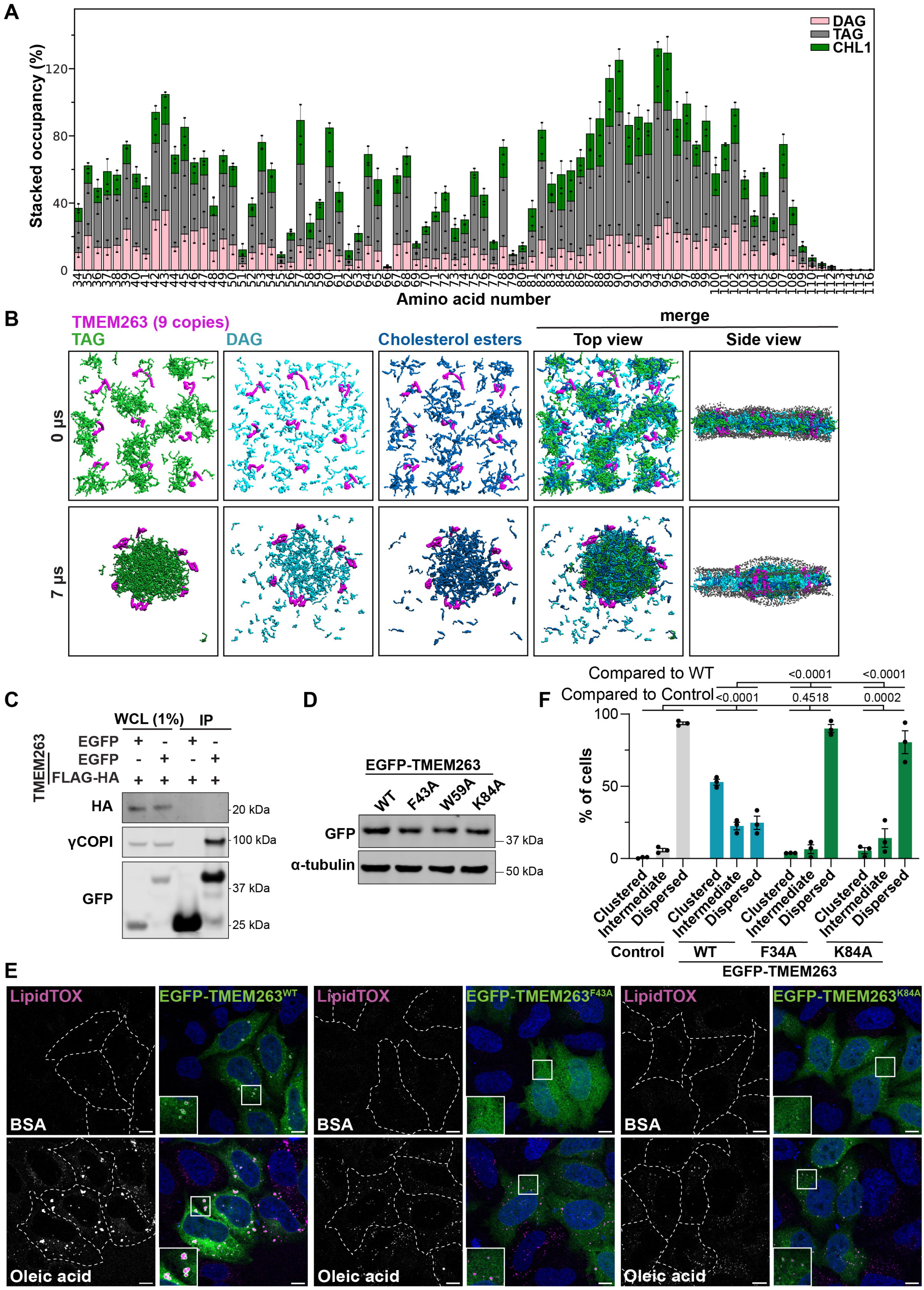

Supplementary Figure 5- Characterising TMEM263 lipid binding. A) PyLipID-derived TAG, DAG and cholesterol (CHL1) binding occupancies based on atomistic MD. Occupancies were calculated for each residue as a % of frames where the residue is within 0.35 nm of

TAG/DAG/cholesterol. Data are given for each residue and error bars represent SD, n = 5. **(B)** TMEM263 is recruited to lipid lens in a phospholipid bilayer. Coarse-grained simulation of 6% TAG (green), 6% DAG (cyan), and 6% cholesterol esters (blue) in a phospholipid bilayer with 9 molecules of TMEM263$^{33\text{-}116}$ (magenta). Phosphate heads are shown in grey in the side view. Views are shown both before (0 µs) and after (7 µs) simulation. **(C)** TMEM263 shows no evidence of oligomerisation. HEK293T cells expressing EGFP-TMEM263 and FLAG-HA-TMEM263 were subject to GFP immunoprecipitation and western blot, n = 2, both biological replicates had similar results. **(D)** Representative blot showing the expression levels of EGFP-TMEM263 mutations shown in E and Fig. 4E,F. n = 2, both biological replicates had similar results. **(E,F)** HeLa cells were transfected with EGFP-TMEM263 mutants as indicated, were incubated for 16 hours with BSA vehicle control or 30 µM oleic acid, fixed and stained with the neutral lipid dye, LipidTOX **(E)**. Cells were scored as having clustered, intermediate, or dispersed lipid droplets **(F)**. At least 250 cells per condition across n = 3. Cochran-Mantel-Haenszel chi-squared tests with Haldane-Anscombe and Bonferroni corrections were performed.

Scale bars are 10 µm and 2 µm for zoomed images, bars extend to the mean, lines show mean values, and error bars represent SEM, unless stated otherwise.

Supplementary Figure 6

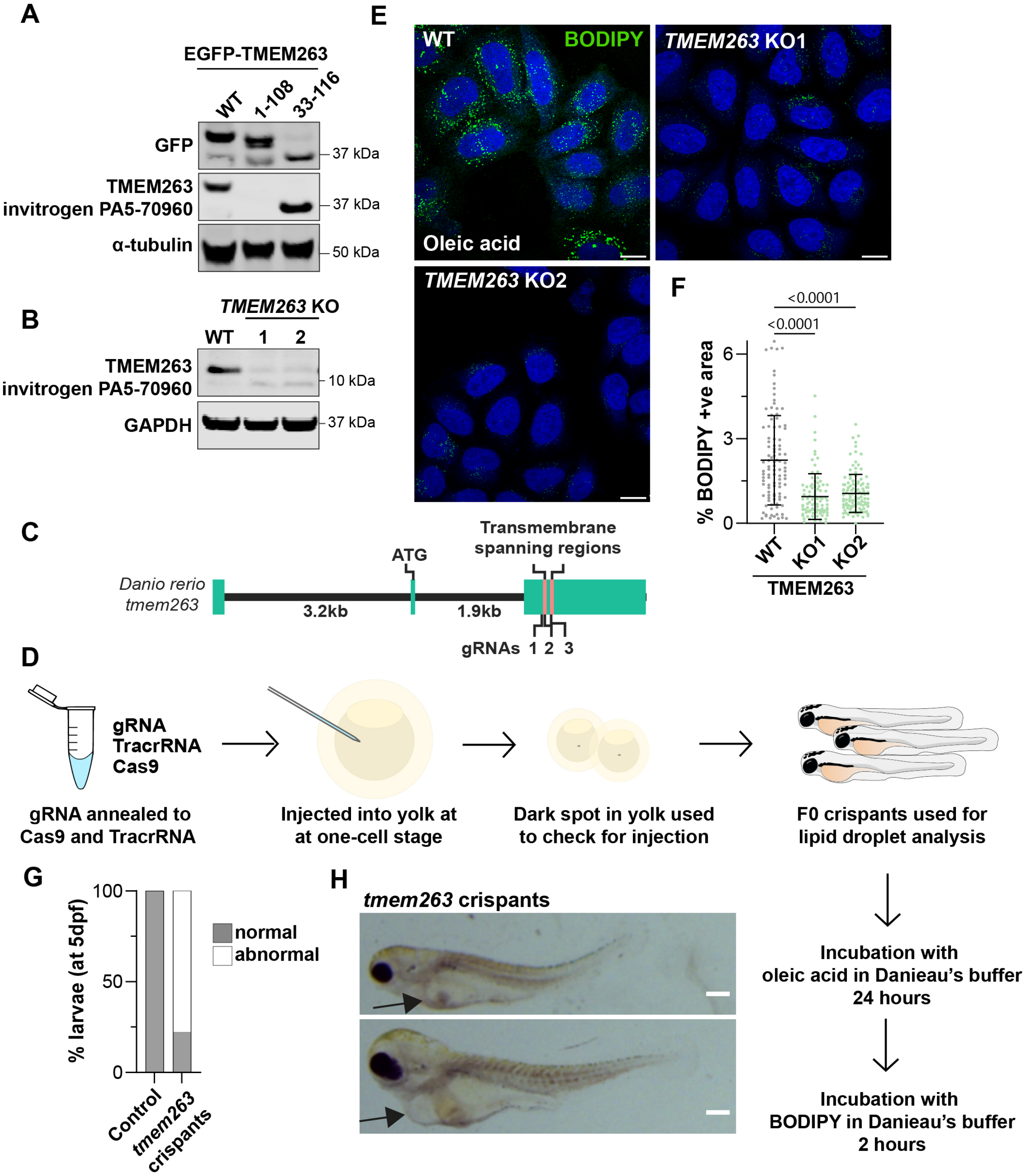

Supplementary Figure 6- Generation of TMEM263 models. (A) Validation of TMEM263 antibody (Invitrogen PA5-70960) for use in western blot. HeLa cells were transfected with EGFP-TMEM263^{WT}, EGFP-TMEM263$^{1\text{-}108}$, or EGFP-TMEM263$^{33\text{-}116}$ and blotted. Antibodies for both GFP and TMEM263 were used. The endogenous antibody recognises the C-terminal of TMEM263, n = 2, both biological replicates had similar results. **(B)** Generation of *TMEM263* Crispr/Cas9 knock-out (KO) HeLa cells. Representative blot showing loss of TMEM263 protein, n = 3, all 3 biological replicates had similar results. **(C,D)** Strategy to create *tmem263* mutant zebrafish. gRNA were generated to target the transmembrane domains of TMEM263 **(C)** and the timeline to create a crispant zebrafish cohort **(D)**. Parental, *TMEM263* KO1 and *TMEM263* KO2 HeLa cells were treated for 16 hours with 30 μM oleic acid and stained with BODIPY, scale bar is 10 μm **(E)**. The % BODIPY-positive area per cell was quantified **(F)**.

Graph shows representative data from one repeat, n = 3 with at least 90 cells per condition. Individual data points correspond to individual cells, lines show mean values, error bars represent SD. Two-tailed Kruskal-Wallis test with Dunn's multiple comparisons tests was performed. All 3 biological replicates had similar results. **(G,H)** *tmem263* mutation impairs zebrafish larval development. The proportion of zebrafish in control or *tmem263* crispant groups were quantified for normal or abnormal larval development $n_{normal} = 19$ for control and $n_{normal} = 12$, $n_{abnormal} = 41$ for *tmem263* crispants. **(G)**. Representative image of two *tmem263* crispant larvae (5dpf) with characteristic oedema (black arrow), scale bars are 250 μm **(H)**.

	Forward primer:	Reverse primer:
pEGFP-C2-TMEM263$^{1-80}$	TAAGCAGAATTCATGAATCAGACAGATAAAAATCAACAAG	TGCTTAGGATCCTCATATTCCCATGGAAGGCACAGTTG
pEGFP-C2-TMEM263$^{1-108}$	TAAGCAGAATTCATGAATCAGACAGATAAAAATCAACAAG	TGCTTAGGATCCTCATGTTAAGGGCACTTTGTTTAC
pEGFP-C2-TMEM263$^{33-108}$	TGCTTAGAATTCGGCATGTTGTCCCGTGTG	TGCTTAGGATCCTCATGTTAAGGGCACTTTGTTTAC
pEGFP-C2-TMEM263$^{33-116}$	TGCTTAGAATTCGGCATGTTGTCCCGTGTG	TGCTTAGGATCCTCAGTCAGATTTGTCTTTCTTCTTTCC
pEGFP-C2-TMEM263$^{60-116}$	TGCTTAGAATTCATTGGTGGAAAGAGTCTGG	TGCTTAGGATCCTCAGTCAGATTTGTCTTTCTTCTTTCC
pEGFP-C2-TMEM263^{W59*}	GACTCTTTCCACCAATCTAAGCCACACCACCAATG	CATTGGTGGTGTGGCTTAGATTGGTGGAAAGAGTC
pEGFP-C2-TMEM263^{K114A}	CGGTGGATCCTCAGTCAGATGCGTCTTTCTTCTTTCCTGTTA	TAACAGGAAAGAAGAAAGACGCATCTGACTGAGGATCCACCG
pEGFP-C2-TMEM263^{K112A}	GATCCTCAGTCAGATTTGTCTGCCTTCTTTCCTGTTAAGGGCAC	GTGCCCTTAACAGGAAAGAAGGCAGACAAATCTGACTGAGGATC
pEGFP-C2-TMEM263^{K111A}	CCTCAGTCAGATTTGTCTTTCGCCTTTCCTGTTAAGGGCACTTT	AAAGTGCCCTTAACAGGAAAGGCGAAAGACAAATCTGACTGAGG
pEGFP-C2-TMEM263^{K110A}	CTCAGTCAGATTTGTCTTTCTTCGCTCCTGTTAAGGGCACTTTGTTTA	TAAACAAAGTGCCCTTAACAGGAGCGAAGAAAGACAAATCTGACTGAG
pEGFP-C2-TMEM263^{W59A}	GACTCTTTCCACCAATCGCAGCCACACCACCAATGG	CCATTGGTGGTGTGGCTGCGATTGGTGGAAAGAGTC
pEGFP-C2-TMEM263^{K84A}	GCAGAGACACCCCCTGCCACCAGCCCTATTCC	GGAATAGGGCTGGTGGCAGGGGGTGTCTCTGC
pEGFP-C2-TMEM263^{F43A}	GCTCCCTTTGTAACACTGGCGATACCCCCAGTCACACG	CGTGTGACTGGGGGTATCGCCAGTGTTACAAAGGGAGC

Supplementary Table 1: Primers used to generate TMEM263 truncations and mutations.
