# Supplementary material for: Identification of TMEM263 as an ER-resident protein that controls early stages of lipid droplet biogenesis
Source: Nat Commun. 2026 Sep 28;17:11192. doi: 10.1038/s41467-026-78093-9 (PMC13714300; doi:10.1038/s41467-026-78093-9)
Supplement: Supplementary file 2 [file 41467_2026_78093_MOESM2_ESM.pdf]

nature portfolio

Peer Review File

Identification of TMEM263 as an ER-resident protein that controls early stages of lipid droplet biogenesis

Corresponding Author: Dr Bernadette Carroll

This file contains all reviewer reports in order by version, followed by all author rebuttals in order by version.

Version 0:

Reviewer comments:

Reviewer #1

(Remarks to the Author)
Comments:

TMEM263 has been found to be closely associated with cellular growth and human body development, yet its mechanistic role remains unclear. In this study, the authors discovered that TMEM263 is an ER-resident protein that localizes to the ER through a COPI-dependent mechanism and participates in lipid droplet (LD) biogenesis. Through its transmembrane domains, TMEM263 binds to neutral lipids and promotes lipid droplet formation. Loss of TMEM263 leads to reduced lipid droplet accumulation and growth defects in zebrafish.

While the function of TMEM263 is clearly linked to LD formation, mechanistic analysis appears to be preliminary. It is rerecommended that the authors consider additional experiments and discussion on the following issues:

1) The introduction of TMEM263 is through a screen for COPI binding proteins, which basically selects potential ER and Golgi-resident proteins (note that COPI also works to retain Golgi-resident proteins). It is not logically fluent with the following focus on TMEM263 and LD biogenesis. It is suggested that this part to be tuned down and possibly moved to supplementary info.

2) The authors propose that TMEM263 functions during lipid droplet (LD) biogenesis and may translocate from the ER to LDs. While LipidTOX is commonly used to label mature LDs, the authors could employ other probes or marker proteins (such as GFP-GPAT4-TM or GFP-DFCP1) to specifically label nascent LDs and examine whether TMEM263 localizes to these nascent structures. Furthermore, live-cell time-lapse imaging of GFP-tagged TMEM263 would be essential to directly observe its dynamic translocation from the ER to LDs.

3) The TMEM263 hairpin as predicted by multiple methods, is not a typical hairpin. The connecting loop of the two TM in the luminal side is simply too long and not feasible for monolayered phospholipids on the surface of LD. In fact, the simulation is done with bilayer. It contains multiple hydrophilic residues (K63, S64, E66, T68, K69, T70, T73/74, S77) between its two transmembrane domains, which would be energetically unfavorable for insertion into the hydrophobic core of LDs (Fig 4B shows lower lipid binding properties in this region). Unlike PLIN1 and UBXD8/FAF2 (which are not full transmembrane proteins) or GPAT4 (which has only a short base and features a V-shaped hydrophobic hairpin structure), how could TMEM263 insert into LD membrane remains unclear. An in vitro reconstitution assay using purified TMEM263 and artificial LDs could determine whether TMEM263 can incorporate into the LD phospholipid monolayer.

4) The authors did not detect the interaction between TMEM263 and seipin, and notably, TMEM263 forms 2-3 punctate structures on LD surfaces that are distinct from seipin's localization. More conclusive evidence is needed to clarify TMEM263's specific role in mediating ER-LD contact sites and facilitating LD biogenesis or growth.

5) Fig. 5F: BODIPY staining is less potent than LipidTOX; it is recommended to switch to LipidTOX for lipid droplet labeling to align with the cell experiments.

6) In Fig. 2I, the authors used an endogenous antibody to stain TMEM236. The specificity of the antibody needs to be demonstrated in the sup info, possibly a control staining using KO cells.

Reviewer #2

(Remarks to the Author)
The manuscript explores the role TMEM263, an ER-resident protein, in the early stages of lipid droplet biogenesis. It convincingly demonstrates how the protein's structural features, particularly its two transmembrane domains, facilitate interactions that promote neutral lipid condensation, thereby driving lipid droplet initiation and growth. By employing both cellular and zebrafish models, the study provides strong evidence that loss of TMEM263 disrupts lipid droplet formation and leads to growth abnormalities. These findings are significant, as they establish a novel mechanistic link between lipid homeostasis, organismal growth, and skeletal development. The manuscript is based on solid experimental work and presents compelling data with moderately clear interpretations. That said, there are a number of important concerns that need to be addressed before the manuscript can reach its full potential.

Comments on the Manuscript
1. Whether that tag used with TMEM263 is GFP or EGFP? If its EGFP the name should be changed accordingly for all chimeric proteins.
2. The statement "When the neutral lipid accumulation surpasses a critical concentration (~5–10 mol%), they undergo phase separation into a lipid 'lens'" requires a supporting reference for the critical concentration threshold. Please insert an appropriate citation.
3. The claim of colocalization of GFP-TMEM263 with Sec61 or TGN46 in Figure 1 would be strengthened by quantitative analysis (e.g., pairwise cross-correlation). Appropriate negative controls (two unrelated images, e.g., ER vs. Golgi) and positive controls (two acquisitions of the same GFP-TMEM263 image) should also be included.
4. The fraction of cells exhibiting the "bunch-of-grapes" phenotype in both control and GFP-TMEM263–expressing cells should be quantified. In addition, this phenotype is not observed in MEFs and RPE-1 cells, which should be noted in the Results section.
5. In several places, GFP-TMEM263 is incorrectly referred to as TMEM263. This may cause confusion with the endogenous protein. For example, the sentence "TMEM263-positive lipid droplets further confirms TMEM263 is recruited to lipid droplet membranes (Fig. S2E)" should be corrected to specify GFP-TMEM263.
6. While colocalization of GFP-TMEM263 with multiple LD-resident proteins and LipidTOX has been shown, the conclusion that TMEM263 is definitively an LD-resident protein is premature. Either this should be stated more cautiously, or higher-resolution imaging (e.g., TEM or SEM) could be used to confirm localization.
7. In Figure S1B, a higher-resolution, zoomed-in image should be provided to clearly demonstrate colocalization of GFP-esp-COP1 with the ER and Golgi. It would be clearer to present these as pairwise comparisons (e.g., EGFP-esp-COP1 with ER [red/green pair] and EGFP-esp-COP1 with Golgi [red/green pair]).
8. In Figure 4G, the corresponding images of mutant expression should be included.
9. Regarding the statement "mutant GFP-TMEM263 positive puncta was significantly reduced compared to wild-type," the authors should first demonstrate that expression levels of each mutant are comparable to wild-type.
10. Similarly, in Figure 4G, expression levels of all mutants should be shown to be comparable.
11. Is there experimental evidence that 2.5% TAG concentration is indeed below the threshold for spontaneous lipid coalescence? Please clarify.
12. The image quality in Figure 1C needs improvement to convincingly demonstrate colocalization. Pairwise presentation would enhance clarity.
13. In the sentence "As expected, GFP-TMEM263 colocalised with the ER, Golgi (Fig. 1C) and endogenous COPI (Fig. 1D), the latter at small vesicular structures and at a region consistent with the Golgi," the vesicular structures should be indicated in the figure with arrows.
14. From Figures 2I and 2J, it cannot be conclusively stated that endogenous TMEM263 is LD-resident. This should be more cautiously discussed in the manuscript.
15. The localization pattern of GFP-TMEM263 (Figures 2B, 2C, 2G, 2H) appears different from that of endogenous TMEM263 (Figures 2I, 4A). Could the GFP tag itself be affecting localization? This should be addressed.
16. The mechanism by which proteins containing transmembrane domains localize to LDs should be discussed in the manuscript.
17. The claim that TMEM263-positive LDs are larger than controls (Figure 2) is not sufficiently supported. Representative images alone are inadequate. A quantitative analysis of LD size distribution should be performed in at least 15–20 randomly selected control cells. Similar analyses should be carried out in GFP-TMEM263–expressing cells at varying expression levels (high, medium, low; controlled by transfection DNA amounts). Expression levels of GFP-TMEM263 should also be quantified. Intra-cellular and inter-cellular variations of LD sizes should be quantified.
18. To confirm that GFP-TMEM263 localization to LDs is not an artifact of overexpression, the authors should examine GFP-TMEM263 localization after endogenous TMEM263 knockdown. This would help establish whether GFP tagging alters localization.
19. The inference of colocalization between endogenous TMEM263 and seipin/BSCL2 (Figure 4A) is not sufficiently supported by the presented image. Quantitative colocalization analysis is needed. A negative control (e.g., TMEM263 with a protein forming puncta but unrelated to LDs, such as TNFR1) should also be included.
20. In Figure 5, TMEM263 knockdown in HeLa cells appears to reduce LD size rather than number. Since quantification of LD number may be unreliable when LD size decreases, a more rigorous analysis is required. Higher-magnification imaging and 3D imaging should be performed to quantify both LD number and size accurately.
21. The zebrafish blastodisc contains LDs (see: https://pubmed.ncbi.nlm.nih.gov/26355567). It would be valuable to analyze LD size and number distribution in tmem263 crispants at the 1- and 2-cell stages.

Reviewer #3

(Remarks to the Author)
The manuscript by Yalci et al. identifies TMEM263 as a novel ER-resident COPI cargo protein essential for early lipid droplet (LD) biogenesis, proposing a hairpin topology that mediates ER bilayer integration and LD targeting. While the study combines AlphaFold predictions, molecular dynamics (MD) simulations, and cellular assays, the data in its current form provide limited new mechanistic insights and largely reiterate previous findings in the LD biology field. Critical gaps include insufficient experimental validation of structural models, short simulation timescales, lack of monolayer simulations, and limited functional evidence for early LD biogenesis. Addressing these issues with extended simulations, biochemical confirmation, and dynamic imaging would significantly strengthen the study and make it more suitable for publication in Nature Communications. In its present form, the manuscript provides limited novel mechanistic understanding of ER-to-LD partitioning of class I LD proteins and largely reiterates findings already reported in the journal.

Major Comments:
Comment 1: In Figure 3C, the author stated that the overexpression of protein truncations of GFP-TMEM263 in HeLa cells is required for proper localization to the ER and induces the accumulation of TMEM263-positive LDs. However, in the gray-scale images, it is hard to visualize the accumulation of TMEM263-positive LDs. Also, the LD clustering/accumulation phenotype appears to be relatively weak.
Comment 2: Based on the limited co-localization of endogenous TMEM263 with seipin on a subset of LDs in Figure 4A, the suggestion that TMEM263 functions in the early stages of LD formation implied in the manuscript title requires further experimental support. I will suggest employing live-cell imaging using early LD biogenesis markers such as the model peptide HPos, LiveDrop, or acyl-CoA synthetase 3, which is known to localize to nascent LD assembly sites and is essential for efficient LD nucleation (PMID: 24368806).
Comment 3: While AlphaFold has transformed structure prediction, its application to membrane proteins remains limited, especially when lipid-dependent conformational changes are involved. The static nature of AlphaFold's predictions does not account for dynamic structural rearrangements driven by membrane composition or lipid-protein interactions. Therefore, in Figure 4B, the predicted hairpin structure needs to be experimentally verified using biochemical and biophysical experiments, as done elegantly in this study (PMID: 38802378), to eliminate the possibility that the protein does not associate with the ER via an amphipathic helix and indeed adopts a hairpin topology. Additionally, the authors should display helical wheel projection for each hairpin in the figure.
Comment 4: The authors conducted relatively short 150 ns simulations to investigate TMEM263-neutral lipid/bilayer interactions. Given that the peptide structure was predicted using AlphaFold and considering the slow diffusion rates of lipids, the simulation timeframe provides insufficient sampling, even with five replicate simulations. The limited diffusion of lipids within this timeframe raises concerns about the statistical significance of the observed interactions.
Furthermore, the authors did not experimentally validate the conformation of the peptide in the lipid membrane. Snapshots at different time points clearly demonstrate that the peptide conformation has not reached equilibrium or stabilized within the simulation period. The use of shorter acyl tails in DLPC/PE lipids to represent the thinner ER membrane further raises questions about the validity of the observed peptide structure and conformation in the bilayer.
There is a discrepancy in the methods section where the authors claim to have simulated up to 150 ns; however, the final structure shown in Figure S4, D is at 94.4 ns. It is not clear what happened to the peptide structure between 94-150 ns. This raises concerns about the stability and reliability of the reported conformations. The use of AlphaFold-predicted structures with short simulations (100-150 ns) is insufficient for drawing reliable conclusions about TMEM263-membrane interactions. Previous studies on UBXD8 (PMID: 38802378) employed more extensive MD simulations at the microsecond timescale, complemented by solid experimental data to validate the structure in the bilayer and monolayer. Authors should extend simulations to the microsecond timescale to provide meaningful structural insights into TMEM263-membrane interactions and include experimental data to support the computational findings.
The authors claim that peptides maintained the same orientation and remained evenly spaced around the lens with no evidence of oligomerization. However, several methodological concerns need to be addressed: In the context of LD biogenesis, where seipin is a key regulator, the authors should clarify the biological relevance of evenly spaced peptides around the lens. It is not surprising that peptides maintain their orientation in coarse-grained Martini simulations, as these simulations employ an elastic network that inherently restricts conformational changes in proteins. Martini 2 is known to produce artificially strong protein-protein interactions ("sticky" proteins). The article does not clarify whether: (a) Protein-protein interactions were scaled to reduce artificial aggregation; and (b) If peptide interactions with the lens reduce diffusion and artificially restrict aggregation. The authors should provide more detailed structural insights to support their claims about peptide behavior and arrangement. In addition, authors consider using Go-Martini and other Martini models to follow the conformational changes. These methodological considerations are crucial for properly interpreting the observed peptide behavior and its relevance to LD biogenesis mechanisms.
Another major limitation of this study is the absence of simulations in a LD-like monolayer environment. TMEM263 is proposed to localize to or interact with LDs, which are characterized by a phospholipid monolayer rather than a bilayer. Simulating the protein only within a bilayer membrane environment fails to recapitulate the biophysical constraints and properties like lipid packing and surface tension unique to LDs. This oversight may mask conformational changes or membrane-embedding modes that are specific to TMEM263 LD localization. Therefore, incorporating simulations in a lipid monolayer environment would be essential to better understand the structural dynamics and topological behavior of TMEM263 in its physiological context.
Comment 5: The protein, as shown in Figure 4C and Figure S4 (A), accumulates near the LD rim and never transitions to the LD. Considering the high contact of neutral lipids to amino acids W59 and K84 (Figure 4E), does this interaction facilitate the switch in conformation of the TMEM263 from its deeply buried state within the bilayer to help it transition to the LDs? Due to the absence of this critical data, their MD simulations are just re-validating the earlier work (PMID: 32730754; PMID: 38802378; PMID: 35583926; PMID: 37889293), and not providing any new structural insights. The current gap in knowledge

is in identifying the factors that drive the partitioning of hairpin proteins from the bilayer to the monolayer.

Minor comments
Page 3, last paragraph. Correction. The latter structures resembled lipid droplets, and to confirm this, we stained cells with the neutral lipid dye (LipidTOX) and lipid droplet marker (PLIN2).
For consistency, I will suggest that authors clearly state whether it is a stable expression or overexpression of the protein in the main figure legends. At some places, they state this, and sometimes it just says, "cells expressing."
Including page and line numbers in the submitted manuscript would facilitate the review process and make it easier for reviewers to provide specific comments.

Version 1:

Reviewer comments:

Reviewer #1

(Remarks to the Author)
The authors have addressed most of my concerns during the revision. The current working model is rather different from the previous model. However, given the existing evidence, such model is more plausible. The analysis with the TMs and lipid accumulation part is a bit weak. It is suggested that the authors strengthen this part in future studies. I still think the initial screen, even though served its purpose, is a bit out of context. It would be reasonable that the authors come back to this point in the following investigations. For example, is it possible to relocate the protein to Golgi (mutating the COPI site) and see if LD biogenesis is affected.

Reviewer #2

(Remarks to the Author)
I thank the authors for their thorough and considered responses to the reviewers' comments. The revised manuscript is substantially strengthened by the new quantitative colocalization analyses, the dose-response and knockdown-rescue experiments addressing overexpression artifacts, and the updated molecular dynamics simulations that have led the authors to revise their structural model of TMEM263 topology. I particularly commend the authors for tempering claims that were not fully supported by their data — notably the assertion of TMEM263-seipin colocalization once quantification and STED imaging showed minimal overlap, and appropriately softening the "LD-resident protein" claim to "ER-resident protein that colocalizes with LD markers."
That said, a few points still need to be clarified before I can fully endorse the revision. First, the authors describe "significant colocalization" between EGFP-TMEM263 and TGN46, but this doesn't match the Manders coefficients in Rebuttal Table 1, which are very low (M2 ≈ 0.02–0.07). It's also unclear what the authors are using as a true negative control here — the table as presented seems to show reproducibility between repeated images of the same sample, not a comparison to an unrelated marker. Please clarify this. Second, the Western blots showing similar expression levels of the TMEM263 mutants and truncations (addressing Comments 9 and 10) are a good start, but it would be stronger to quantify band intensity relative to the loading control, rather than relying on visual comparison of the blots alone. Finally, in Rebuttal Fig. 3, the statistical significance of the dose-response relationship between TMEM263 expression and LD size/number is inconsistent across comparisons. The manuscript text should reflect this same caution shown in the rebuttal, rather than implying a clear, consistent trend. These points are relatively minor and do not take away from what is otherwise a well-done and convincing revision.

1. Comment 3 (TGN46 colocalization claim): The manuscript states "significant colocalisation of EGFP-TMEM263 with Sec61β and TGN46," but the Manders M2 values reported in Rebuttal Table 1 for TMEM263–TGN46 are very low (0.02–0.065), inconsistent with a "significant" colocalization claim. Please reconcile the text with these values, or provide additional quantification that supports the stated conclusion.
2. Comment 3 (negative control): The reviewer requested a negative control consisting of two unrelated markers (e.g., ER vs. Golgi). Rebuttal Table 1 as presented shows reproducibility between two acquisitions of the same image (a positive/technical control), not a comparison between unrelated structures. Please clarify which dataset serves as the true negative control, or provide one if it is missing.
3. Comments 9 & 10 (mutant/truncation expression levels): Western blots are provided (Fig. S4B,F, S5D) showing expression of TMEM263 mutants and truncations, but expression levels are not quantified?. Please provide densitometric quantification (band intensity normalized to loading control, ideally with statistics across replicates) to substantiate the claim that expression levels are comparable to wild-type.
4. Comment 17 (dose-response statistics): In Rebuttal Fig. 3, statistical significance across the control/low/medium/high EGFP-TMEM263 dose comparisons is inconsistent (e.g., LD size is significant for control-vs-high and low-vs-high but not control-vs-medium; LD number does not reach significance at any comparison). Please ensure the manuscript text describes this relationship with the same caution reflected in the rebuttal, rather than implying a uniformly significant dose-dependent trend.

Reviewer #3

(Remarks to the Author)
The authors have adequately addressed my comments. Some of the questions are beyond the scope of this work, and I am satisfied with their rebuttals. I have no further comments and would recommend this work to be published in Nature Communications.

Open Access This Peer Review File is licensed under a Creative Commons Attribution 4.0 International License, which permits use, sharing, adaptation, distribution and reproduction in any medium or format, as long as you give appropriate credit to the original author(s) and the source, provide a link to the Creative Commons license, and indicate if changes were made.
In cases where reviewers are anonymous, credit should be given to 'Anonymous Referee' and the source.
The images or other third party material in this Peer Review File are included in the article's Creative Commons license, unless indicated otherwise in a credit line to the material. If material is not included in the article's Creative Commons license and your intended use is not permitted by statutory regulation or exceeds the permitted use, you will need to obtain permission directly from the copyright holder.
To view a copy of this license, visit https://creativecommons.org/licenses/by/4.0/

Point-by-point response to reviewers:

<u>Reviewer #1</u> (Remarks to the Author)

1) The introduction of TMEM263 is through a screen for COPI binding proteins, which basically selects potential ER and Golgi-resident proteins (note that COPI also works to retain Golgi-resident proteins). It is not logically fluent with the following focus on TMEM263 and LD biogenesis. It is suggested that this part to be tuned down and possibly moved to supplementary info.

We have modified the text to reduce focus here but feel it is important to demonstrate how we initially identified TMEM263. Furthermore, given there is published evidence of COPI involvement in lipid droplet biology (PMID: 23901109, PMID: 24497546), we think the fact that TMEM263 is a COPI cargo, could be of general interest to the field.

2) The authors propose that TMEM263 functions during lipid droplet (LD) biogenesis and may translocate from the ER to LDs. While LipidTOX is commonly used to label mature LDs, the authors could employ other probes or marker proteins (such as GFP-GPAT4-TM or GFP-DFCP1) to specifically label nascent LDs and examine whether TMEM263 localizes to these nascent structures. Furthermore, live-cell time-lapse imaging of GFP-tagged TMEM263 would be essential to directly observe its dynamic translocation from the ER to LDs.

We agree that establishing the relationship of EGFP-TMEM263 with nascent vs mature LDs is important for this manuscript. To address this, we have carried out live-cell imaging of HeLa cells expressing EGFP-TMEM263, the nascent LD marker, mCherry-HPos (PMID: 24368806) and LipidTOX 0-18 minutes after oleic acid addition ***(manuscript Fig. 2J, K)****. Consistent with the literature, mCherry-HPos labelling of puncta occurs prior to the acquisition of LipidTOX signal (PMID: 24368806). Our data reveal that EGFP-TMEM263 puncta start to emerge on average ~6.5 minutes after the addition of oleic acid. They are initially negative for HPos and LipidTOX. EGFP-TMEM263 puncta subsequently acquire mCherry-HPos signal ~8 minutes, and LipidTOX labelling ~10 minutes after feeding. These data indicate that EGFP-TMEM263 localises to both nascent LDs (mCherry-HPos^{+ve}, LipidTOX^{-ve}) and mature LDs (mCherry-HPos^{+ve}, LipidTOX^{+ve}).*

*We were unable to extend the live-cell time-lapse imaging for more than 20 minutes after the addition of oleic due to fluorophore bleaching. In this 20 minute timeframe, we were unable to observe any transition of EGFP-TMEM263 into the characteristic 'ring-like' distribution around a LipidTOX core. Anecdotally, all EGFP-TMEM263-puncta seemingly remained attached to the ER network (****also see Video 1****), and although our data demonstrate localisation with both nascent and mature LD markers, we cannot definitively say that it transitions from the ER to the LDs based on these experiments. As such, we have toned down this suggestion in the text, focusing instead on TMEM263 as an ER-resident protein.*

Please also see a similar comment made by Reviewer 3 (Comment 2)

3) The TMEM263 hairpin as predicted by multiple methods, is not a typical hairpin. The connecting loop of the two TM in the luminal side is simply too long and not feasible for monolayered phospholipids on the surface of LD. In fact, the simulation is done with bilayer. It contains multiple hydrophilic residues (K63, S64, E66, T68, K69, T70, T73/74, S77) between its two transmembrane domains, which would be energetically unfavorable for insertion into the hydrophobic core of LDs (Fig 4B shows lower lipid binding properties in this region). Unlike PLIN1 and UBXD8/FAF2 (which are not full transmembrane proteins) or GPAT4 (which has only a short base and features a V-shaped hydrophobic hairpin structure), how could TMEM263 insert into LD membrane remains unclear. An in vitro reconstitution assay using purified TMEM263 and artificial LDs could determine whether TMEM263 can incorporate into the LD phospholipid monolayer.

Initially, to address this important question, we tried modelling TMEM263 in a monolayer. The very high polarity of the inter-TM hinge however makes the conformation very unstable, and it very quickly partitions out of the membrane in the simulations. We chose not to report this in the manuscript, as the speed of the partitioning is too fast to be reliably interpreted, and was hard to support without extensive experimental analyses, such as the one suggested by the reviewer. It is also possible that interactions with another protein binding partner would ameliorate this effect. These experiments were however not feasible in this current study but remain important ongoing work. As mentioned above - we have modified the text to stress that our data place TMEM263 in the ER at sites of LD biogenesis.

Please also see a similar comment made by Reviewer 3 (Comment 4).

4) The authors did not detect the interaction between TMEM263 and seipin, and notably, TMEM263 forms 2-3 punctate structures on LD surfaces that are distinct from seipin's localization. More conclusive evidence is needed to clarify TMEM263's specific role in mediating ER-LD contact sites and facilitating LD biogenesis or growth.

We have included several new pieces of data to support the conclusion that TMEM263 plays a specific role in facilitating LD biogenesis. Firstly, and also please see answer to point 2, we have included live imaging which identified EGFP-TMEM263 at nascent LDs (mCherry-HPos^{+ve}, LipidTOX^{-ve}) ***(manuscript Fig. 2J,K)****. Secondly, we have also carried out more extensive analysis of LD size in cells expressing EGFP-TMEM263, demonstrating they are significantly larger than LD in control cells both in oleic acid-fed conditions supporting a role for TMEM263 in LD growth* ***(manuscript Fig. 2D,E and Fig. S2D,E)****. And thirdly, we have expanded our analysis of LDs in cells depleted of TMEM263 and show that upon oleic acid feeding, fewer LDs accumulate, further indicating a role for TMEM263 in LD biogenesis and growth* ***(manuscript Fig. 5A-D****). With regards to the ER-LD contact sites, this was postulated in the discussion as a potential role for TMEM263. This remains an open question, and to reduce any ambiguity, we have removed this from the discussion.*

5) Fig. 5F: BODIPY staining is less potent than LipidTOX; it is recommended to switch to LipidTOX for lipid droplet labeling to align with the cell experiments.

We have now tested LipidTOX staining in oleic acid-fed wildtype zebrafish larvae, at the same age as shown in ***manuscript Fig. 5G****. We found that LipidTOX shows comparable brightness to BODIPY but in some cases, greater background of the surrounding blood vessels, making imaging of LDs more challenging* ***(Rebuttal Fig. 1)****. In a review of the literature, across 18 papers looking at LDs in zebrafish larvae, the majority (10) use BODIPY, whilst six use Nile red/Oil red O and only 2 used LipidTOX. We would therefore suggest that using BODIPY for zebrafish studies is more appropriate, but we agree with the reviewer that it was important to confirm this by the staining with LipidTOX.*

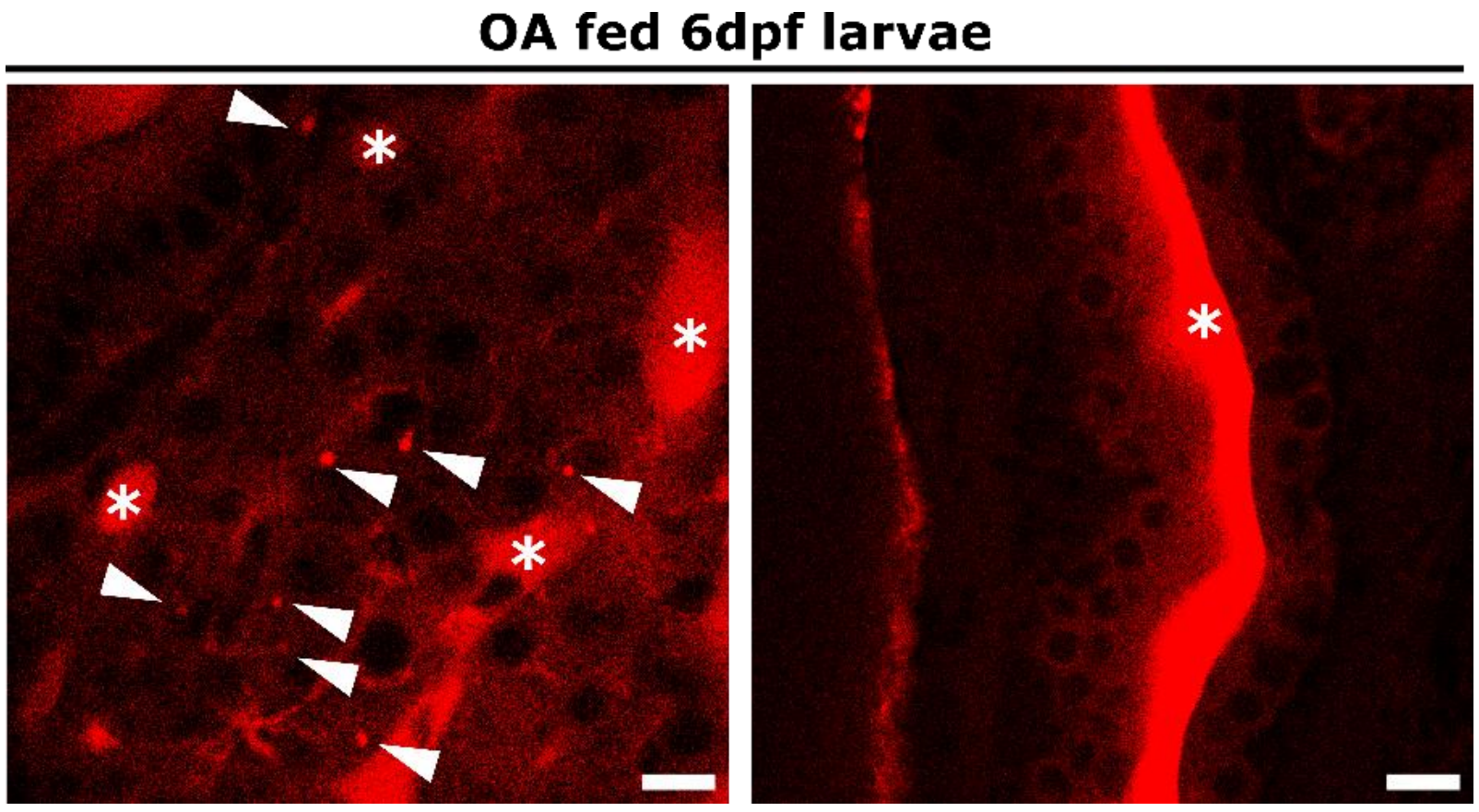

Rebuttal Figure 1- Confocal z-slices of *wild-type* zebrafish larvae at 6 days post-fertilisation (dpf) after feeding with oleic acid (OA) for 24hrs and staining with LipidTOX for 2hrs. Scale bars is 10 μm. White arrowheads show lipid droplets; white asterisks show blood vessels.

6) In Fig. 2I, the authors used an endogenous antibody to stain TMEM263. The specificity of the antibody needs to be demonstrated in the sup info, possibly a control staining using KO cells.

*This has now been included for the two antibodies we have available for TMEM263 (****Rebuttal Fig. 2A,B and manuscript Fig. S3J).*** *We have also included a validation of the antibodies by western blot (****Rebuttal Fig. 2C,D and manuscript Fig. S6A).***

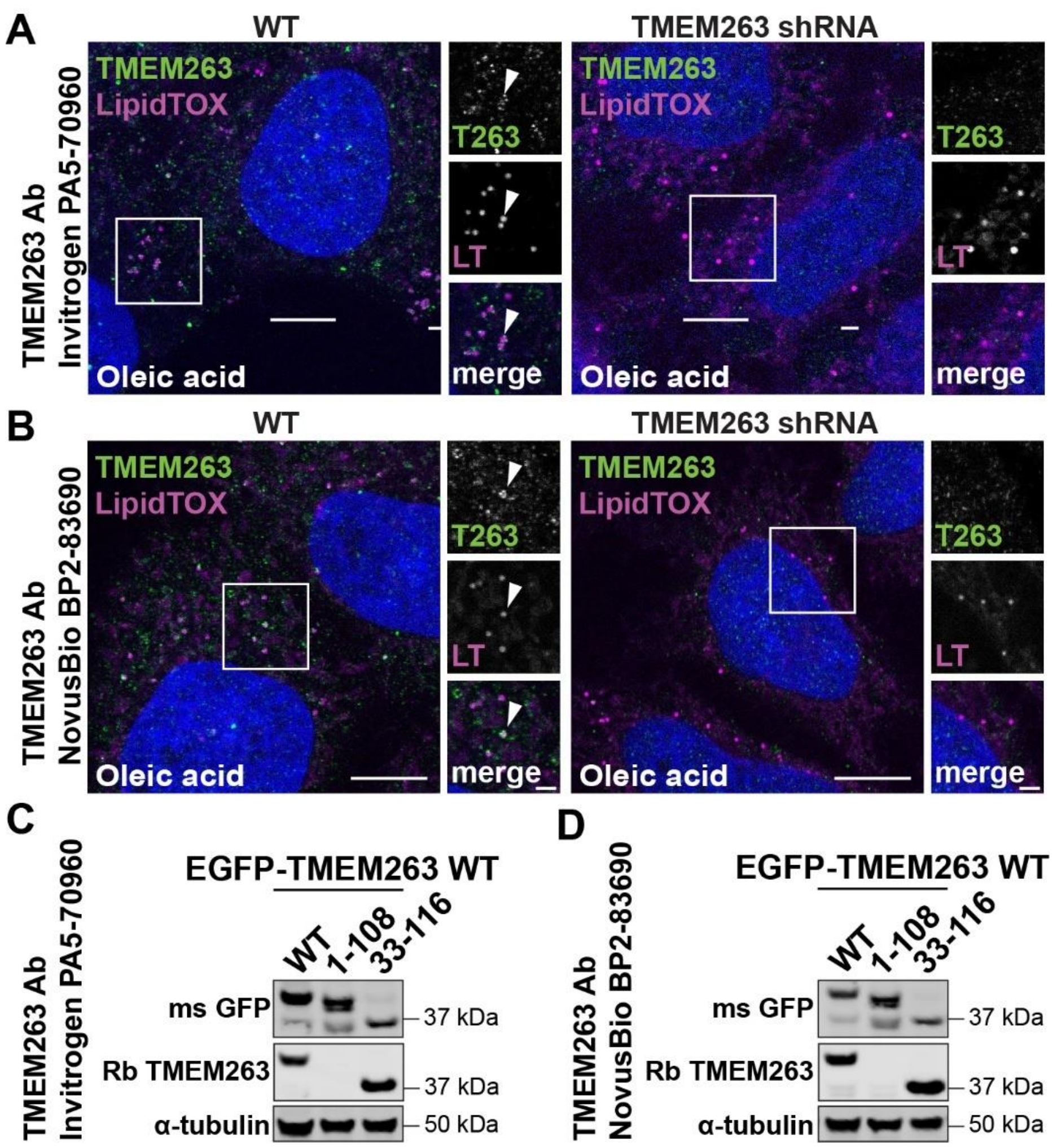

Rebuttal Figure 2: Validation of endogenous TMEM263 antibodies.

Reviewer #2 (Remarks to the Author):

Comments on the Manuscript

1. Whether that tag used with TMEM263 is GFP or EGFP? If its EGFP the name should be changed accordingly for all chimeric proteins.

All text and figures have been modified to specify EGFP-TMEM263.

2. The statement “When the neutral lipid accumulation surpasses a critical concentration (~5–10 mol%), they undergo phase separation into a lipid ‘lens’” requires a supporting reference for the critical concentration threshold. Please insert an appropriate citation.

Four additional citations have been added to the manuscript (PMID: 23567913, PMID: 20877640, PMID: 33293168 and PMID: 6947263) to include in vitro and computational studies.

Please also see response to Reviewer 2 Comment 11.

3. The claim of colocalization of GFP-TMEM263 with Sec61 or TGN46 in Figure 1 would be strengthened by quantitative analysis (e.g., pairwise cross-correlation). Appropriate negative controls (two unrelated images, e.g., ER vs. Golgi) and positive controls (two acquisitions of the same GFP-TMEM263 image) should also be included.

*Co-localisation analysis of EGFP-TMEM263 with Sec61β and TGN46 (plus negative control, ER vs Golgi) is now included (**manuscript Fig. S1F)** and shows significant colocalisation of EGFP-TMEM263 with Sec61β and TGN46, consistent with it being a COPI cargo. Positive controls (two acquisitions of the same EGFP-TMEM263 image) are included in **Rebuttal Table 1**.*

Type of co-localisation	Image & cell analysed	Manders coefficient (M2) First acquisition	Manders coefficient (M2) Second acquisition	Difference (%)
**EGFP-TMEM263 & mCherry-Sec61β *live imaging**	Image 1, cell 1	0.728	0.770	5.77
	Image 1, cell 2	0.724	0.773	6.77
**EGFP-TMEM263 & TGN46 *fixed samples**	Image 1, cell 1	0.063	0.065	3.17
	Image 1, cell 2	0.051	0.055	7.84
	Image 2, cell 1	0.045	0.044	-2.22
	Image 2, cell 2	0.020	0.019	-5.00
	Image 2, cell 3	0.036	0.035	-2.78
**TGN46 & Sec61β *fixed samples**	Image 1, cell 1	0.645	0.635	-1.55
	Image 1, cell 2	0.709	0.685	-3.39
	Image 2, cell 1	0.563	0.554	-1.60
	Image 2, cell 2	0.455	0.436	-4.18
	Image 2, cell 3	0.742	0.739	-0.40

Table 1: Positive controls (two acquisitions of the same image) for co-localisation EGFP-TMEM263 / ER / Golgi.

4. The fraction of cells exhibiting the “bunch-of-grapes” phenotype in both control and GFP-TMEM263-expressing cells should be quantified. In addition, this phenotype is not observed in MEFs and RPE-1 cells, which should be noted in the Results section.

*We apologise if it is not clear- the ‘bunch-of-grapes’ phenotype is quantified, as ‘clustered’ in **manuscript Fig. 2F** for HeLa and **manuscript Fig. S3F,I** for MEFs and RPE-1s. The data show that overexpression of EGFP-TMEM263 in oleic acid-fed conditions is associated with an increase LD clustering in all three cell lines.*

5. In several places, GFP-TMEM263 is incorrectly referred to as TMEM263. This may cause confusion with the endogenous protein. For example, the sentence “TMEM263-positive lipid

droplets further confirms TMEM263 is recruited to lipid droplet membranes (Fig. S2E)” should be corrected to specify GFP-TMEM263.

We have modified the text to ensure it is clear when TMEM263 has been overexpressed.

6. While colocalization of GFP-TMEM263 with multiple LD-resident proteins and LipidTOX has been shown, the conclusion that TMEM263 is definitively an LD-resident protein is premature. Either this should be stated more cautiously, or higher-resolution imaging (e.g., TEM or SEM) could be used to confirm localization.

We agree with the reviewer on this point, and we have now modified the text to clarify that our data identify TMEM263 as an ER resident protein that colocalises with nascent and mature LD markers, we cannot definitively say at this point that it is a LD-resident protein.

7. In Figure S1B, a higher-resolution, zoomed-in image should be provided to clearly demonstrate colocalization of GFP-esp-COP1 with the ER and Golgi. It would be clearer to present these as pairwise comparisons (e.g., EGFP-esp-COP1 with ER [red/green pair] and EGFP-esp-COP1 with Golgi [red/green pair]).

***Manuscript Fig. S1B** has now been modified to include higher-resolution, zoomed-in images and pairwise comparisons of GFP-epsCOPI with Golgi (TGN46) and GFP-epsCOPI with ER (Sec61β).*

8. In Figure 4G, the corresponding immunofluorescence images of mutant expression should be included.

*The images corresponding to mutant expression are included in **manuscript Fig. 4E and Fig. S5E**, and western blotting of expression levels are shown in **manuscript Fig. S5D**.*

9. Regarding the statement “mutant GFP-TMEM263 positive puncta was significantly reduced compared to wild-type,” the authors should first demonstrate that expression levels of each mutant are comparable to wild-type.
10. Similarly, in Figure 4G, expression levels of all mutants should be shown to be comparable.

*Blots showing the expression level of EGFP-TMEM263 truncations and point mutants are now included in **manuscript Fig. S4B,F and Fig. S5D**, respectively.*

11. Is there experimental evidence that 2.5% TAG concentration is indeed below the threshold for spontaneous lipid coalescence? Please clarify.

The critical concentration of TAG above which phase nucleation starts is dependent on multiple factors such as the exact lipid composition used, the surface tension of the bilayer, the elasticity of the membrane (angle potential parameter k_BT), and temperature. This critical

concentration has been measured experimentally to be at ~2.8% (Hamilton, Small 1981 – PMID: 6947263). Other reports estimate that this critical concentration is 3-5% depending on parameters used (Thiam, Ikonen 2021 – PMID: 33293168). Studies using similar membrane composition and the same software report a critical concentration of 3% (Khandelia et al 2010 – PMID: 20877640). To verify that 2.5% is below the critical concentration of the system used in our modelling, we performed the simulation at 2.5% TAG and with no protein present, which demonstrated that the lipids do not spontaneously coalesce, and this is quantified across 20 repeats ***(manuscript Fig. 4C,D)****.*

12. The image quality in Figure 1C needs improvement to convincingly demonstrate colocalization. Pairwise presentation would enhance clarity.

We have replaced ***manuscript Fig. 1C*** *with a selected frame from live-cell imaging using a spinning disk microscope. The reticular localisation of EGFP-TMEM263 shows clear colocalisation with the ER-marker, Sec61β. Pairwise presentation of EGFP-TMEM263 with Sec61β and of EGFP-TMEM263 with TGN46 (trans-Golgi) is also presented in a new image in* ***manuscript Fig. S1E****.*

13. In the sentence “As expected, GFP-TMEM263 colocalised with the ER, Golgi (Fig. 1C) and endogenous COPI (Fig. 1D), the latter at small vesicular structures and at a region consistent with the Golgi,” the vesicular structures should be indicated in the figure with arrows.

Arrows/arrowheads have now been added to ***manuscript Fig. 1C*** *and* ***Fig. S1E,G,H****.*

14. From Figures 2I and 2J, it cannot be conclusively stated that endogenous TMEM263 is LD-resident. This should be more cautiously discussed in the manuscript.

We have modified the text to more cautiously discuss the observed localisation of endogenous TMEM263 in the vicinity of LDs in ***manuscript Fig. 2H,I****.*

15. The localization pattern of GFP-TMEM263 (Figures 2B, 2C, 2G, 2H) appears different from that of endogenous TMEM263 (Figures 2I, 4A). Could the GFP tag itself be affecting localization? This should be addressed.

The reviewer raises an important point here. We have now included data demonstrating that overexpression of FLAG-HA-TMEM263 (a significantly smaller tag) also induces an accumulation of LipidTOX^{+ve} LDs ***(manuscript Fig. 2G)****. The FLAG signal can be seen to surround the neutral lipid core similar to EGFP-TMEM263. We also see endogenous TMEM263 localising at the edge of LipidTOX LD core, and occasionally, this distribution encircles the LD, similar to the overexpressed constructs* ***(manuscript Fig. 2H, example 3)****. The low levels of endogenous TMEM263 protein in the cell and the quality of available reagents are likely to contribute to the increased variability in signal and distribution seen compared to the overexpressed constructs.*

16. The mechanism by which proteins containing transmembrane domains localize to LDs should be discussed in the manuscript.

Our new MD simulations provide more conclusive evidence that TMEM263 contains two transmembrane domains connected with a linker, and it does not exist as the hairpin we originally postulated it might. As such, we have modified our hypothesis that TMEM263 would be able to transition to a lipid droplet monolayer and we have instead focused on TMEM263 as an ER-resident protein.

17. The claim that TMEM263-positive LDs are larger than controls (Figure 2) is not sufficiently supported. Representative images alone are inadequate. A quantitative analysis of LD size distribution should be performed in at least 15–20 randomly selected control cells. Similar analyses should be carried out in GFP-TMEM263–expressing cells at varying expression levels (high, medium, low; controlled by transfection DNA amounts). Expression levels of GFP-TMEM263 should also be quantified. Intra-cellular and inter-cellular variations of LD sizes should be quantified.

*We have now performed quantitative analysis of LD size distribution in oleic acid-fed control and EGFP-TMEM263 expressing cells **(manuscript Fig. 2D,E)**. Furthermore, we have included histograms to demonstrate intra-cellular and inter-cellular variations of LD size in the same conditions **(manuscript Fig. S2D,E)**. These analyses demonstrate that EGFP-TMEM263 expression promotes increased LD size (from mean of 0.15 μm^2 in controls to 0.28 μm^2).*

*As requested by the reviewer, we have carried out an additional experiment to quantify LD size in cells expressing increasing amounts of EGFP-TMEM263 in steady-state (i.e. not oleic acid fed) conditions **(Rebuttal Fig. 3)**. These data demonstrate LD size, and number correlates with TMEM263 expression levels, although the latter is not statistically significant.*

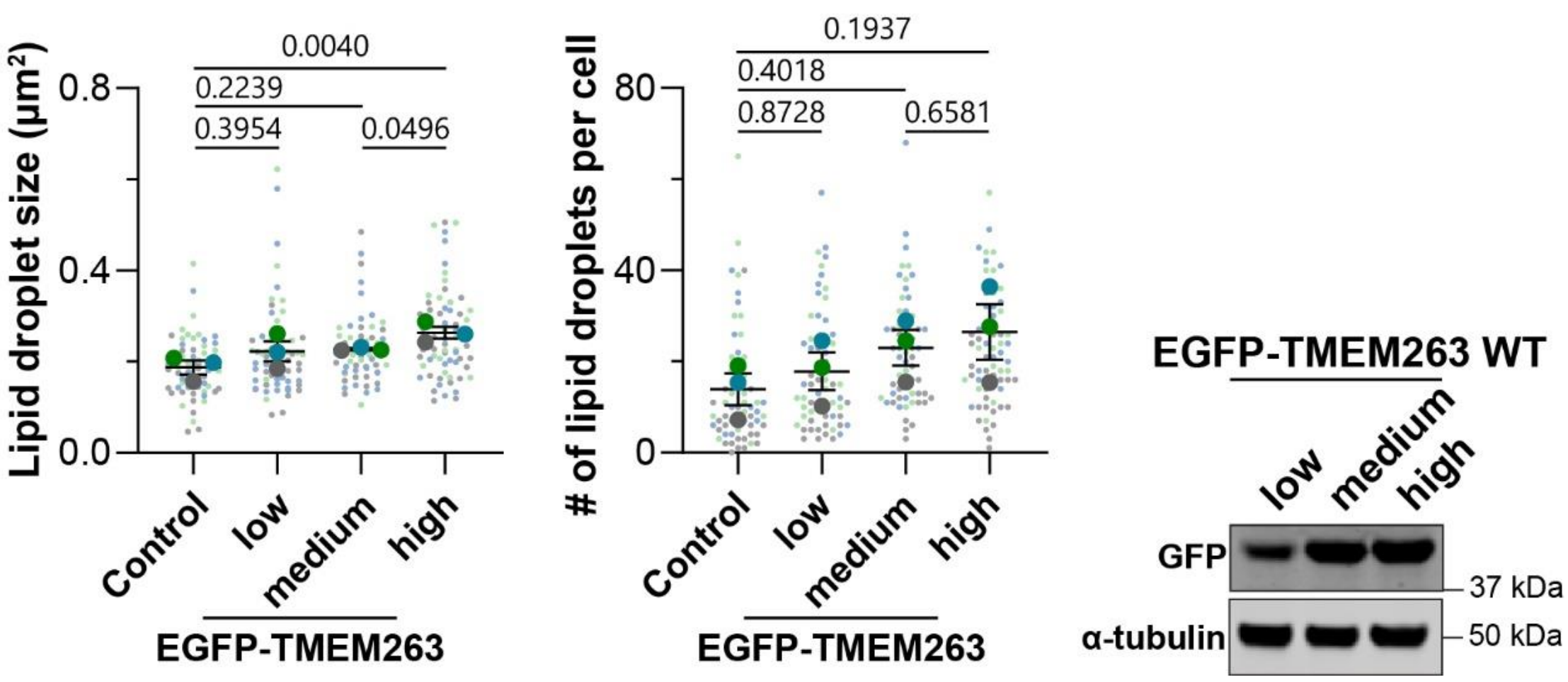

***Rebuttal Figure 3:** HeLa cells were transfected with increasing amounts of EGFP-TMEM263 DNA (low: 0.1 µg/well, medium: 0.2 µg/well, high: 0.25 µg/well in 24 well plates) Nb. 0.2 µg/well was used in all experiments in the main study). Cells were fixed 24h post-transfection and*

stained with LipidTOX. Lipid droplet size and number were quantified. Representative blot showing the expression levels of EGFP-TMEM263.

18. To confirm that GFP-TMEM263 localization to LDs is not an artifact of overexpression, the authors should examine GFP-TMEM263 localization after endogenous TMEM263 knockdown. This would help establish whether GFP tagging alters localization.

This is now included in ***manuscript Fig. S3K****. We can confirm that EGFP-TMEM263 overexpression in cells treated with shRNA against the UTR of TMEM263 (so it does not target overexpressed TMEM263) localises to LipidTOX*$^{+ve}$ *LDs.*

19. The inference of colocalization between endogenous TMEM263 and seipin/BSCL2 (Figure 4A) is not sufficiently supported by the presented image. Quantitative colocalization analysis is needed. A negative control (e.g., TMEM263 with a protein forming puncta but unrelated to LDs, such as TNFR1) should also be included.

We apologise for any confusion; we did not mean to suggest that there was significant co-localisation between TMEM263 and seipin as our own biochemistry (data not shown) and IF data do not suggest any interaction. To further clarify this point, we have 1) quantified EGFP-TMEM263- seipin colocalisation, alongside a negative control, EGFP-TMEM263- p62 ***(Rebuttal Fig. 4 and manuscript Fig. S3L,M)****; 2) carried out higher resolution imaging of endogenous TMEM263, endogenous seipin and BODIPY using STED microscopy* ***(manuscript Fig. S3N)****. At this higher resolution, we confirm that there is very little co-localisation between endogenous TMEM263 and Seipin.*

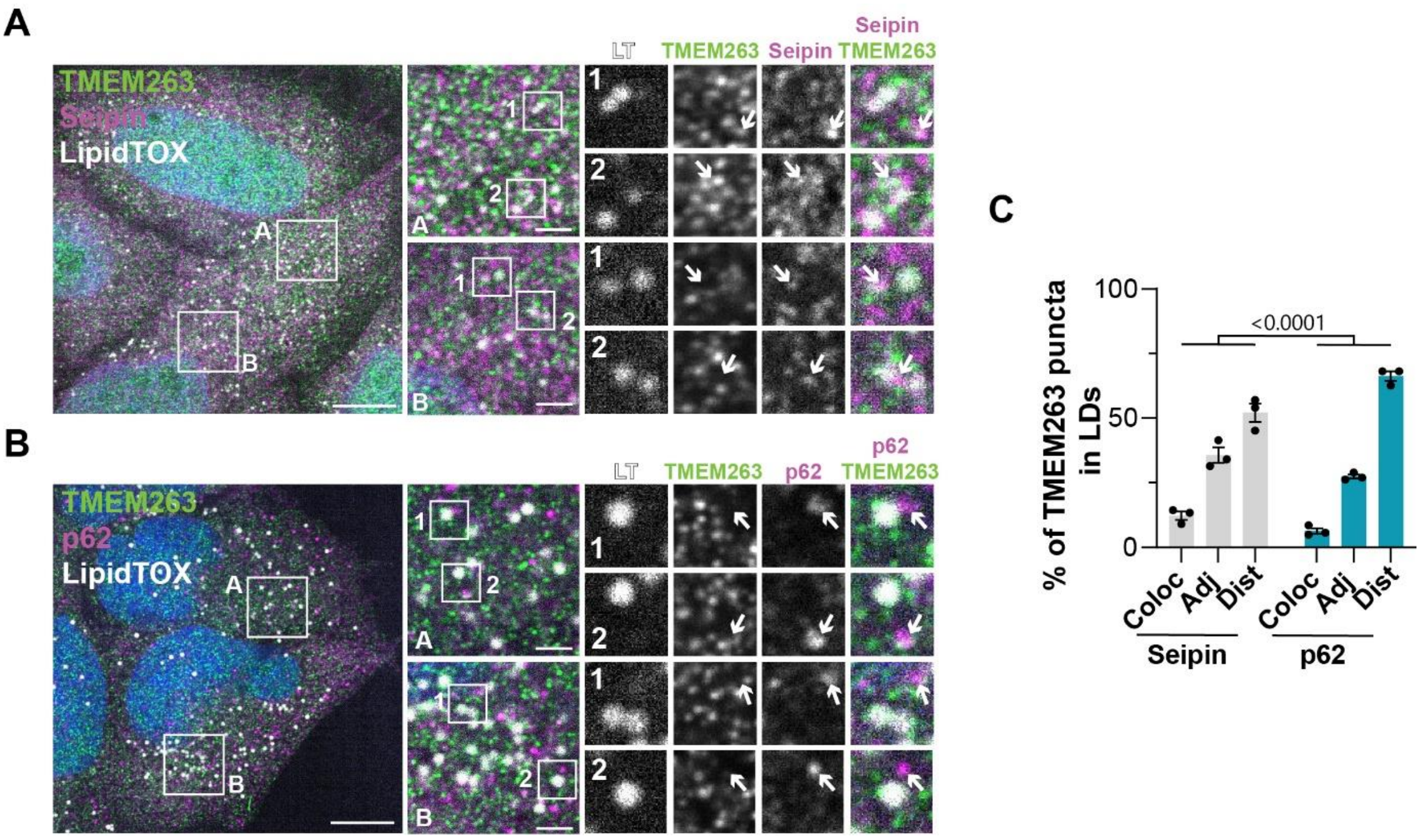

Rebuttal Figure 4: *Quantification of distance between TMEM263 and Seipin or p62 puncta associated with the same lipid droplet and divided in the following categories: colocalisation (Coloc), adjancent (Adj) and distinct (Dist).*

20. In Figure 5, TMEM263 knockdown in HeLa cells appears to reduce LD size rather than number. Since quantification of LD number may be unreliable when LD size decreases, a more rigorous analysis is required. Higher-magnification imaging and 3D imaging should be performed to quantify both LD number and size accurately.

More extensive analysis of LDs in HeLa cells depleted of TMEM263 have now been carried out i.e. at higher magnification and with confocal z-stacks ***(manuscript Fig. 5A-D****). Loss of TMEM263 leads to reduced number of LDs and reduced % LipidTOX-positive area. Our analysis indicates there is no significant difference in LD size in TMEM263-depleted cells.*

21. The zebrafish blastodisc contains LDs (see: https://pubmed.ncbi.nlm.nih.gov/26355567). It would be valuable to analyze LD size and number distribution in tmem263 crispants at the 1- and 2-cell stages.

We appreciate the reviewer's comment and believe there is a lot of exciting biology to be done in our follow-up studies in vivo. As such, we have not carried out this specific experiment, and are focusing on establishing a stable, whole-body knock-out of tmem263. This will allow us to comprehensively investigate the role and regulation of tmem263 in vivo in our on-going work.

Reviewer #3 (Remarks to the Author)

Major Comments:

Comment 1: In Figure 3C, the author stated that the overexpression of protein truncations of GFP-TMEM263 in HeLa cells is required for proper localization to the ER and induces the accumulation of TMEM263-positive LDs. However, in the gray-scale images, it is hard to visualize the accumulation of TMEM263-positive LDs. Also, the LD clustering/accumulation phenotype appears to be relatively weak.

We have replaced the images in Fig. 3C to improve the resolution, and they now include co-labelling with the ER-marker, Sec61β. The lack of robust clustering in this figure is because these images are at steady-state rather than in oleic acid-fed conditions, which is the equivalent to Fig. 2A.

Comment 2: Based on the limited co-localization of endogenous TMEM263 with seipin on a subset of LDs in Figure 4A, the suggestion that TMEM263 functions in the early stages of LD formation implied in the manuscript title requires further experimental support. I will suggest employing live-cell imaging using early LD biogenesis markers such as the model peptide HPos, LiveDrop, or acyl-CoA synthetase 3, which is known to localize to nascent LD assembly sites and is essential for efficient LD nucleation (PMID: 24368806)

Please also see response to Reviewer 1 Comment 2. Using live-cell imaging of EGFP-TMEM263, mCherry-HPos and LipidTOX, we can reveal that EGFP-TMEM263 puncta

*emerge first and sequentially become positive for mCherry-HPos and then LipidTOX. This data is quantified and has been included in **manuscript Fig. 2J,K**. These data support a role for TMEM263 early in LD biogenesis.*

Comment 3: While AlphaFold has transformed structure prediction, its application to membrane proteins remains limited, especially when lipid-dependent conformational changes are involved. The static nature of AlphaFold's predictions does not account for dynamic structural rearrangements driven by membrane composition or lipid-protein interactions. Therefore, in Figure 4B, the predicted hairpin structure needs to be experimentally verified using biochemical and biophysical experiments, as done elegantly in this study (PMID: 38802378), to eliminate the possibility that the protein does not associate with the ER via an amphipathic helix and indeed adopts a hairpin topology. Additionally, the authors should display helical wheel projection for each hairpin in the figure.

*The analysis carried out in PMID: 38802378 on UBXD8 is beyond the scope of this current study, but we have carried out additional modelling of TMEM263 in a phospholipid bilayer (**manuscript Fig. 3H)**. Our data suggest that TMEM263 has a TM helix (TM1), followed by a relatively long linker comprising both a helix (49-68) and a loop (69-79), and another TM helix (TM2). The helical wheel projection of these helices is also included in **manuscript Fig. S4H**. Furthermore, overexpression of individual putative TM domains (i.e. 1-60 and 60-116) show cytoplasmic distribution and no co-localisation with the ER (Sec61β) **(Fig. 3C)**, indicating they do not stably associate with membranes.*

Comment 4: The authors conducted relatively short 150 ns simulations to investigate TMEM263-neutral lipid/bilayer interactions. Given that the peptide structure was predicted using AlphaFold and considering the slow diffusion rates of lipids, the simulation timeframe provides insufficient sampling, even with five replicate simulations. The limited diffusion of lipids within this timeframe raises concerns about the statistical significance of the observed interactions. Furthermore, the authors did not experimentally validate the conformation of the peptide in the lipid membrane. Snapshots at different time points clearly demonstrate that the peptide conformation has not reached equilibrium or stabilized within the simulation period. The use of shorter acyl tails in DLPC/PE lipids to represent the thinner ER membrane further raises questions about the validity of the observed peptide structure and conformation in the bilayer.

There is a discrepancy in the methods section where the authors claim to have simulated up to 150 ns; however, the final structure shown in Figure S4, D is at 94.4 ns. It is not clear what happened to the peptide structure between 94-150 ns. This raises concerns about the stability and reliability of the reported conformations. The use of AlphaFold-predicted structures with short simulations (100-150 ns) is insufficient for drawing reliable conclusions about TMEM263-membrane interactions. Previous studies on UBXD8 (PMID: 38802378) employed more extensive MD simulations at the microsecond timescale, complemented by solid experimental data to validate the structure in the bilayer and monolayer. Authors should extend simulations to the microsecond timescale to provide meaningful structural insights into TMEM263-membrane interactions and include experimental data to support the computational findings.

The reviewer is justified in requesting more sampling and analysis here. We have now performed new atomistic simulations, both in a larger box (from 8 x 8 x 8.5 nm to 11 x 11 x 10 nm) and with considerably more sampling, with five repeats each lasting 1 μs. We have included a number of analysis methods to better characterise the behaviour of the protein and assess convergence ***(manuscript Fig. 3I-K, Fig. S4G,I-J, Fig. 4A,B, and Fig. S5A)****. Our data provide a clear overview of the structural dynamics of TMEM263, that adopts a stable, yet dynamical, alpha hairpin.*

We have also linked our MD observations to mutagenesis experiments to provide some further insights to the findings. Point mutations to F43, W59, and K84 residues of TMEM263 and their effect in localisation (loss of ER localisation in W59A – ***manuscript Fig. 4E****) and lipid binding abilities (fewer lipid droplets and less clustering compared to TMEM263 WT –* ***manuscript Fig. 4F,G and Fig. S5F,E****) is supported by the high lipid occupancy of F43 (highest in TM1 –* ***manuscript Fig. S5A****), the position of bulky W59 at the start of the inter-TM linker* ***(manuscript Fig. 3H)****, and the role of K84 in the interaction of the bottom part of TM2 with the lipid headgroup* ***(manuscript Fig. 3H,J)****.*

The authors claim that peptides maintained the same orientation and remained evenly spaced around the lens with no evidence of oligomerization. However, several methodological concerns need to be addressed: In the context of LD biogenesis, where seipin is a key regulator, the authors should clarify the biological relevance of evenly spaced peptides around the lens. It is not surprising that peptides maintain their orientation in coarse-grained Martini simulations, as these simulations employ an elastic network that inherently restricts conformational changes in proteins. Martini 2 is known to produce artificially strong protein-protein interactions ("sticky" proteins). The article does not clarify whether: (a) Protein-protein interactions were scaled to reduce artificial aggregation; and (b) If peptide interactions with the lens reduce diffusion and artificially restrict aggregation. The authors should provide more detailed structural insights to support their claims about peptide behavior and arrangement. In addition, authors consider using Go-Martini and other Martini models to follow the conformational changes. These methodological considerations are crucial for properly interpreting the observed peptide behavior and its relevance to LD biogenesis mechanisms.

Given the caveats in interpreting the even distribution of TMEM263 around the lipid lens in CG simulations, we have modified the text, to more simply highlight that upon lens formation, TMEM263 localises to the edge. The model system, which also lacks seipin and other factors related to early LD formation, was designed to provide additional information about the behaviour of TMEM263 in a membrane environment

We note that while Martini 2.2 has been known to promote "sticky" proteins, we generally do not see TMEM263 interacting with itself in our simulations, so this does not appear to be an issue here. Furthermore, our biochemistry shows no indication that TMEM263 interacts with itself in cellulo so the model faithfully recapitulates this ***(manuscript Fig. S5C)****. We have trialled other Martini models (Go-martini, Martini 3), but ultimately, we do not consider these methods to be as reliable for modelling the relatively high dynamics of the TMEM263 protein as our atomistic MD. We would like to emphasise here that the goal of the CG MD was to model protein-lipid interactions, which are typically modelled very well in Martini 2.2.*

Another major limitation of this study is the absence of simulations in a LD-like monolayer environment. TMEM263 is proposed to localize to or interact with LDs, which are characterized by a phospholipid monolayer rather than a bilayer. Simulating the protein only within a bilayer membrane environment fails to recapitulate the biophysical constraints and properties like lipid packing and surface tension unique to LDs. This oversight may mask conformational changes or membrane-embedding modes that are specific to TMEM263 LD localization. Therefore, incorporating simulations in a lipid monolayer environment would be essential to better understand the structural dynamics and topological behavior of TMEM263 in its physiological context.

We had tried modelling TMEM263 in a monolayer, however the very high polarity of the inter-TM hinge (including several lysine, serine and threonine residues) makes the conformation very unstable, and very quickly partitions out of the membrane. We note that this would be an inevitable part of any TMEM263 model, and not simply a limitation of our input AF2 model. We chose not to report this in the manuscript, as the speed of the partitioning is too fast to be reliably interpreted, and was hard to support without extensive biophysical analyses, which are beyond the scope of this study. It is also possible that interactions with another protein binding partner would ameliorate this effect, a line of enquiry that we are actively pursuing for a follow up study.

Comment 5: The protein, as shown in Figure 4C and Figure S4 (A), accumulates near the LD rim and never transitions to the LD. Considering the high contact of neutral lipids to amino acids W59 and K84 (Figure 4E), does this interaction facilitate the switch in conformation of the TMEM263 from its deeply buried state within the bilayer to help it transition to the LDs? Due to the absence of this critical data, their MD simulations are just re-validating the earlier work (PMID: 32730754 (Olarte 2020); PMID: 38802378 (Dhiman 2024); PMID: 35583926; PMID: 37889293), and not providing any new structural insights. The current gap in knowledge is in identifying the factors that drive the partitioning of hairpin proteins from the bilayer to the monolayer.

In this study we focussed on what we could convincingly and reliably model and as detailed in the comment above, we were unable to reliably model TMEM263 in a monolayer. Our data suggest that TMEM263 is not a hairpin protein like the ones in the study mentioned by the reviewer and thus we do not consider TMEM263 a useful system to identify pathways from bilayer to monolayer. We agree with the reviewer that there is a gap in knowledge in identifying the factors that drive the partitioning of hairpin proteins from the bilayer to the monolayer, however this work is not trying to answer this particular question. We employ MD as an addition to our in cellulo and in vivo work to provide insights in the characterisation of a novel protein which appears to have an important role in LD biogenesis, and we are remaining open-minded about both structural and functional features of this.

Minor comments

Page 3, last paragraph. Correction. The latter structures resembled lipid droplets, and to confirm this, we stained cells with the neutral lipid dye (LipidTOX) and lipid droplet marker (PLIN2).

This has been modified.

For consistency, I will suggest that authors clearly state whether it is a stable expression or overexpression of the protein in the main figure legends. At some places, they state this, and sometimes it just says, "cells expressing."

The text has been modified to make it clearer where over-expression has been used.

Including page and line numbers in the submitted manuscript would facilitate the review process and make it easier for reviewers to provide specific comments.

This has now been done.

Point-by-point response to Reviewer 2:

I thank the authors for their thorough and considered responses to the reviewers' comments. The revised manuscript is substantially strengthened by the new quantitative colocalization analyses, the dose-response and knockdown-rescue experiments addressing overexpression artifacts, and the updated molecular dynamics simulations that have led the authors to revise their structural model of TMEM263 topology. I particularly commend the authors for tempering claims that were not fully supported by their data — notably the assertion of TMEM263-seipin colocalization once quantification and STED imaging showed minimal overlap, and appropriately softening the "LD-resident protein" claim to "ER-resident protein that colocalizes with LD markers."

That said, a few points still need to be clarified before I can fully endorse the revision. First, the authors describe "significant colocalization" between EGFP-TMEM263 and TGN46, but this doesn't match the Manders coefficients in Rebuttal Table 1, which are very low (M2 ≈ 0.02–0.07). It's also unclear what the authors are using as a true negative control here — the table as presented seems to show reproducibility between repeated images of the same sample, not a comparison to an unrelated marker. Please clarify this. Second, the Western blots showing similar expression levels of the TMEM263 mutants and truncations (addressing Comments 9 and 10) are a good start, but it would be stronger to quantify band intensity relative to the loading control, rather than relying on visual comparison of the blots alone. Finally, in Rebuttal Fig. 3, the statistical significance of the dose-response relationship between TMEM263 expression and LD size/number is inconsistent across comparisons. The manuscript text should reflect this same caution shown in the rebuttal, rather than implying a clear, consistent trend. These points are relatively minor and do not take away from what is otherwise a well-done and convincing revision.

1. Comment 3 (TGN46 colocalization claim): The manuscript states "significant colocalisation of EGFP-TMEM263 with Sec61β and TGN46," but the Manders M2 values reported in Rebuttal Table 1 for TMEM263–TGN46 are very low (0.02–0.065), inconsistent with a "significant" colocalization claim. Please reconcile the text with these values, or provide additional quantification that supports the stated conclusion.

2. Comment 3 (negative control): The reviewer requested a negative control consisting of two unrelated markers (e.g., ER vs. Golgi). Rebuttal Table 1 as presented shows reproducibility between two acquisitions of the same image (a positive/technical control), not a comparison between unrelated structures. Please clarify which dataset serves as the true negative control, or provide one if it is missing.

We apologise for the confusion. There was a labelling mistake in the table in the previous point-to-point response, the data that correspond to TGN46 & Sec61β (negative control) were labeled as EGFP-TMEM263 & TGN46 and vise versa. The table only shows the positive controls that the reviewer asked, which are quantifications two separate acquisitions of the same image. ***Table 1*** *now has been added below with the correct labelling. The full data that are plotted in* ***manuscript Fig. S1F*** *are included in the source data uploaded with both resubmissions. The mean values of each experiment are shown in the* ***Table 2****. The co-localisation analysis is based on images as shown in* ***manuscript Fig. 1C*** *(EGFP-TMEM263 & mCherry-Sec61β co-localisation) and* ***Fig. S1E*** *(TGN46 & Sec61β and EGFP-TMEM263 & TGN46 co-localisation). Therefore, EGFP-TMEM263 has a significant co-localisation with*

TGN46 (M2 > 0.7) and the co-localisation of TGN46 (Golgi) with Sec61β (ER) serves as the negative control (M2 < 0.15) that the reviewer requested.

**Type of co-localisation**	**Image & cell analysed**	**Manders coefficient (M2) First acquisition**	**Manders coefficient (M2) Second acquisition**	**Difference (%)**
**EGFP-TMEM263 & mCherry-Sec61β *live imaging**	Image 1, cell 1	0.728	0.770	5.77
	Image 1, cell 2	0.724	0.773	6.77
**TGN46 & Sec61β *fixed samples**	Image 1, cell 1	0.063	0.065	3.17
	Image 1, cell 2	0.051	0.055	7.84
	Image 2, cell 1	0.045	0.044	-2.22
	Image 2, cell 2	0.020	0.019	-5.00
	Image 2, cell 3	0.036	0.035	-2.78
**EGFP-TMEM263 & TGN46 *fixed samples**	Image 1, cell 1	0.645	0.635	-1.55
	Image 1, cell 2	0.709	0.685	-3.39
	Image 2, cell 1	0.563	0.554	-1.60
	Image 2, cell 2	0.455	0.436	-4.18
	Image 2, cell 3	0.742	0.739	-0.40

Table 1: Positive controls (two acquisitions of the same image) for co-localisation EGFP-TMEM263 / ER / Golgi.

**Type of co-localisation**	**Manders coefficient (M2)**		
	**n = 1**	**n = 2**	**n = 3**
**EGFP-TMEM263 & mCherry-Sec61β**	0.6481	0.6981	0.6328
**TGN46 & Sec61β**	0.1429	0.07733	0.05264
**EGFP-TMEM263 & TGN46**	0.7059	0.7671	0.7784

Table 2: Mean values of quantification of co-localisation EGFP-TMEM263 / ER / Golgi as presented in manuscript Fig. S1F.

3. Comments 9 & 10 (mutant/truncation expression levels): Western blots are provided (Fig. S4B,F, S5D) showing expression of TMEM263 mutants and truncations, but expression levels are not quantified?. Please provide densitometric quantification (band intensity normalized to loading control, ideally with statistics across replicates) to substantiate the claim that expression levels are comparable to wild-type.

*As requested by the reviewer, we have quantified the western blots shown in **manuscript Fig. S4B, F and Fig. S5D** in Rebuttal Figure 1.*

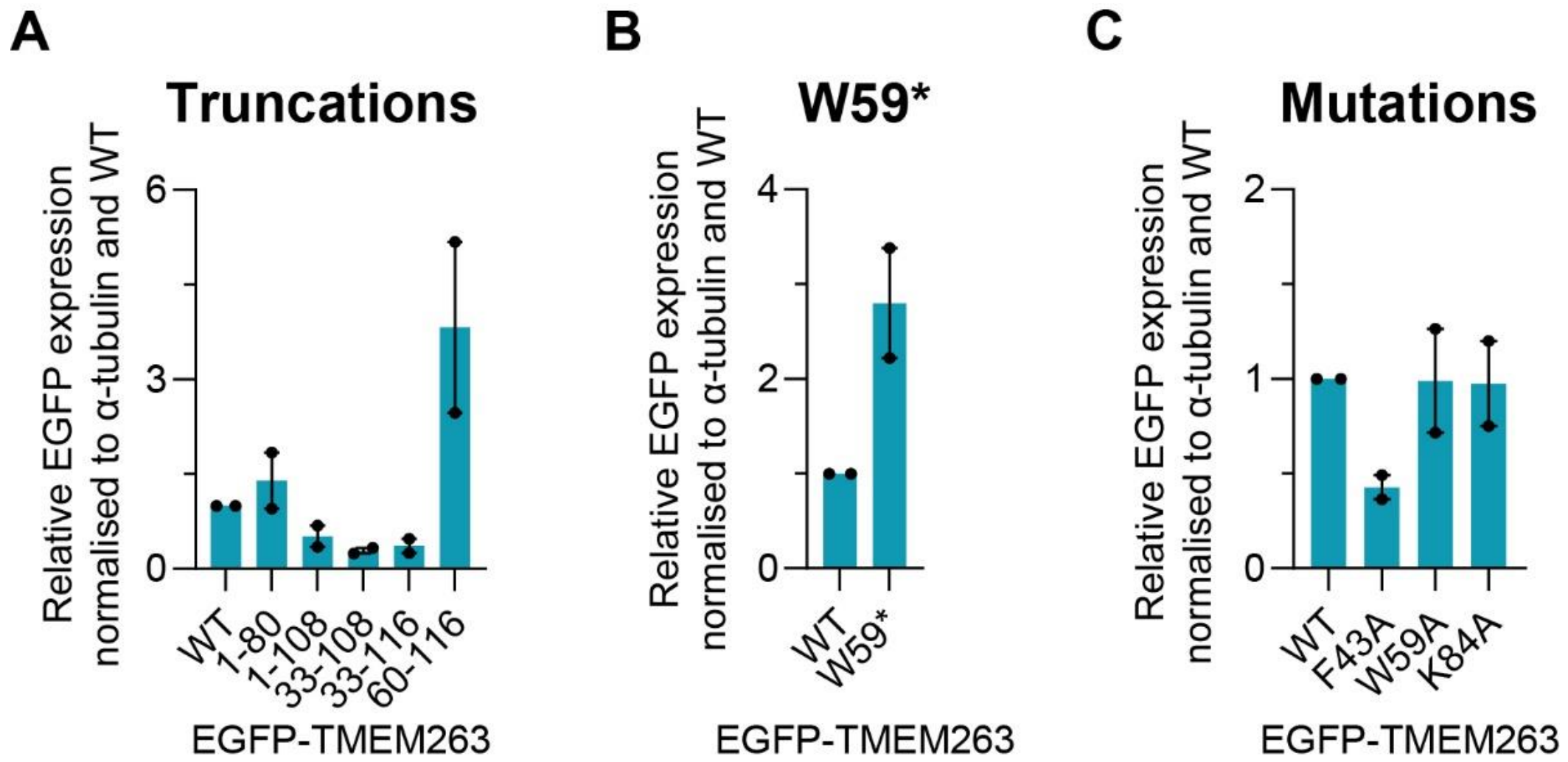

Rebuttal Figure 1: *HeLa cells were transfected with EGFP-TMEM263^{WT}, truncations and mutations. Quantification of blots showing the expression levels of EGFP-TMEM263 normalised to α-tubulin relative to EGFP-TMEM263^{WT} (n=2 for each experiment).*

4. Comment 17 (dose-response statistics): In Rebuttal Fig. 3, statistical significance across the control/low/medium/high EGFP-TMEM263 dose comparisons is inconsistent (e.g., LD size is significant for control-vs-high and low-vs-high but not control-vs-medium; LD number does not reach significance at any comparison). Please ensure the manuscript text describes this relationship with the same caution reflected in the rebuttal, rather than implying a uniformly significant dose-dependent trend.

*We thank the reviewer for pointing out this detail. The data shown in the rebuttal (dose-response statistics) come from experiments in steady-state condition (i.e. without oleic acid treatment). In this condition, the medium transfection level of HeLa cells with EGFP-TMEM263, which was used in the experiments in the main study, shows no statistical significance from the untransfected control cells in both LD size and number. In the manuscript, the data shown in **manuscript Fig. 2D and Fig. S2C** come from experiments in lipid-fed condition (i.e. with oleic acid treatment). In this condition, the LD size is statistically significantly higher in EGFP-TMEM263 transfected cells compared to untransfected controls cells, while LD number is not statistically different. Therefore the LD size is increasing significantly only when the cells are treated with oleic acid. We have modified the text to highlight that the effect shown in the main figures is present only after oleic acid treatment.*
