# Supplementary material for: Identification of TMEM263 as an ER-resident protein that controls early stages of lipid droplet biogenesis
Source: Nat Commun. 2026 Sep 28;17:11192. doi: 10.1038/s41467-026-78093-9 (PMC13714300; doi:10.1038/s41467-026-78093-9)
Supplement: Supplementary file 3 [file 41467_2026_78093_MOESM3_ESM.pdf]

Description of Additional Supplementary Files

Supplementary Data 1: Analysis of immunoprecipitation of εCOPI-GFP Vs GFP in HeLa cells (bottom-up proteomics)

Supplementary Movie 1: TMEM263 appears in nascent lipid droplets. HeLa cells transfected with EGFP-TMEM263 and mCherry-HPos were stained with LipidTOX and imaged live every 10 seconds for 18 minutes after oleic acid addition. The movie shows the formation of a LipidTOX-positive lipid droplet. An EGFP-TMEM263 positive circular structure appears first, then it becomes positive for mCherry-HPos and afterwards it is stained by LipidTOX. Scale bar is 2 μm.
