# Supplementary material for: Identification of TMEM263 as an ER-resident protein that controls early stages of lipid droplet biogenesis
Source: Nat Commun. 2026 Sep 28;17:11192. doi: 10.1038/s41467-026-78093-9 (PMC13714300; doi:10.1038/s41467-026-78093-9)
Supplement: Supplementary file 4 [file 41467_2026_78093_MOESM6_ESM.pdf]

nature portfolio

Corresponding author(s): Bernadette Carroll

Last updated by author(s): Sep 4, 2026

Reporting Summary

Nature Portfolio wishes to improve the reproducibility of the work that we publish. This form provides structure for consistency and transparency in reporting. For further information on Nature Portfolio policies, see our Editorial Policies and the Editorial Policy Checklist.

Statistics

For all statistical analyses, confirm that the following items are present in the figure legend, table legend, main text, or Methods section.

n/a	Confirmed	
☐	☒	The exact sample size (n) for each experimental group/condition, given as a discrete number and unit of measurement
☐	☒	A statement on whether measurements were taken from distinct samples or whether the same sample was measured repeatedly
☐	☒	The statistical test(s) used AND whether they are one- or two-sided<br>*Only common tests should be described solely by name; describe more complex techniques in the Methods section.*
☐	☐	A description of all covariates tested
☐	☒	A description of any assumptions or corrections, such as tests of normality and adjustment for multiple comparisons
☐	☒	A full description of the statistical parameters including central tendency (e.g. means) or other basic estimates (e.g. regression coefficient) AND variation (e.g. standard deviation) or associated estimates of uncertainty (e.g. confidence intervals)
☐	☒	For null hypothesis testing, the test statistic (e.g. F, t, r) with confidence intervals, effect sizes, degrees of freedom and P value noted<br>*Give P values as exact values whenever suitable.*
☒	☐	For Bayesian analysis, information on the choice of priors and Markov chain Monte Carlo settings
☒	☐	For hierarchical and complex designs, identification of the appropriate level for tests and full reporting of outcomes
☒	☐	Estimates of effect sizes (e.g. Cohen's d, Pearson's r), indicating how they were calculated

Our web collection on statistics for biologists contains articles on many of the points above.

Software and code

Policy information about availability of computer code

Data collection	N/A
Data analysis	N/A

For manuscripts utilizing custom algorithms or software that are central to the research but not yet described in published literature, software must be made available to editors and reviewers. We strongly encourage code deposition in a community repository (e.g. GitHub). See the Nature Portfolio guidelines for submitting code & software for further information.

Data

Policy information about availability of data

All manuscripts must include a data availability statement. This statement should provide the following information, where applicable:

- Accession codes, unique identifiers, or web links for publicly available datasets
- A description of any restrictions on data availability
- For clinical datasets or third party data, please ensure that the statement adheres to our policy

All data and reagents will be made available upon request. Source data are provided with this paper. The mass spectrometry proteomics data have been deposited to the ProteomeXchange Consortium via the PRIDE partner repository with the dataset identifier PXD083284.

April 2023

Research involving human participants, their data, or biological material

Policy information about studies with human participants or human data. See also policy information about sex, gender (identity/presentation), and sexual orientation and race, ethnicity and racism.

Reporting on sex and gender	N/A
Reporting on race, ethnicity, or other socially relevant groupings	N/A
Population characteristics	N/A
Recruitment	N/A
Ethics oversight	N/A

Note that full information on the approval of the study protocol must also be provided in the manuscript.

Field-specific reporting

Please select the one below that is the best fit for your research. If you are not sure, read the appropriate sections before making your selection.

[x] Life sciences [ ] Behavioural & social sciences [ ] Ecological, evolutionary & environmental sciences

For a reference copy of the document with all sections, see nature.com/documents/nr-reporting-summary-flat.pdf

Life sciences study design

All studies must disclose on these points even when the disclosure is negative.

Sample size	In cell based experiments, all experiments were performed with 3 biological replicates, with the exception of some western blot experiments that were performed with 2 biological replicates, the proteomic experiment that was performed with 4 biological replicates to ensure that all significant hits are recognised and the experiments with TMEM263 KO1/2+ that were performed with 4 biological replicates due to high variability within samples. For the fish experiments, a pilot experiment was performed to assess the number of total fish needed. For computational experiments, at least 5 repeats were done. Sample size is mentioned in figure legend.
Data exclusions	Data from 4 OA-fed tmem263 crispant fish were excluded from analyses (Fig.5F, H and Supplementary Fig. 6G) because the genotyping results were inconclusive. No data were excluded from the rest of the analyses.
Replication	For fish experiments, all attempts at replication were successful. In the majority of the experiments (cell based and computational), independent experiments are shown in the graphs to demonstrate the replication success. The number of independent repeats with similar results is the same as the number of replicates unless stated otherwise. For chi-squared tests this information is in the Source Data file.
Randomization	For cell based experiments, cells were seeded in wells of a plate and the next day the allocation into groups (OA treatment/ transfection) was done randomly in different wells.<br>For the fish experiments, fish were randomly allocated in to control or OA fed groups.
Blinding	Blinding was not relevant in the cell based experiments in this study because the majority of the data were analysed using macros or other codes. Regarding fish experiments, during image acquisition, the researcher knew which fish were injected or were WT, but did not know which of the injected fish were successful mutants. ROIs for image analysis were taken from the same region in every fish so blinding would not have altered the images acquired. For image analysis genotypes were blinded.

Reporting for specific materials, systems and methods

We require information from authors about some types of materials, experimental systems and methods used in many studies. Here, indicate whether each material, system or method listed is relevant to your study. If you are not sure if a list item applies to your research, read the appropriate section before selecting a response.

Materials & experimental systems

n/a	Involved in the study
☐	☒ Antibodies
☐	☒ Eukaryotic cell lines
☒	☐ Palaeontology and archaeology
☐	☒ Animals and other organisms
☒	☐ Clinical data
☒	☐ Dual use research of concern
☒	☐ Plants

Methods

n/a	Involved in the study
☒	☐ ChIP-seq
☒	☐ Flow cytometry
☒	☐ MRI-based neuroimaging

Antibodies

Antibodies used	Primary antibodies:<br>γCOP (Proteintech, 12393-1-AP, rabbit, 1:1000), ADRP/Perilipin-2 (PLIN2, Proteintech, 15294-1-AP, rabbit 1:400), TMEM263 (Invitrogen, PA5-70960, rabbit 1:200 & NovusBio, BP2-83690, rabbit, 1:200), Flag M2 (Sigma-Aldrich, F1804, mouse, 1:1000), Sec61β (Cell Signaling Technology, #14648, rabbit, 1:200), TGN46 (GeneTex, GTX74290, sheep, 1:2000), BSCL2/Seipin (Abnova, H00026580-A02, mouse, 1:200), LPCAT1 (Abcam, ab214034, rabbit, 1:250), LPCAT2 (Abcam, ab224244, rabbit, 1:250), HA.11 (Biolegend, #901573, mouse, 1:1000), GFP (Roche, 11814460001, mouse, 1:2000; Proteintech, PABG1, Rabbit, 1:2000), mCherry (Abcam, ab167453, rabbit, 1:2000), βCOP (gift from D. Stephens, University of Bristol, mouse, 1:500), α-tubulin (Sigma-Aldrich T9026, mouse, 1:1000), and GAPDH (Cell Signaling Technology, #97166, mouse, 1:2000).<br>Secondary antibodies:<br>Goat anti-rabbit 594 (A11037, Invitrogen, Thermo Fisher Scientific), donkey anti-rabbit 568 (A10042, Invitrogen, Thermo Fisher Scientific), goat anti-rabbit 488 (A11034, Invitrogen, Thermo Fisher Scientific), donkey anti-mouse 568 (A10037, Invitrogen, Thermo Fisher Scientific), and donkey anti-sheep 633 (A21100, Invitrogen, Thermo Fisher Scientific), goat anti-rabbit STAR RED (abberior, STRED-1002, 1:200) and goat anti-ms STAR ORANGE (abberior, STORANG-1001, 1:200), goat anti-mouse 680 (Invitrogen, Thermo Fisher Scientific, A21057, 1:20000) and goat anti-rabbit 800 (Invitrogen, Thermo Fisher Scientific, SA535571, 1:20000).
Validation	TMEM263 antibodies were validated in this study (Supplementary Fig. 3J, 6A)

Eukaryotic cell lines

Policy information about cell lines and Sex and Gender in Research

Cell line source(s)	All standard cell lines were obtained from collaborators (HeLa, MEFs, RPE1-hTERT) or ATCC (HEK293T).
Authentication	The HEK293T used in this study were authenticated by ATCC, no other cell lines were authenticated.
Mycoplasma contamination	All cell lines tested negative for mycoplasma.
Commonly misidentified lines (See ICLAC register)	N/A

Animals and other research organisms

Policy information about studies involving animals; ARRIVE guidelines recommended for reporting animal research, and Sex and Gender in Research

Laboratory animals	zebrafish, wild-type (injected with control or tmem263-targeted guides). Experimental animals were 6dpf
Wild animals	N/A
Reporting on sex	Gender was not considered during this study due to the age of the animals (one cell stage at guide RNA injection, and 5/6dpf at analysis)
Field-collected samples	N/A
Ethics oversight	Experiments were approved by the local ethics committee (the Animal Welfare and Ethical Review Body of the University of Bristol) and performed under UK Home Office project licence.

Note that full information on the approval of the study protocol must also be provided in the manuscript.

Plants

Seed stocks	N/A
Novel plant genotypes	N/A
Authentication	N/A
