# Supplementary material for: Identification of TMEM263 as an ER-resident protein that controls early stages of lipid droplet biogenesis
Source: Nat Commun. 2026 Sep 28;17:11192. doi: 10.1038/s41467-026-78093-9 (PMC13714300; doi:10.1038/s41467-026-78093-9)
Supplement: Supplementary file 5 [file 41467_2026_78093_MOESM7_ESM.zip › Source Data/Western blots.pdf]

Figure 1

B

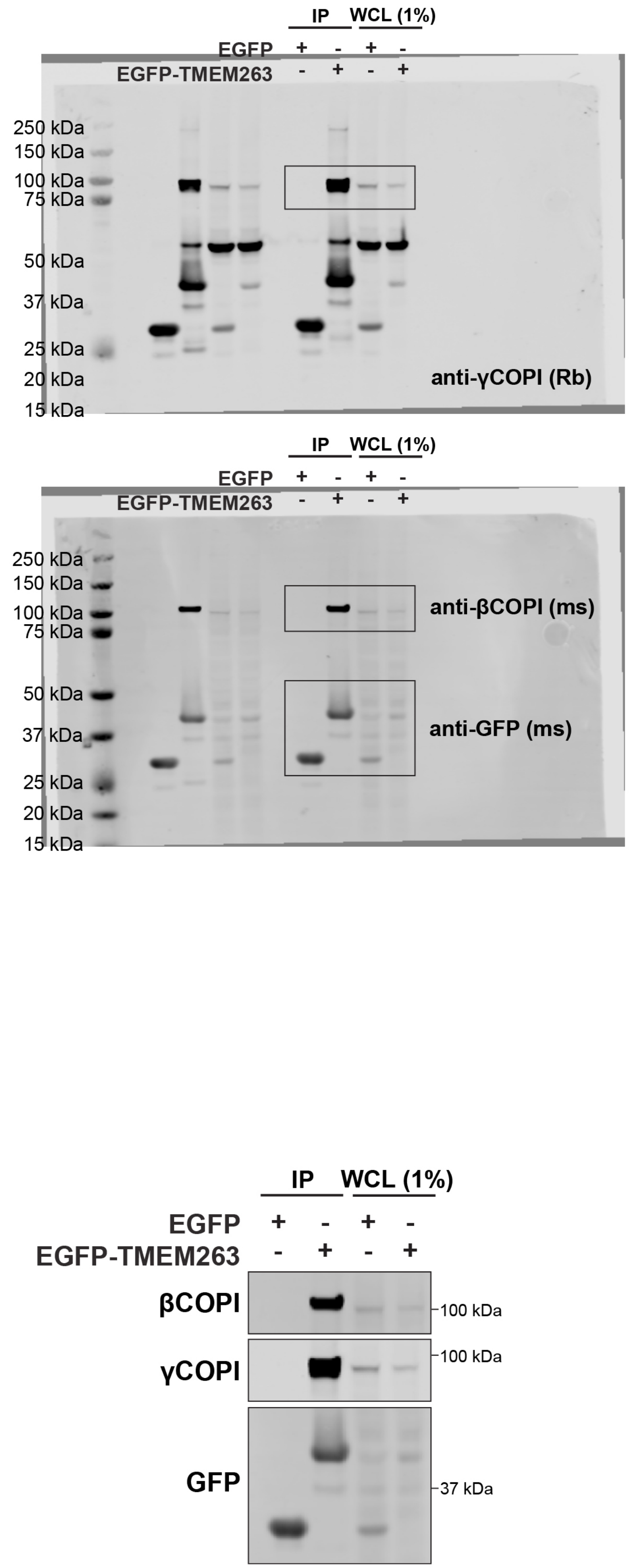

IP
WCL (1%)
EGFP
EGFP-TMEM263
250 kDa
150 kDa
100 kDa
75 kDa
50 kDa
37 kDa
25 kDa
20 kDa
15 kDa
anti-γCOPI (Rb)
anti-βCOPI (ms)
anti-GFP (ms)
βCOPI
γCOPI
GFP
100 kDa
37 kDa

E

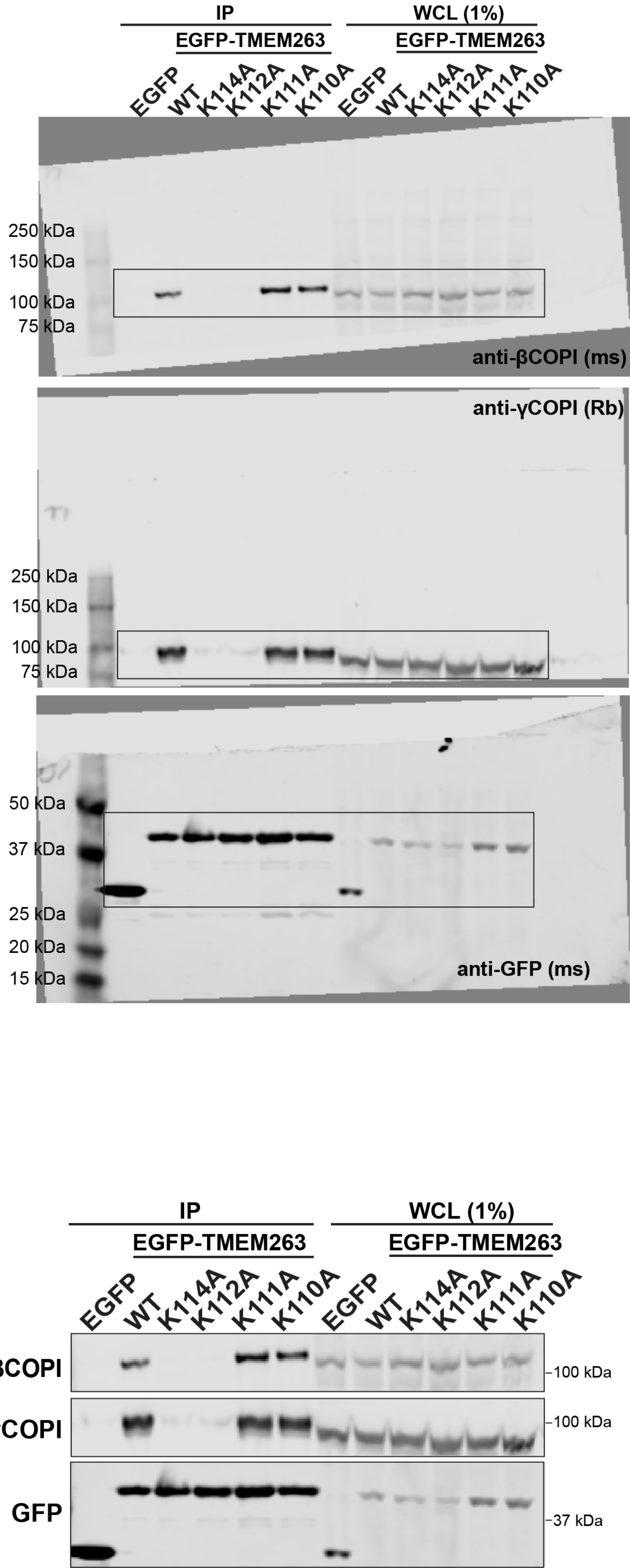

IP
WCL (1%)
EGFP-TMEM263
EGFP
WT
K114A
K112A
K111A
K110A
250 kDa
150 kDa
100 kDa
75 kDa
50 kDa
37 kDa
25 kDa
20 kDa
15 kDa
anti-βCOPI (ms)
anti-γCOPI (Rb)
anti-GFP (ms)
βCOPI
γCOPI
GFP
100 kDa
37 kDa

Figure S1

A

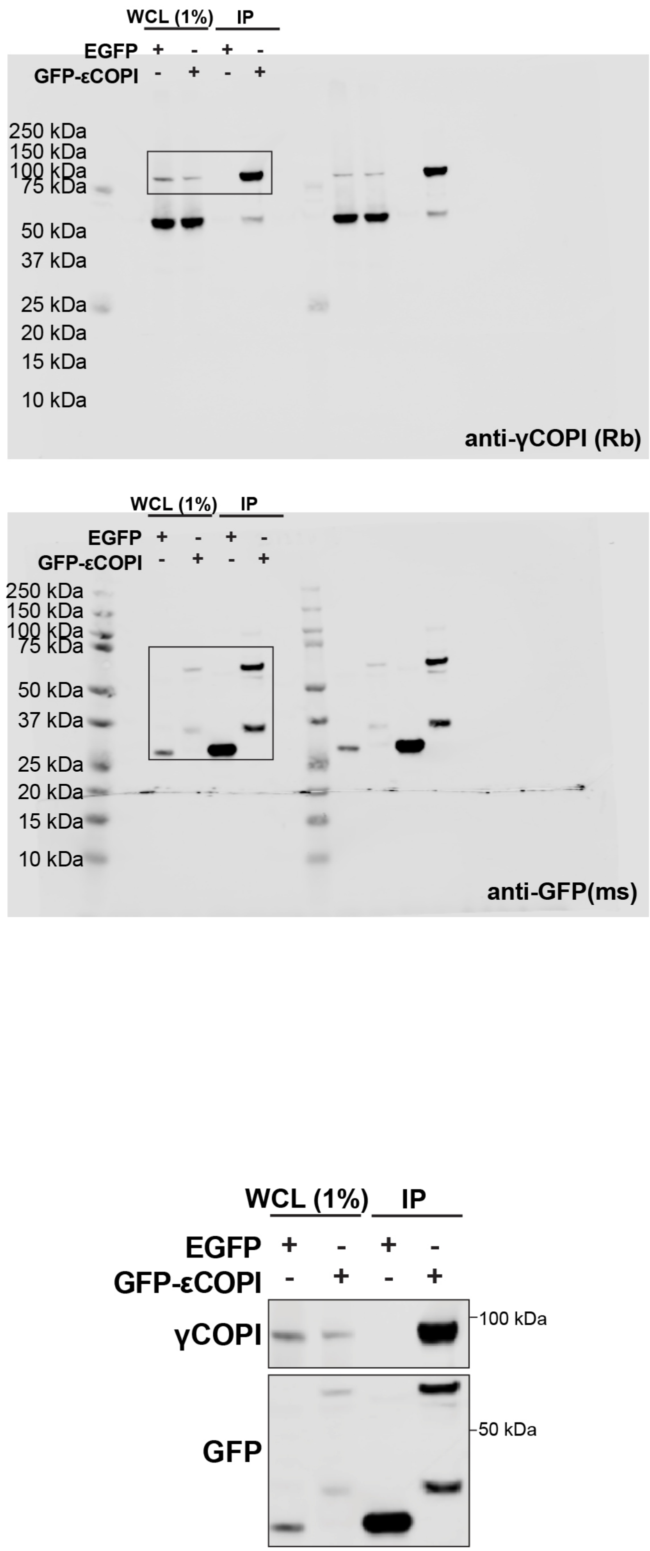
WCL (1%)
IP
EGFP + - + -
GFP-εCOPI - + - +
250 kDa
150 kDa
100 kDa
75 kDa
50 kDa
37 kDa
25 kDa
20 kDa
15 kDa
10 kDa
anti-γCOPI (Rb)
anti-GFP(ms)
γCOPI
GFP
100 kDa
50 kDa

D

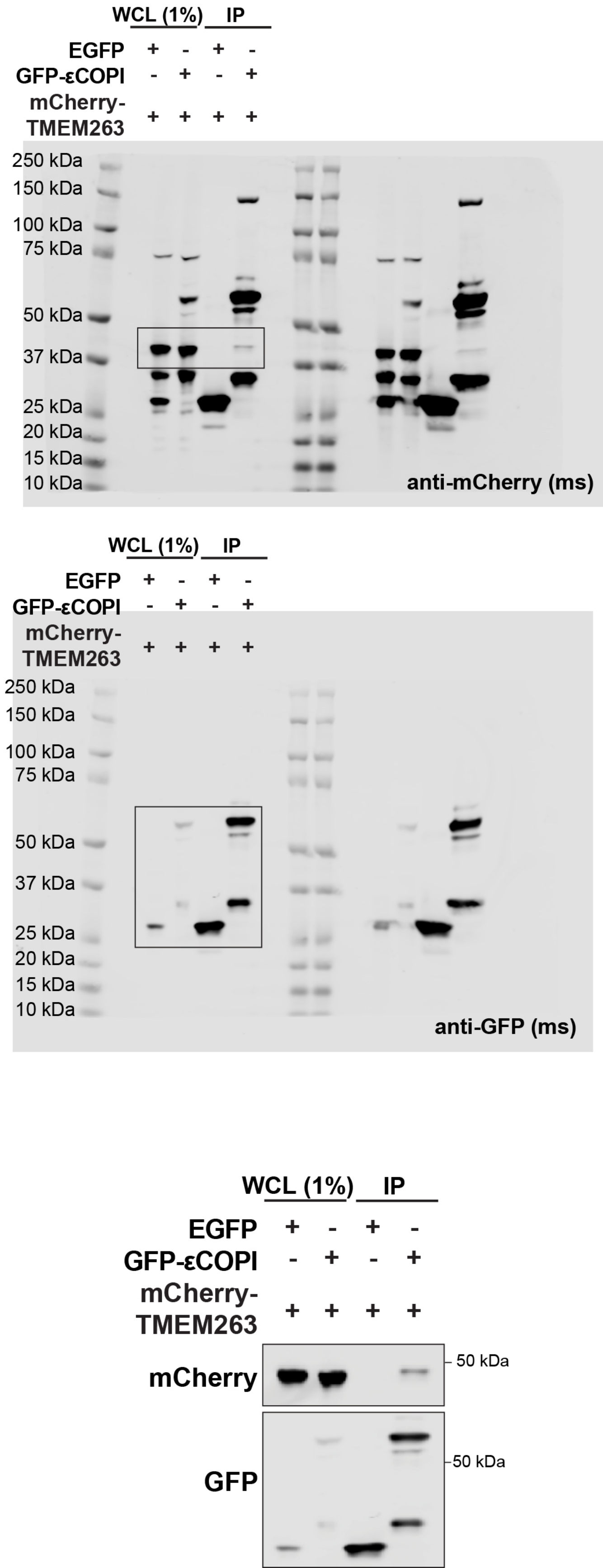
WCL (1%)
IP
EGFP + - + -
GFP-εCOPI - + - +
mCherry-TMEM263 + + + +
250 kDa
150 kDa
100 kDa
75 kDa
50 kDa
37 kDa
25 kDa
20 kDa
15 kDa
10 kDa
anti-mCherry (ms)
anti-GFP (ms)
mCherry
GFP
50 kDa

Figure S4

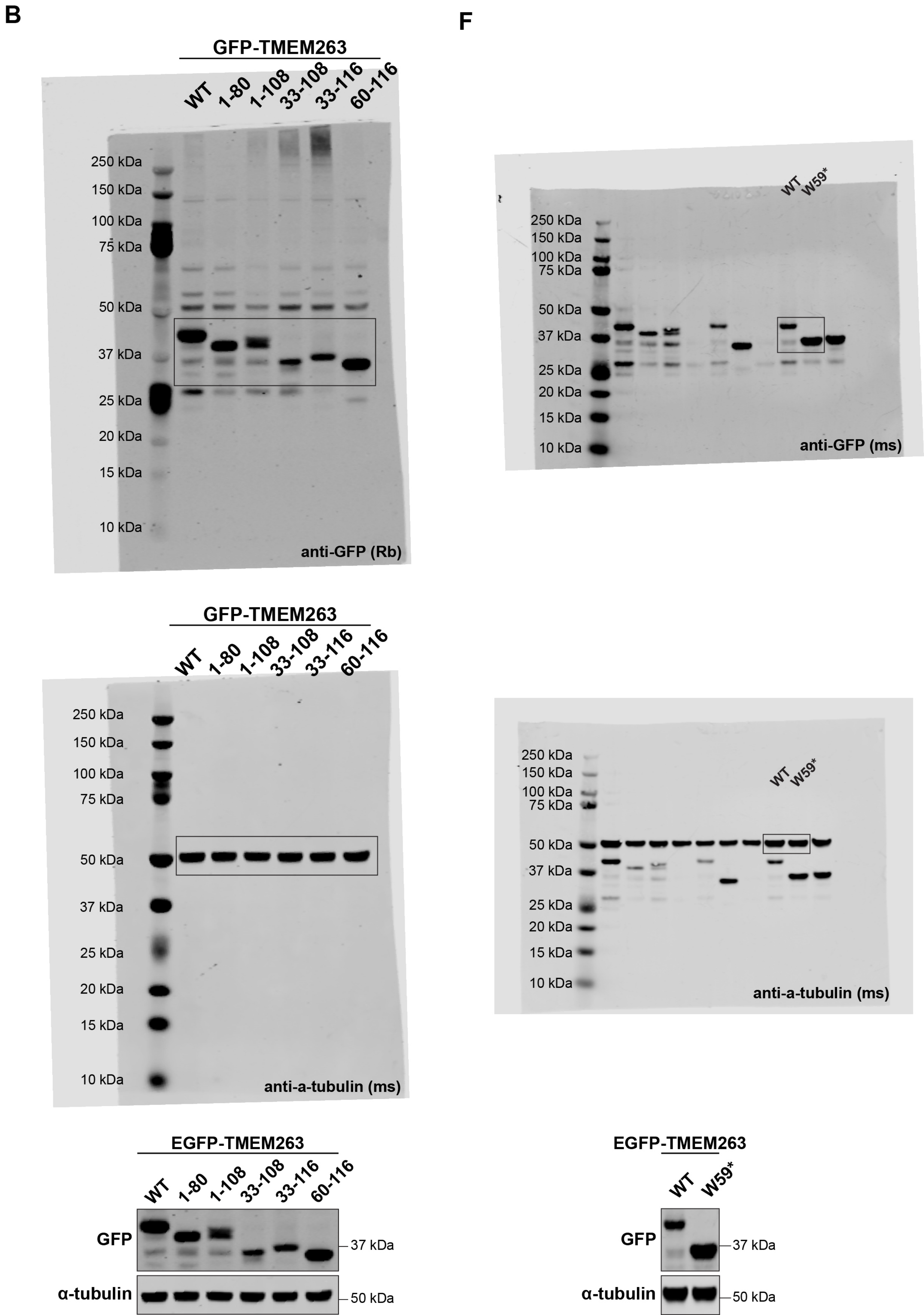
B
GFP-TMEM263
WT
1-80
1-108
33-108
33-116
60-116
250 kDa
150 kDa
100 kDa
75 kDa
50 kDa
37 kDa
25 kDa
20 kDa
15 kDa
10 kDa
anti-GFP (Rb)
GFP-TMEM263
WT
1-80
1-108
33-108
33-116
60-116
250 kDa
150 kDa
100 kDa
75 kDa
50 kDa
37 kDa
25 kDa
20 kDa
15 kDa
10 kDa
anti-a-tubulin (ms)
EGFP-TMEM263
WT
1-80
1-108
33-108
33-116
60-116
GFP
37 kDa
α-tubulin
50 kDa
F
WT
W59*
250 kDa
150 kDa
100 kDa
75 kDa
50 kDa
37 kDa
25 kDa
20 kDa
15 kDa
10 kDa
anti-GFP (ms)
250 kDa
150 kDa
100 kDa
75 kDa
50 kDa
37 kDa
25 kDa
20 kDa
15 kDa
10 kDa
WT
W59*
anti-a-tubulin (ms)
EGFP-TMEM263
WT
W59*
GFP
37 kDa
α-tubulin
50 kDa

Figure S5

C

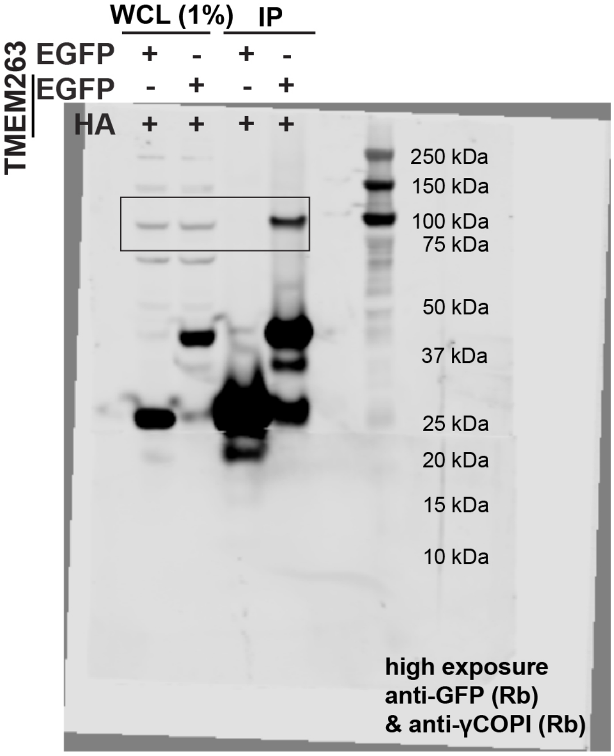
WCL (1%)
IP
TMEM263
EGFP + - + -
EGFP - + - +
HA + + + +
250 kDa
150 kDa
100 kDa
75 kDa
50 kDa
37 kDa
25 kDa
20 kDa
15 kDa
10 kDa
high exposure
anti-GFP (Rb)
& anti-γCOPI (Rb)

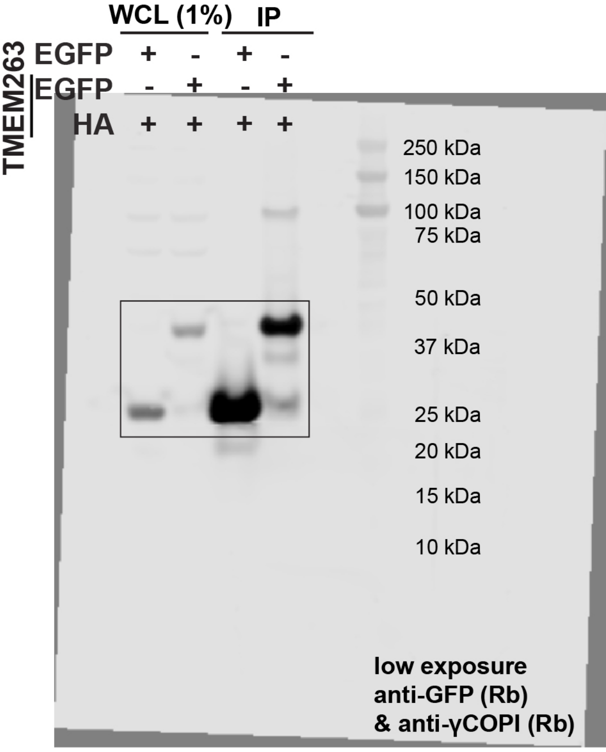
WCL (1%)
IP
TMEM263
EGFP + - + -
EGFP - + - +
HA + + + +
250 kDa
150 kDa
100 kDa
75 kDa
50 kDa
37 kDa
25 kDa
20 kDa
15 kDa
10 kDa
low exposure
anti-GFP (Rb)
& anti-γCOPI (Rb)

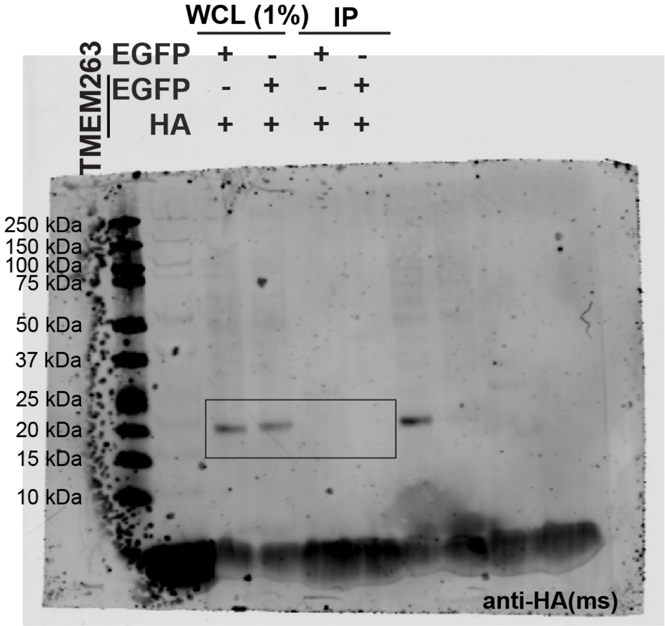
WCL (1%)
IP
TMEM263
EGFP + - + -
EGFP - + - +
HA + + + +
250 kDa
150 kDa
100 kDa
75 kDa
50 kDa
37 kDa
25 kDa
20 kDa
15 kDa
10 kDa
anti-HA(ms)

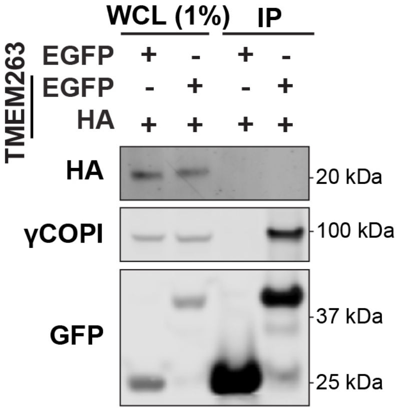
WCL (1%)
IP
TMEM263
EGFP + - + -
EGFP - + - +
HA + + + +
HA
γCOPI
GFP
20 kDa
100 kDa
37 kDa
25 kDa

D

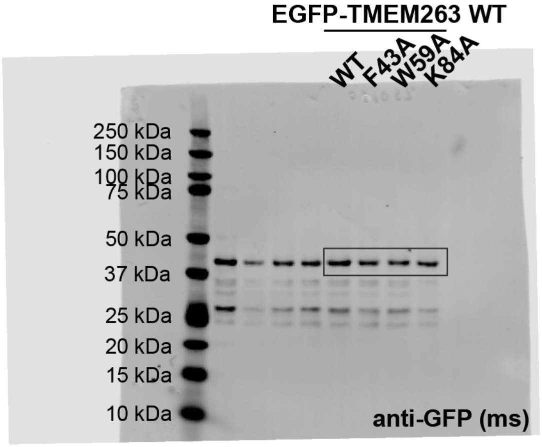
EGFP-TMEM263 WT
WT
F43A
W59A
K84A
250 kDa
150 kDa
100 kDa
75 kDa
50 kDa
37 kDa
25 kDa
20 kDa
15 kDa
10 kDa
anti-GFP (ms)

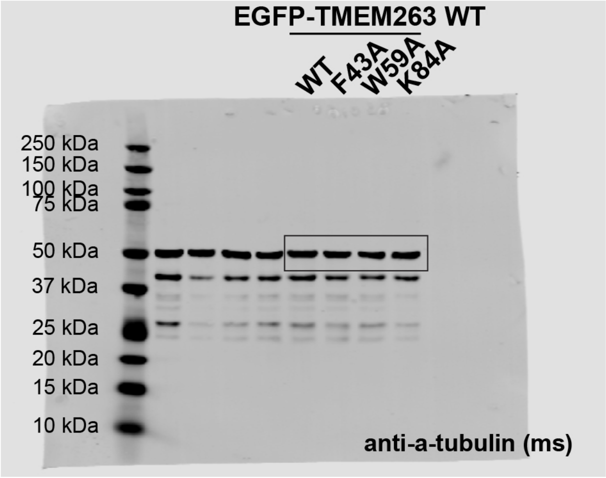
EGFP-TMEM263 WT
WT
F43A
W59A
K84A
250 kDa
150 kDa
100 kDa
75 kDa
50 kDa
37 kDa
25 kDa
20 kDa
15 kDa
10 kDa
anti-a-tubulin (ms)

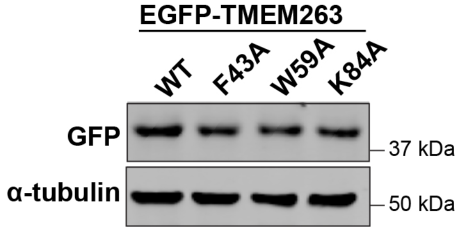
EGFP-TMEM263
WT
F43A
W59A
K84A
GFP
α-tubulin
37 kDa
50 kDa

Figure S6

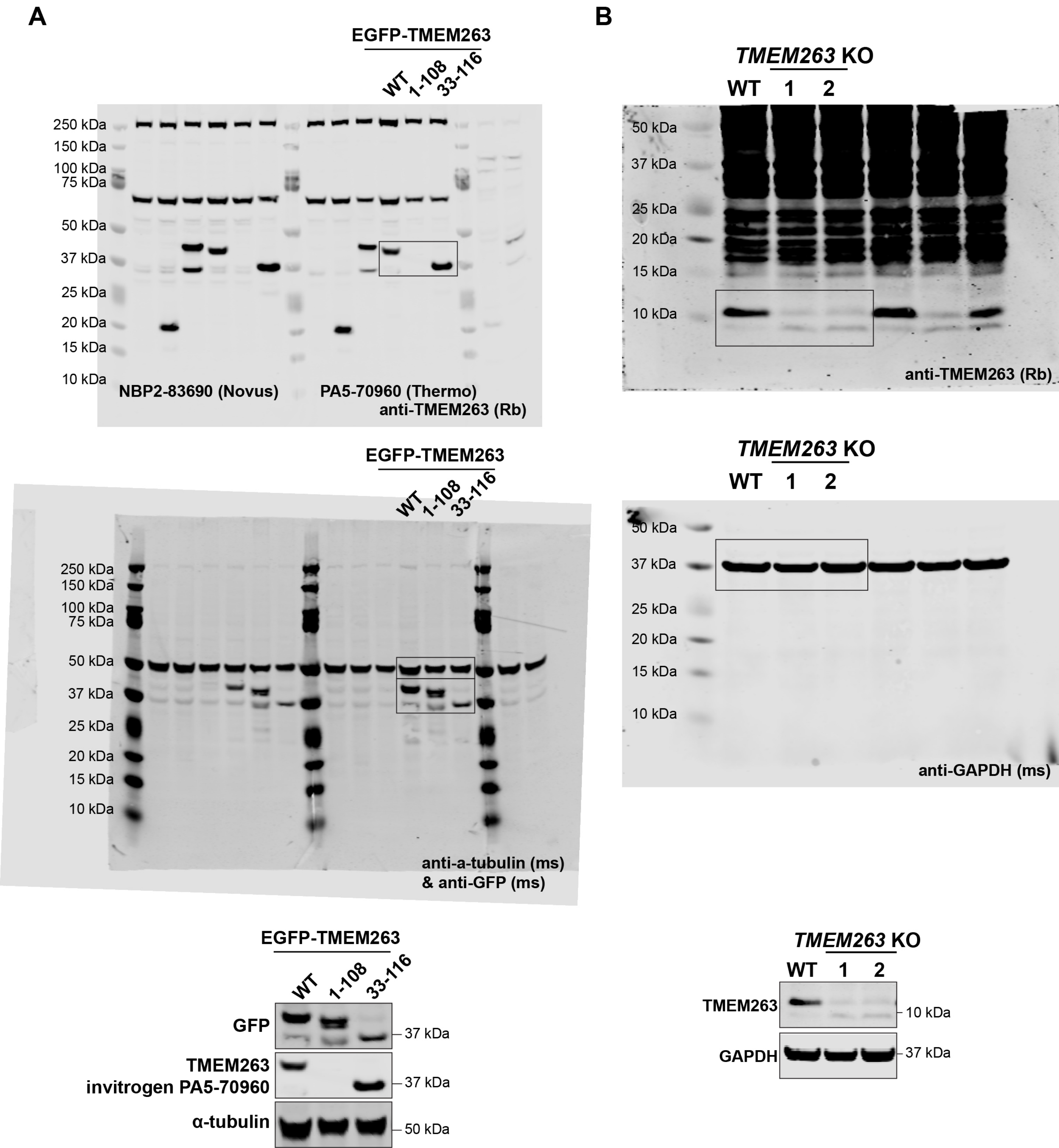
A
EGFP-TMEM263
WT
1-108
33-116
250 kDa
150 kDa
100 kDa
75 kDa
50 kDa
37 kDa
25 kDa
20 kDa
15 kDa
10 kDa
NBP2-83690 (Novus)
PA5-70960 (Thermo)
anti-TMEM263 (Rb)
EGFP-TMEM263
WT
1-108
33-116
anti-a-tubulin (ms)
& anti-GFP (ms)
EGFP-TMEM263
WT
1-108
33-116
GFP
TMEM263
invitrogen PA5-70960
α-tubulin
37 kDa
37 kDa
50 kDa
B
TMEM263 KO
WT
1
2
anti-TMEM263 (Rb)
TMEM263 KO
WT
1
2
anti-GAPDH (ms)
TMEM263 KO
WT
1
2
TMEM263
GAPDH
10 kDa
37 kDa
